# Role of the Mobile Active Site Flap in IMP Dehydrogenase Inhibitor Binding

**DOI:** 10.1021/acsinfecdis.4c00636

**Published:** 2025-01-29

**Authors:** Xingyou Wang, Masha M. Rosenberg, Youngchang Kim, Natalia Maltseva, Gregory D. Cuny, Andrzej Joachimiak, Petr Kuzmič, Lizbeth Hedstrom

**Affiliations:** † Department of Chemistry, Brandeis University, Waltham, Massachusetts 02454, United States; ‡ Department of Biology, Brandeis University, Waltham, Massachusetts 02454, United States; § Center for Structural Biology of Infectious Diseases, Consortium for Advanced Science and Engineering, University of Chicago, Chicago, Illinois 60667, United States; ∥ The Structural Biology Center, X-ray Science Division, Argonne National Laboratory, Lemont, Illinois 60439, United States; ⊥ Department of Pharmacological and Pharmaceutical Sciences, College of Pharmacy, University of Houston, Houston, Texas 77204, United States; # Department of Biochemistry and Molecular Biology, University of Chicago, Chicago, Illinois 60367, United States; ∇ BioKin Ltd., Watertown, Massachusetts 02472, United States

**Keywords:** IMPDH, inhibitor selectivity, antibiotics, enzyme kinetics, presteady-state kinetics, STD-NMR

## Abstract

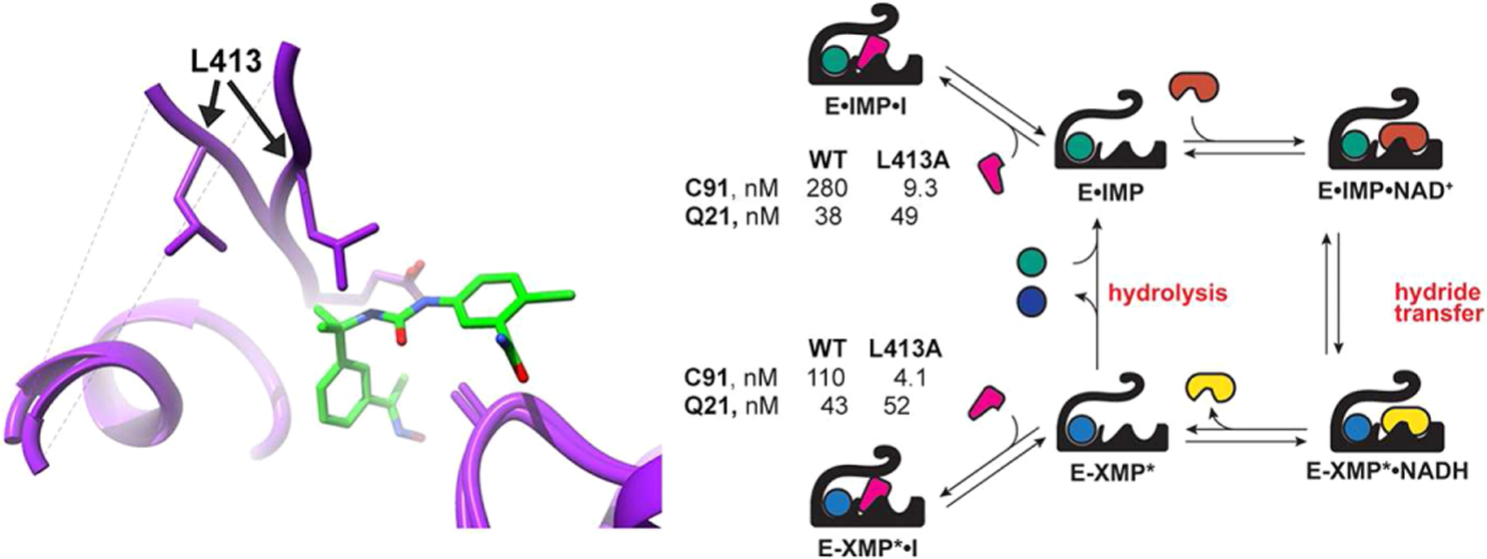

Inosine 5′-monophosphate dehydrogenase (IMPDH) is a promising antibiotic target. This enzyme catalyzes the NAD-dependent oxidation of inosine 5′-monophosphate (IMP) to xanthosine 5′-monophosphate (XMP), which is the rate-limiting step in guanine nucleotide biosynthesis. Bacterial IMPDH-specific inhibitors have been developed that bind to the NAD^+^ site. These inhibitors display varied affinities to different bacterial IMPDHs that are not easily rationalized by X-ray crystal structures of enzyme–inhibitor complexes. Inspection of X-ray crystal structures of 25 enzyme–inhibitor complexes, including 10 newly described, suggested that a mobile active site flap may be a structural determinant of inhibitor potency. Saturation transfer difference NMR experiments also suggested that the flap may contact the inhibitors to varying extents in different IMPDHs. Flap residue Leu413 contacted some inhibitors but was not structured in the crystal structures of other inhibitor complexes. The substitution of Leu413 with Phe or Ala in *Bacillus anthracis* IMPDH had inhibitor-selective effects, suggesting residue 413 could be a structural determinant of affinity. Curiously, the Ala substitution increased the potency of most inhibitors, even those that contacted Leu413 in the crystal structures. Presteady-state and steady-state kinetics experiments showed that the Leu413Ala substitution had comparable effects on inhibitor binding to the noncovalent E·IMP complex and the covalent intermediate E-XMP*, suggesting that the flap had similar interactions in both complexes. These results demonstrate that contacts do not necessarily indicate favorable interactions, and poorly structured mobile regions should not be discounted when assessing binding determinants.

Inosine 5′-monophosphate dehydrogenase (IMPDH) catalyzes the oxidation of inosine 5′-monophosphate (IMP) to xanthosine 5′-monophosphate (XMP) with concomitant reduction of NAD^+^ to NADH. This is the rate-limiting step of guanine nucleotide biosynthesis.^1^ The inhibition of IMPDH decreases the guanine nucleotide pool, thereby suppressing the cell proliferation. IMPDH is a validated drug target for immunosuppressive,^2^ cancer,^3^ and antiviral^4^ chemotherapy and a promising target for the treatment of infectious diseases,^5^ where new drugs are urgently needed to fight against the threat of antibiotic resistance.

The NAD^+^ binding sites of bacterial and mammalian IMPDHs are highly diverged.^6^ Our laboratory and others have exploited these differences to develop several series of selective inhibitors of bacterial IMPDHs, many of which display antibacterial activity.^7−14^ The structural determinants for the selectivity for bacterial over mammalian IMPDHs are revealed in the many E·IMP·I crystal structures of IMPDHs from *Bacillus anthracis* (*Ba*), *Campylobacter jejuni* (*Cj*), *Cryptosporidium parvum* (*Cp*), *Clostridium perfringens* (*Clp*), and *Mycobacterium tuberculosis* (*Mtb*) deposited in the Protein Data Bank (PDB). IMPDHs are square planar homotetramers with 4 active sites. The cofactor binding site is formed by residues of two subunits.^6^ These inhibitors are composed of two aromatic rings connected by an amide, urea, or tetrazole linker, and all adopt similar binding modes (Figure 1A–C). The ”leftside” aromatic ring (L-ring) binds in the nicotinamide subsite where it stacks with the base of IMP and interacts with Gly392 and Met397. The linker interacts with Ala253 and forms hydrogen bonds with the carboxylate of Glu416 while the “rightside” aromatic ring (R-ring) binds in the adenine subsite where it stacks with Tyr445′ (*Ba*IMPDH numbering; prime denotes residue from the adjacent monomer). Importantly, the interactions with Ala253, Glu416, and Tyr445′ account for the selectivity versus human IMPDHs.^15^ These residues are found in IMPDHs from many other pathogens, including *Streptococcus pyogenes* (*Sp*), *Staphylococcus aureus* (*Sa*), *Francisella tularensis* (*Ft*), and *Borrelia burgdorferi* (*Bb*) (Figure S1). The inhibitor binding sites are highly conserved in these enzymes, yet the structure–activity relationships (SARs) for inhibition vary between enzymes.^6^ For example, inhibitor **P32** displayed similar affinities for *Ba-* and *Clp*IMPDH (*K*_i,app_ = 47 and 34 nM, respectively) while **C91** was a 10-fold more potent inhibitor of *Ba*IMPDH, even though the enzyme–inhibitor contacts are conserved (Figure 1C).^6^ The structural features that determine the varying SAR of inhibition are not readily apparent in the crystal structures of the E·IMP·I complexes. Understanding the structural basis of the SARs of inhibition would greatly facilitate the development of IMPDH-targeted antibiotics.

**Figure 1:**
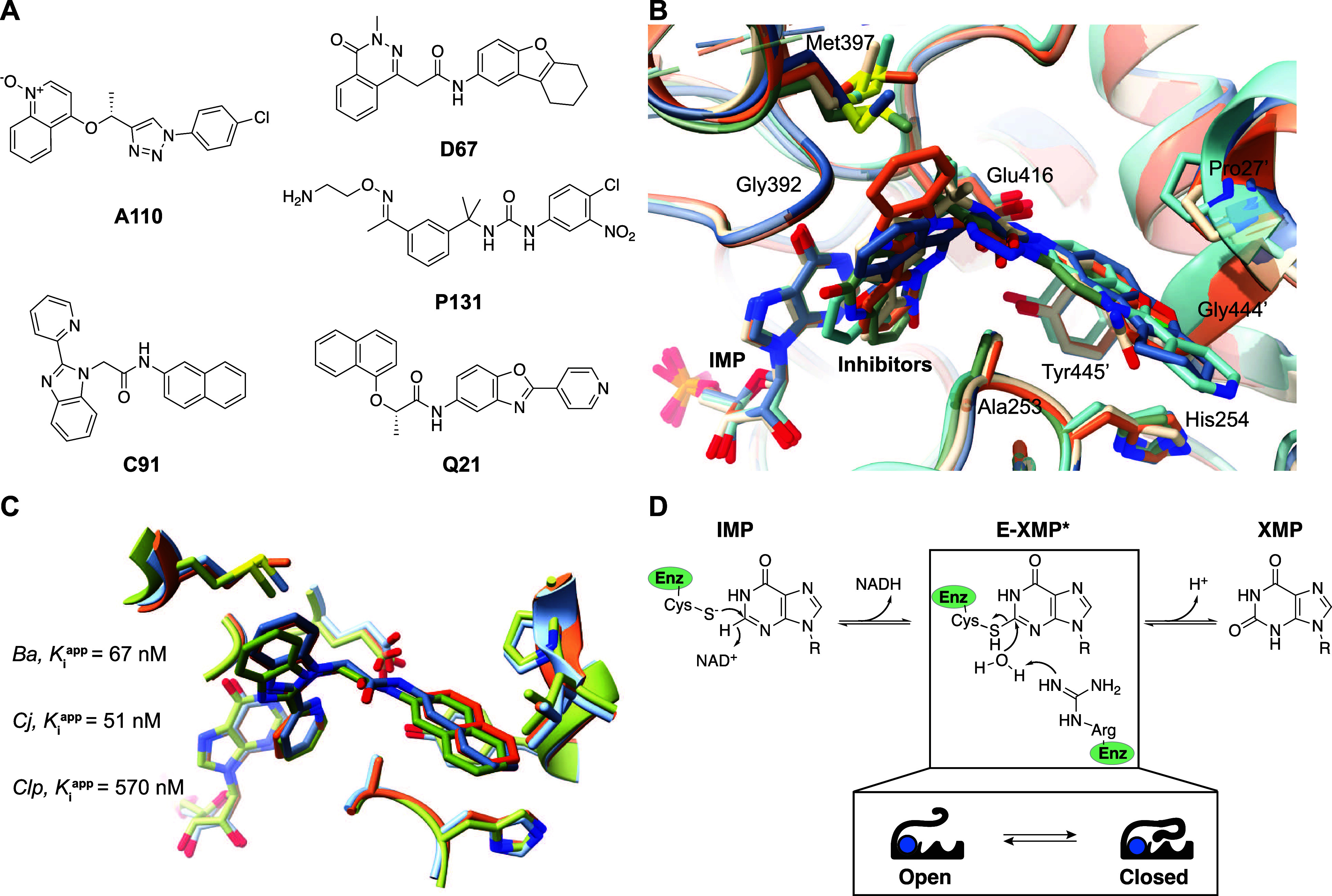
Selective inhibitors of bacterial IMPDHs. (A) Representative inhibitors of bacterial IMPDHs from five series (**A**, **C**, **P**, **D**, **Q**) with different scaffolds. (B) Overlay of *Ba*IMPDH with different inhibitors bound. The inhibitors adopt similar conformations in the cofactor site. Green: **A110** (PDB: 4MYA); orange: **C91** (PDB: 4MY9); deep blue: **D67** (PDB: 4QM1); beige: **P32** (PDB: 4MYX); cyan: **Q21** (PDB: 4MY8). (C) Overlay of *Ba-*, *Cj-*, and *Clp*IMPDH structures with **C91** bound. Residues making direct contacts with **C91** are shown. The *K*_i,app_ values are from ref (6). Colors are designated as follows: *Ba*IMPDH: orange; *Cj*IMPDH: green; and *Clp*IMPDH: blue. **C91** is colored a darker shade of the corresponding protein color. (D) Mechanism of IMPDH catalysis. A conformational change in E-XMP* is required for hydrolysis. **R**: ribose 5′-monophosphate.

A mobile active site flap (residues 381–421 in *Ba*IMPDH) plays a pivotal role in catalysis and cofactor/inhibitor binding and may also be the source of the varied SAR of inhibition. The IMPDH reaction begins with the sequential addition of IMP and NAD^+^. The flap is not observed in E·IMP and becomes partially structured in the presence of cofactor.^6,7^ The active site Cys308 attacks IMP and the hydride is transferred to NAD^+^, forming the E-XMP* covalent intermediate (Figure 1D). NADH departs, then the flap moves into the vacant cofactor site to form the closed conformation. Potassium activates IMPDH by promoting this conformational change.^16^ The closed conformation positions Arg404-Tyr405 to activate water for the hydrolysis of E-XMP*, producing XMP and the free enzyme.

Although structures are only available for E·IMP complexes, inhibitors can bind to both E·IMP and E-XMP*, with at least some inhibitors preferring E-XMP*.^17^ As observed with the cofactor, part of the flap becomes structured when inhibitors bind. The visible segments include three key inhibitor-interacting residues: Gly392, Met397 and Glu416. The flap appears to be highly dynamic and may transiently interact with the inhibitors in a manner not captured in the crystal structures. These observations suggest two explanations for the varied SAR of inhibition: (1) Perhaps the interactions that account for varying SAR are only manifest in E-XMP*; or (2) Perhaps the flap transiently contacts the inhibitors.

We set out to explore how the dynamic flap affects inhibitor binding and determine whether the E·IMP·I complexes are good models for E-XMP*·I complexes. Here we report 10 additional E·IMP·I crystal structures that further illustrate the varied conformations of the flap both within a given E·IMP·I complex and between different bacterial IMPDHs. We also report saturation transfer difference (STD) NMR experiments that reveal differences in inhibitor binding modes among bacterial IMPDHs. Presteady-state and steady-state studies were performed, showing that mutations of flap residue Leu413 have inhibitor-selective effects that are similar for both E·IMP·I and E-XMP*·I, suggesting that the flap has similar interactions in both complexes. However, the crystal structures were poor predictors of how an inhibitor was affected by the mutation, suggesting that the distribution of flap conformations in the crystal may not reflect that in solution.

## Results and Discussion

### Flap May Interact Transiently with Inhibitors

**Figure 2:**
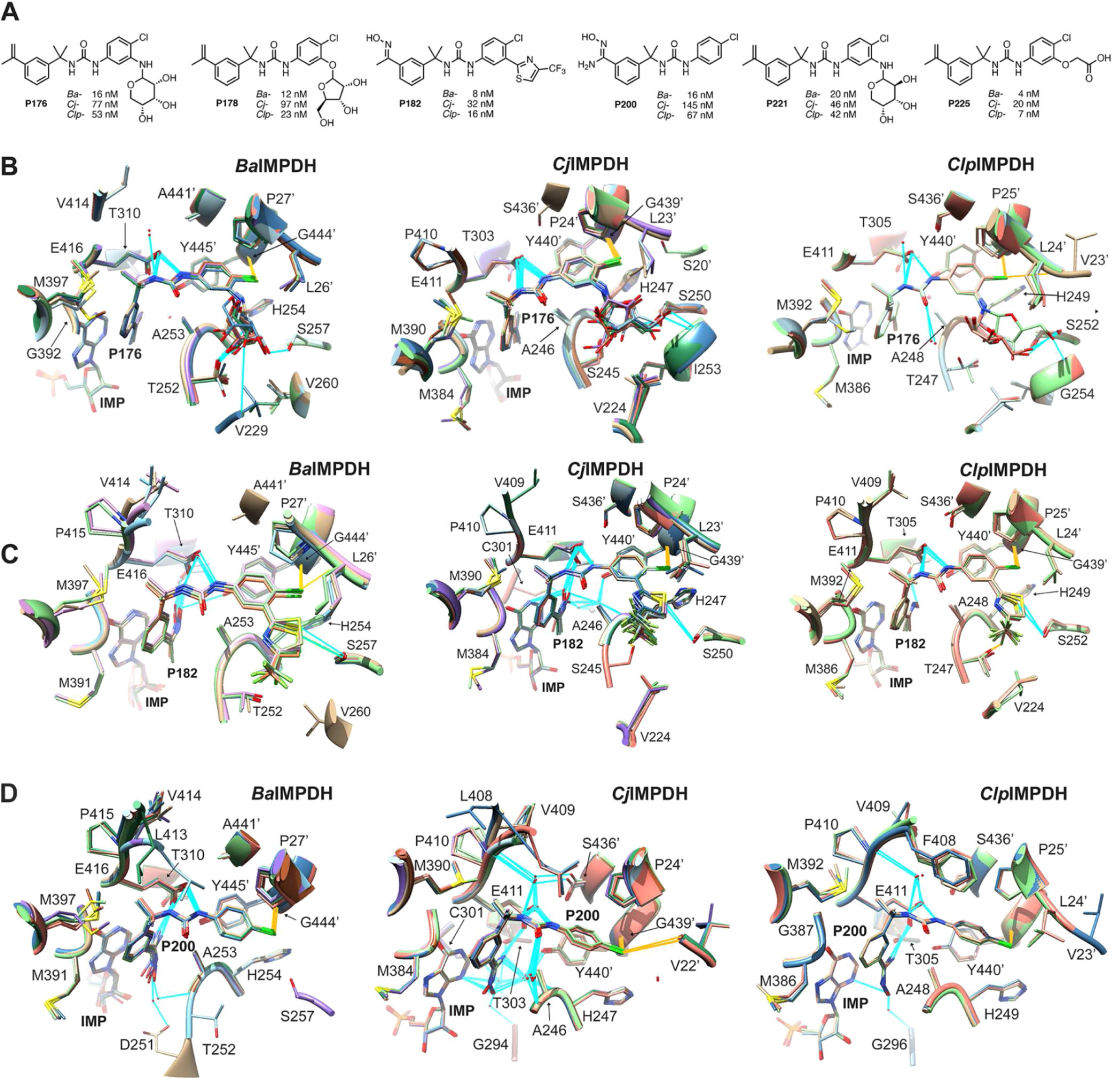
Structures of inhibitor complexes. (A) Inhibitor structures. The values of *K*_i,app_ are listed. (B) Structures of E·IMP·**P176** complexes. The asymmetric units of ***B***a- (PDB: 7MTX), *Cj-* (PDB: 5URQ) and *Clp*IMPDH (PDB: 5UWX) contained 8, 8, and 4 active sites, respectively. The IMP containing subunits were overlaid for each enzyme. Hydrogen bonds are depicted in cyan, and halogen bonds are gold. (C) Structures of E·IMP·**P182** complexes. The asymmetric units of *Ba-* (PDB: 5UUV), *Cj-* (PDB: 5UQH), and *Clp*IMPDH (PDB: 5UZE) contained 4, 8, and 4 active sites, respectively. (D) Structures of E·IMP·**P200** complexes. The asymmetric units of *Ba-* (PDB: 5UUZ), *Cj-* (PDB: 5UQG), and *Clp*IMPDH (PDB: 5UZS) contained 8, 8, and 4 active sites, respectively. Panels (B–D) were rendered in UCSF Chimera.^18^

We analyzed enzyme–inhibitor interactions in 10 crystal structures of E·IMP·I complexes from *Cj-* and *Clp*IMPDHs to investigate the structural determinants of inhibitor potency and selectivity (Tables S1 and S22). These complexes included 6 inhibitors from the **P** series, which contain an isopropylurea linker between the “leftside” L-ring and “rightside” R-ring phenyl groups: **P176**, **P178** (*Clp*IMPDH only), **P182**, **P200**, **P221** (*Clp*IMPDH only), and **P225** (Figure 2A). Structures of **P176**, **P182**, and **P200** have previously been reported with *Ba*IMPDH.^9^ All of the inhibitors contain an R-ring p-Cl that increased potency in *Ba*IMPDH.^8,9^**P176**, **P178**, and **P221** contain sugar substituents designed to interact with the adenosine ribose binding site. These compounds are freely equilibrating mixtures of pyranoses and furanoses.^9^ The values of *K*_i,app_ for these inhibitors ranged from 7 to 145 nM.

The resolutions of the 10 crystal structures varied from 1.85 to 2.73 Å. *Cj*IMPDH crystallized with 3–8 active sites in the asymmetric unit while all of the *Clp*IMPDH crystals contained 4 active sites. The IMP site is conserved among IMPDHs and IMP bound essentially identically in *Ba-*, *Cj-*, and *Clp*IMPDH (Figures 2B–D and S2). The inhibitor binding sites of *Cj-*, *Clp-*, and *Ba*IMPDH are also highly conserved. Only four substitutions were observed in residues that contact the inhibitors: (i) Thr252 is Ser in *Cj*IMPDH; the Thr252 side chain interacted with R-ring substituents, e.g., forming hydrogen to sugars or hydrophobic contacts with trifluoromethyl of **P182**. (ii) Val260 is Ile in *Cj*IMPDH; the Val260 side chain interacted with some R-group substituents, e.g., the trifluoromethyl group of **P182**. (iii) Leu413 is Phe in *Clp*IMPDH; this residue was usually not structured, but did interact with the linker in some inhibitors. (iv) Ala441′ is Ser in both *Cj-* and *Clp*IMPDH; the Ala441 side chain interacted with the R-ring in some inhibitors. Despite these differences, only two inhibitors, **P178** and **P200**, displayed greater than a 5-fold difference in affinity across the three enzymes.

The L-ring, R-ring, p-Cl, and isopropylurea linker made similar interactions in all of the complexes of all three enzymes (Figures 2B–D and S2). Hydrophobic interactions were observed between the L-rings and the IMP base, and Gly392 and the side chain of Met397 while the R-rings interacted with side chains of Tyr445′, His254, and Pro27′. The urea NHs formed hydrogen bonds to the Glu416 carboxylate, while a linker methyl group made hydrophobic contacts with Cβ and Cγ of Glu416. The urea carbonyl interacted with the Ala253 side chain. In addition, the R-ring p-Cl formed halogen bonds with the carbonyl of Gly444′ and C-X/π interactions with the imidazole of His254. These interactions were observed in almost every individual active site.

In contrast, interactions with the L- and R-ring substituents varied in individual active sites of a given crystal structure, obscuring potential determinants of enzyme selectivity. In **P176**, **P178**, and **P221**, this variation may have derived from inhibitor heterogeneity since these compounds were mixtures of pyranoses and furanoses. The resolution of these structures was not sufficient to unambiguously define the sugar conformations. **P176** was modeled as pyranose in the *Ba*IMPDH complex, with distinct conformations and patterns of hydrogen bonds in the individual active sites (Figure 2B). The side chains of the surrounding residues, Val229, Thr252, and Met397, were also found in different conformations. **P176** was modeled as a furanose in both *Cj-* and *Clp*IMPDH complexes. The sugar displayed 6 hydrogen bonding configurations in the 8 active sites of the *Cj*IMPDH crystal structures, with accompanying changes in the conformations of the surrounding residues (Figure 2B). Only two sugar conformations were observed in *Clp*IMPDH, with similarly little variation in the surrounding residues. Strikingly, interactions with the flap displayed the greatest variability in all three enzymes. The contacts between the inhibitor and flap also displayed considerable variation in the **P178** and **P221** complexes (Figure S2A,B).

Variations were also observed in the binding modes of **P182** and **P200** (Figure 2C,D). In **P182**, the thiazole ring and trifluoromethyl displayed varying orientations in each active site of all three enzymes, accompanied by changes in hydrogen bonding between the thiazole S and Ser257 hydroxyls as well as hydrophobic contacts with Thr252 and Val260. Distinct conformations and hydrogen bonding patterns were also observed for the L-ring oxime. The oxime hydroxyl formed hydrogen bonds to Glu416, Thr310, and Tyr445′ in varying combinations in the individual active sites of *Ba-* and *Cj*IMPDH, but only hydrogen bonds to Glu416 were observed with *Clp*IMPDH. The oxime hydroxyl also formed hydrogen bonds to IMP N1 and water in *Cj*IMPDH. Contacts were observed between **P182** and the Val414 Cα and Pro415 Cα in some but not all active sites of all three enzymes.

Only the hydroxyamidine group can rotate in **P200**, so these complexes displayed much less variability than the other inhibitors (Figure 2D). The hydroxyamidine NH_2_ formed hydrogen bonds to the Glu416 carboxylate in all of the active sites of all three enzymes. The hydroxyl made 2 water-mediated hydrogen bonds to protein in the 8 active sites of the *Ba*IMPDH structure. Two hydrogen bonds were observed in the 4 active sites of the *Clp*IMPDH structure, one water-mediated to the protein and the other direct to N3 of IMP. In contrast, the higher resolution of *Cj*IMPDH complex (2.0 versus 2.5 Å and 2.4 Å for *Ba-* and *Clp*IMPDH, respectively) revealed 31 hydrogen bonds to the NHOH groups in the 8 active sites in the asymmetric unit, including 5 to N3 of IMP, 2 directly to enzyme residues, and 24 to water. This apparent increased solvation of the active site of *Cj*IMPDH might contribute to the lower affinity of inhibitor binding, as has been proposed for other bacterial IMPDH inhibitors.^10^ The conformation of the flap once again displayed considerable heterogeneity both within and between structures, despite the relative rigidity of **P200**. The side chain of Leu413 contacted **P200** in 2 of 8 active sites in both *Ba-* and *Cj*IMPDH. This residue is Phe in *Clp*IMPDH, where it contacts the inhibitor in all four active sites. In contrast, both flap and inhibitor conformations were uniform in the *Cj-* and *Clp*IMPDH complexes with the most potent inhibitor **P225** (Figure S2C). Leu413 did not contact **P225** in any of these complexes. In aggregate, these observations suggest that the flap is dynamic and may contribute to inhibitor potency and SAR via transient interactions of Leu413, Val414, and Pro415, and perhaps other residues.

**Figure 3:**
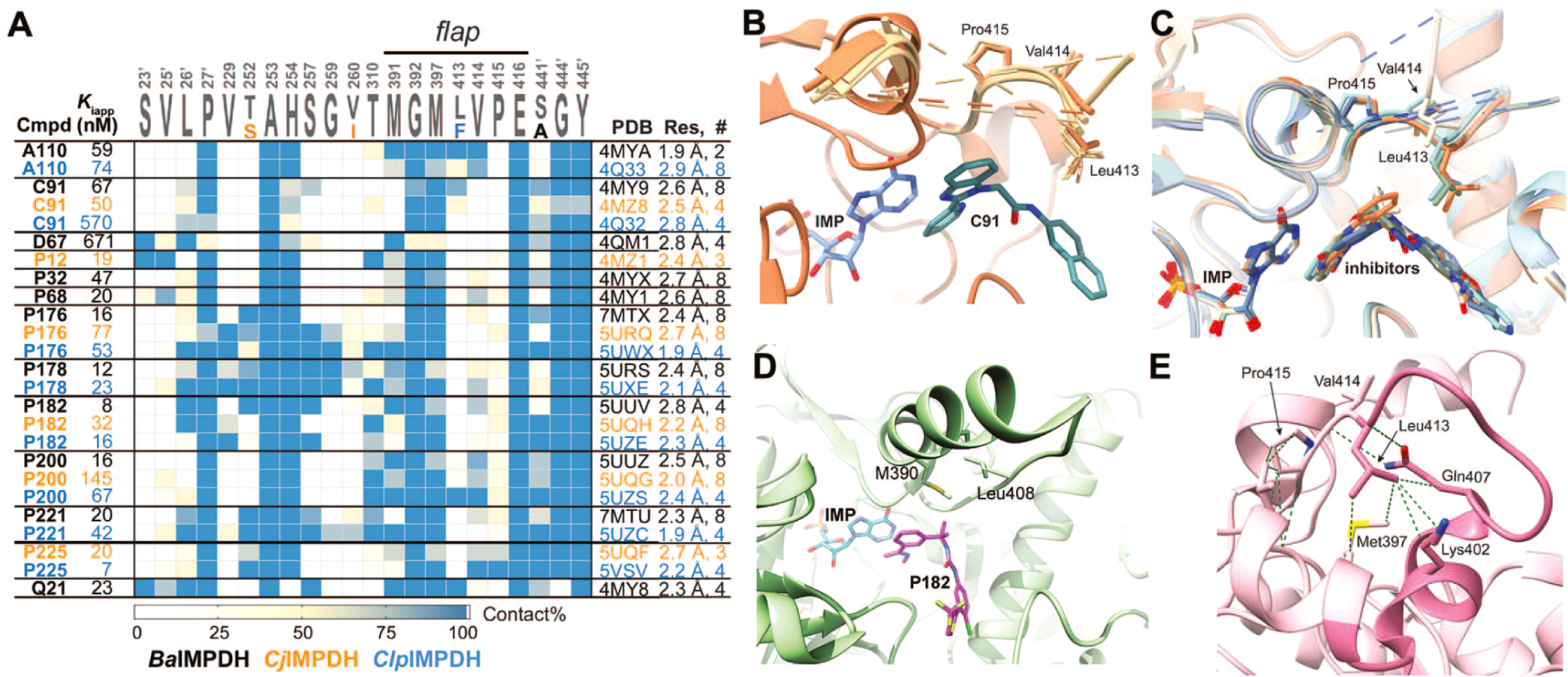
Analysis of crystal structures of bacterial IMPDHs. (A) Heat map of contacts between inhibitors and *Ba-* (black), *Cj-* (orange), and *Clp*IMPDHs (blue). The top list of residues making contacts with inhibitors (*Ba*IMPDH numbering). When a residue is not conserved, it is colored to indicate which enzyme contains the divergent substitution. The inhibitors and values of *K*_i,app_ are listed on the left. The PDB identifier and total number of subunits in the corresponding structure are listed on the right. Contact is defined as van der Waals overlap of −0.4 Å (the default setting in UCSF Chimera). (B) Crystal structure of *Ba*IMPDH·IMP·**C91** (PDB: 4MY9). Four subunits (A, D, E, and F) out of 8 in the crystal structure are overlaid. The structured segments adopt different conformations at different active sites. Deep orange: chain F; light orange: chain A, D, E; **C91**: cadet blue; IMP: cornflower blue. Dashed lines depict residues that are not visible in the crystal structure. (C) Overlay of *Ba*IMPDH with different inhibitors bound. The structured segments adopt different conformations in different inhibitor complexes. Green: **A110** (PDB: 4MYA); orange: **C91** (PDB: 4MY9); deep blue: **D67** (PDB: 4QM1); beige: **P32** (PDB: 4MYX); cyan: **Q21** (PDB: 4MY8). Dashed lines depict residues that are not structured in the crystal structure. (D) Ordered flap in the structure of the complex of *Cj*IMPDH with **P182** (PDB: 5UQH). Met390 and Leu408 correspond to Met397 and Leu413 in *Ba*IMPDH. (E) Flap in the closed conformation in *Ba*IMPDH (PDB: 3TSB). The unstructured region in inhibitor crystal structures is colored deep pink. The contacts Leu413, Val414, and Pro415 made with other residues are labeled as green-dashed lines. Panels (B–E) rendered using UCSF Chimera.^18^

To further assess the contribution of the flap and plasticity of the inhibitor binding site, we determined how many times a particular contact was observed in the active sites of 25 crystal structures of *Ba-*, *Cj-*, and *Clp*IMPDH (Figure 3A). These structures include 5 inhibitor scaffolds. For example, the 8 active sites of *Cj*IMPDH·IMP·**P176** (PDB: 5URQ) had very similar structures, with overall RMSDs ranging from 0.28 to 0.35 Å (Figure 2A). **P176** contacted Val229, Ala253, His254, Ser257, Gly392, Met397, Glu416, Gly444′, and Tyr445′ in all 8 active sites (100% contact frequency). However, the sugar substituent formed different hydrogen bonds in individual active sites, resulting in lower contact frequencies for Gly259 (75%) and Ile260 (38%). We plotted contact frequency in a heat map to visualize regions where the inhibitor interactions varied both within a given structure and between different enzymes (Figure 3A). Ala253 and Glu416 contacted the inhibitor in every active site, as expected. Tyr445′ contacted the inhibitor in all but one active site (139/140); a contact was observed at this site when the criterion was relaxed to a van der Waals overlap of −0.5 Å from the default value of −0.4 Å. These observations further confirm the importance of these interactions.

We focused on the flap because these contacts varied widely in the **P** inhibitors described above and because these interactions involved linker regions that are distinct in different scaffolds. Residues 413–415 were observed in some inhibitor complexes but did not always contact the inhibitor (Figure 3B–D). As in the **P** compounds, these interactions involved the main chains of Val414 and Pro415 and the side chain of Leu413. When the electron density was missing for these residues, the structured segments adopted distinct conformations in individual active sites of a given inhibitor structure as well as in different enzyme–inhibitor complexes (Figure 3B–D). Electron density was not observed for flap residues 400–412 in 139/140 active sites surveyed in the 25 crystal structures. The entire flap was structured in 1 of 8 active sites in the *Cj*IMPDH·IMP·**P182** structure, where it formed an α helix but did not contact the inhibitor (Figure 3D). These observations further indicate that the flap is highly dynamic and may contribute to the inhibitor affinity and selectivity.

### Inhibitor Binding Epitopes Vary in Different Bacterial IMPDHs

**Figure 4:**
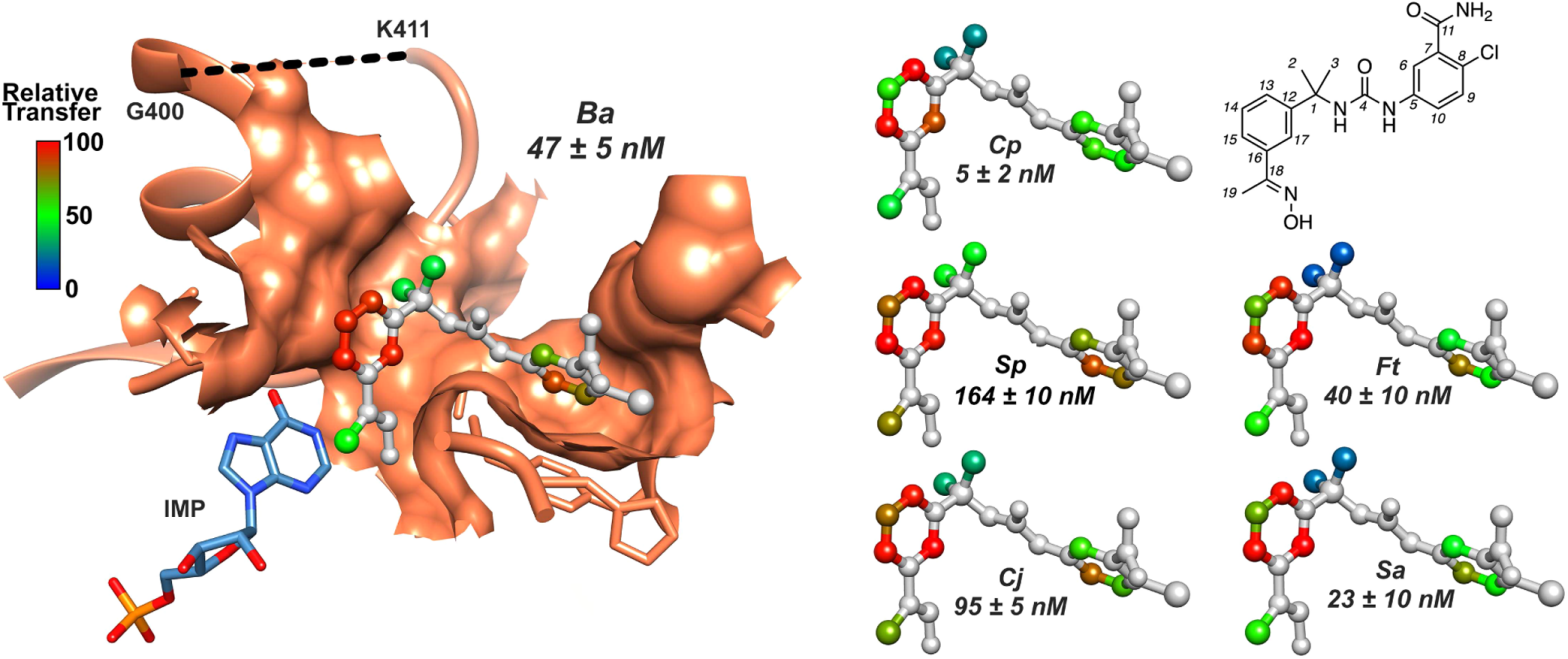
Inhibitor binding epitopes measured by STD-NMR. The E·IMP·**P32** complex of *Ba*IMPDH (PDB: 4MYX) is shown.^6^ Surface shows where the protein (coral) contacts the inhibitor. **P32** is colored by relative STD signals to the respective protons in the E·IMP complexes of 6 bacterial IMPDHs. The values of *K*_i,app_ are shown. Figure rendered using UCSF Chimera.^18^

We turned to saturation transfer difference ^1^H NMR (STD-NMR) experiments to probe inhibitor interactions with bacterial IMPDHs.^19^ This method measures the transfer of magnetization from protein to ligand via intermolecular nuclear overhauser effects (NOEs). The closer a ligand hydrogen is to the protein, the more efficient magnetization transfer and the stronger the corresponding STD-NMR signal (note that only nonexchangeable protons are observed). We chose to examine **P32** because this compound was sufficiently soluble and displayed varying SAR among 6 bacterial IMPDHs, with values of *K*_i,app_ ranging from 5 to 165 nM (Figure 4). **P32** contacts 15 residues in *Ba*IMPDH, of which 3 vary in the 6 enzymes (Figure 3A). Ala441 is often substituted with Ser, but this substitution is found in enzymes with both high (*Cp*IMPDH) and low (*Sp*IMPDH) affinity for **P32**. Substitutions are also observed at Thr252 (Ser in *Cp-* and *Cj*IMPDH) and Leu413 (Met in *Cp*IMPDH and Phe in *Ft-* and *Cj*IMPDH), but in both cases, contacts are observed in only 2 of the 8 active sites in the *Ba*IMPDH crystal structure (PDB: 4MYX).^6^ Therefore, the structural basis of **P32** selectivity is uncertain.

The binding epitope was mapped onto the structure of **P32** by assigning 100% to the proton with the largest STD signal and scaling the other signals accordingly (Figure 4 and Table S4). The binding epitope of **P32** in *Ba*IMPDH qualitatively matched expectations of the crystal structure. The lowest STD signals were observed for the C19 methyl group and the C6 proton; these protons had few protein contacts in the crystal structures. The linker methyl groups cannot be resolved, so the signal reflects both the close protein contacts of the C2 methyl and the solvent-exposed C3 methyl. The L-ring displayed larger STD signals, thus closer contacts, than the R-ring, as expected, given the few protein contacts observed in the crystal structure.

Different binding epitopes were observed among the 6 IMPDHs. Interestingly, no correlation was observed between the values of *K*_i,app_ and the binding epitopes (for example, compare *Ba-*, *Cp-*, and *Ft*IMPDH). The most variation was observed at the C2/3 methyl groups and the L-ring. These atoms interact with the mobile regions of the active site flap, which become ordered when an inhibitor binds.

We also determined the binding epitopes for **P131** where the values of *K*_i,app_ range from 24 to 470 nM among the 6 bacterial IMPDHs (Figure S3 and Table S5). Again, different epitopes were observed among the enzymes that did not correlate with the values of *K*_i,app_. In this case, the R-ring displayed closer contacts than the L-ring likely because the alkylamine group on the oxime disrupts part of the active site.^20^ As with **P32**, the C2/3 methyl groups displayed weak STD signals that ranged from 15 to 59%, indicating that the flap makes transient interactions that vary in different enzymes.

### Residue 413 Is a Determinant of Inhibitor SAR

To further probe the role of the flap in inhibitor SAR, we assessed the effects of substituting Leu413 with Val, Phe, and Ala. These substitutions had little effect on the kinetic parameters with the exception of the *K*_m_ for NAD^+^, which increased by factors of 5.6, 3.5, and 1.4 for L413 V, L413F, and L413A, respectively (Tables 1, S6 and Figures S4–S7). Unfortunately, there are no structures of *Ba*IMPDH with NAD^+^; the analogous residue does not contact the cofactor in IMPDHs from other bacteria (PDB: 4QNE, 4 × 3Z and 4ZQM). Importantly, *K*_m_ is a complex rate constant that includes chemical transformations and product dissociation in addition to NAD^+^ binding, making it difficult to rationalize the effects of mutation on this parameter.

**Table 1:** Steady-State Kinetic Parameters of the *Ba*IMPDH Variants

parameter	WT	L413 V	L413F	L413A
*k*_cat_, s^–1^	2.8 ± 0.2	3.7 ± 0.5	2.6 ± 0.2	1.6 ± 0.1
*K*_m_(*IMP*), μM	59 ± 2	85 ± 5	70 ± 2	69 ± 4
*K*_m_(NAD^+^), μM	590 ± 100	3300 ± 600	2100 ± 200	830 ± 60
*K*_i_(NAD^+^), μM	9600 ± 2100	4400 ± 900	3800 ± 500	8800 ± 900

**Figure 5:**
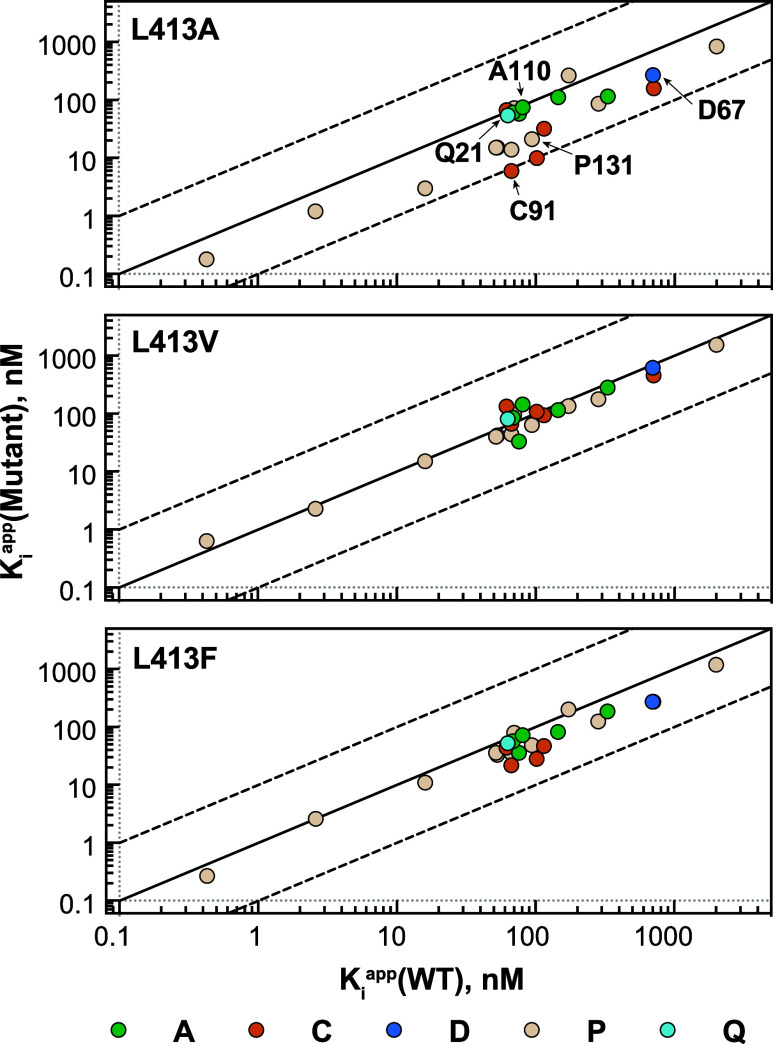
The effects of mutation of Leu413 in *Ba*IMPDH on inhibitor potency. The values of *K*_i,app_ for 23 compounds from 5 different scaffolds **A**, **C**, **D**, **P** and **Q** were determined for both wild-type and mutant enzymes. See Figure S4 for inhibitor structures, and Table S9 for *K*_i,app_ values.

Drawing on our extensive library of bacterial IMPDH inhibitors, we chose 23 representative compounds that span a wide range of potencies and also a variety of scaffolds (Figures S5–S8, Tables S8 and S9). Structures are available for *Ba*IMPDH complexes of 5 inhibitors in this set: **A110**, **C91**, **D67**, **P32**, and **Q21**. The Leu413Val mutation had very modest effects on inhibitor potency, with the ratios of *K*_i,app_ (wild-type) to *K*_i,app_ (L413A) ranging from 0.6 to 2 with an average ratio of 1.2. The inhibitors had more varied responses to the Leu413Phe mutation with *K*_i,app_ ratios ranging from 0.8 to 4 (Figure 5). Therefore, residue 413, and by extension the active site flap, can be a structural determinant of inhibitor potency.

Curiously, only one inhibitor lost potency in L413A as might be expected when a protein contact is lost (Figure 5). Leu413 displayed high contact frequencies (>75%) in crystal structures of **A110** and **Q21** (Figure 3A), but the values of *K*_i,app_ were the same for wild-type and L413A (note that **A110** rearranged in the X-ray beam during data collection, which may have influenced binding^6^). High contact frequency was also observed for **C91**, but its affinity increased by a factor of 10 in L413A. The affinity of **C85**, a close analog of **C91**, also increased 10-fold. Affinity increased for the majority of the inhibitors (15 out of 23), indicating that Leu413 impedes the binding of most inhibitors.

### Open Flap Conformation Predominates in *Ba*IMPDH

Competition between the flap and inhibitors for the cofactor site complicates the interpretation of mutagenesis experiments, since a substitution could potentially change the equilibrium between open and closed conformations (defined by *K*_c_) as well as interactions with inhibitors.^1^ The closed conformation is observed in the crystal structure of the *Ba*IMPDH·P_i_ complex (PDB: 3TSB(6)) (Figure 3E). Leu413 is buried so that its side chain forms a hydrophobic core with Met397, Lys402, and Gln407. The Leu413Val substitution is expected to destabilize the closed conformation, increasing the fraction of enzyme in the open conformation and thus potentially increasing the potency of all inhibitors equivalently. Since the Val substitution has little effect on inhibitor potency, then the open conformation must be favored in *Ba*IMPDH. Assuming that the Val substitution destabilizes the closed conformation by 1.71 kcal/mol (calculated by SAAFEC-SEQ^21^), then the value of *K*_c_ for the wild-type enzyme must be less than or equal to 0.2 (Figure S9).

We used a multiple inhibitor experiment to corroborate this conclusion.^22^ In brief, if the closed conformation predominates, a synergistic interaction will be observed between the nicotinamide riboside analog tiazofurin and ADP: one inhibitor, e.g., ADP, will induce the open conformation, allowing the other, e.g., tiazofurin, to bind (note that the enzyme used in our experiments does not contain the regulatory CBS domains that can also bind ADP). However, the presence of ADP (4 mM) did not increase the potency of tiazofurin (*K*_i,app_ = 0.62 versus 0.55 mM in the absence and presence of ADP, respectively; Table S10). These experiments further suggest that the open conformation predominates.

### Transient Kinetics of *Ba*IMPDHs Inhibition

*K*_i,app_ values provide only general information on inhibitor binding and do not reveal the affinity of the inhibitor for E·IMP and E-XMP*. Therefore, we decided to subject representative inhibitors from each series (**A110**, **C91**, **D67**, **P131**, and **Q21**) to more detailed kinetic investigations.

The transient kinetics of both wild-type and L413A interacting with mutation-insensitive inhibitor **A110** were investigated by stopped-flow experiments. The enzyme was preincubated with saturating IMP (1 mM) to ensure that the reaction starts from E·IMP. As a result, the free enzyme is essentially absent in the stopped-flow assay, which simplifies analysis. The reaction was initiated by the addition of varying concentrations of NAD^+^ (0.25–8 mM). NADH (60 μM) or **A110** (6 μM) were optionally added to probe product inhibition by NADH and inhibition by **A110**, respectively.

**Figure 6:**
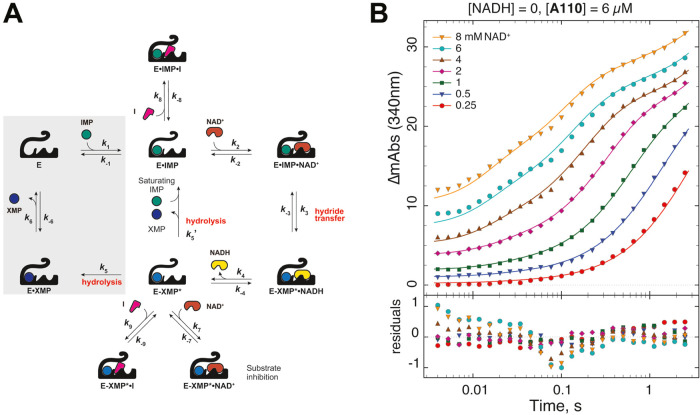
Kinetic analysis of inhibitor binding. (A) Kinetic models for data analysis. A minimal kinetic model shown in the white area was used to analyze the stopped-flow data. The alternate path in the gray area was added to the model to analyze initial rate data. (B) Representative set of transient kinetic traces. L413A (2.3 μM), **A110** (6 μM), and [IMP] = 1 mM.

Multiple combinatorial replicates were analyzed and fit to the model shown in Figure 6A (Figures S10–S12 and Tables S11–S16^17^). Representative progress curves and fits are shown in Figure 6B and the final results are given in Table 2. For both wild-type and L413A, only the lower limits could be determined for NADH release and association rate constants, *k*_4_ and *k*_–4_, according to the empirical Δ*S*SQ method.^23^ Other rate constants were well determined with geometric standard deviation (GSD) < 1.5. The best-fit values of all microscopic rate constants for the wild-type interaction with **A110**, listed in Table 2, are in very good agreement with previously published results.^17^

**Table 2:** Microscopic Rate Constants from Combinatorial Replicates Determined by the Global Fit of Stopped-Flow Transient Kinetic Data^a^

	WT		L413A	
parameters	GM^b^	GSD	GM^c^	GSD
*k*_2_, μM^–1^ s^–1^	0.0309	1.04	0.00852	1.03
*k*_–2_, s^–1^	11.7	1.44	6.9	1.25
*k*_3_, s^–1^	140	1.07	149	1.07
*k*_–3_, s^–1^	59.2	1.16	173	1.37
*k*_4_^d^, s^–1^	>446	1.06	>255	1.15
*k*_–4_^d^, μM^–1^ s^–1^	>3.8	1.06	>0.67	1.28
*k*_5_^′^, s^–1^	22.9	1	6.21	1.03
*k*_7_, μM^–1^ s^–1^	0.00398	1.16	0.000917	1.11
*k*_–7_, s^–1^	31.8	1.16	4.12	1.11
*k*_8_, μM^–1^ s^–1^	9.33	1.04	10.9	1.11
*k*_–8_, s^–1^	15.6	1.04	27.5	1.06
*k*_9_, μM^–1^ s^–1^	3.52	1.02	2.83	1.07
*k*_–9_, s^–1^	0.3	1.02	0.22	1.09
*k*_–2_/*k*_2_, μM	380		810	
*k*_3_/*k*_–3_, −	2.4		0.86	
*k*_4_/*k*_–4_, μM	120		370	
*k*_–7_/*k*_7_, μM	8000		4500	
*k*_–8_/*k*_8_, μM	1.7		2.5	
*k*_–9_/*k*_9_, μM	0.085		0.078	

a GM, geometric mean; GSD, geometric standard deviations.

b Geometric mean from 12 independent best-fit values.

c Geometric mean from 18 independent best-fit values.

d Lower limit (ΔSSQ = 5%^23^); upper limit was not defined by the data.

The Leu413Ala mutation changes all of the microscopic rate constants that characterize the catalytic cycle. The association and dissociation rate constants for the binding of NAD^+^ and NADH are lower than those of the wild-type by factors of 1.7–8.5. Although the change in microscopic rate constant values varied, the corresponding equilibrium constants remain invariant within a factor of approximately three. For example, while *k*_7_ and *k*_–7_ changed by a factor of 4.3 and 7.7, the equilibrium constant *k*_–7_/*k*_7_ shifted by only approximately 40%. The equilibrium constant for the hydride transfer step is about 2.7 times lower in the mutant, and the composite hydrolysis step *k*_5_^′^ is slower by a factor of 3.7. Despite these changes, there is no significant variation in the accumulation of E·IMP and E-XMP* in the catalytic cycles of the wild-type and L413A (Figure S12). These observations were unexpected because Leu413 was not known to interact with substrate/product or cofactor and previous mutations of flap residues specifically perturbed the hydrolysis step.^24^ Several conformational changes occur during the IMPDH catalytic cycle, for example, when substrate/product, cofactor or K^+^ bind, E-XMP* forms and NADH departs.^24^ The effects of the mutation on the microscopic rate constants but not the equilibria suggest that Leu413 may be involved in the transition states for these conformational changes. Mutations that change the rate of protein folding but not protein stability have been observed in other systems.^25^

In contrast, the mutation had no effect on the microscopic rate constants *k*_8_, *k*_–8_, *k*_9_, and *k*_–9_, which characterize the binding of **A110** to E·IMP and E-XMP*. **A110** binds preferentially to E-XMP* in both wild-type and mutant by factors of 20–30. These results signify that the substitution of Leu413 has no effect on the affinity of **A110**, which is surprising since Leu413 contacts **A110** in both active sites in the crystal structure (Figure 3A).

### Determination of Substrate-Related Rate Constants

To interrogate the substrate complexes, we performed initial rate experiments varying IMP, NAD^+^, and XMP concentrations at subsaturating concentrations. These experiments enabled the independent determination of the microscopic rate constants *k*_1_, *k*_–1_, and *k*_5_, as well as the XMP dissociation equilibrium constant *k*_6_/*k*_–6_, appearing in the kinetic model (Figure 6A). These important kinetic constants are absent in the transient kinetic model.

**Figure 7:**
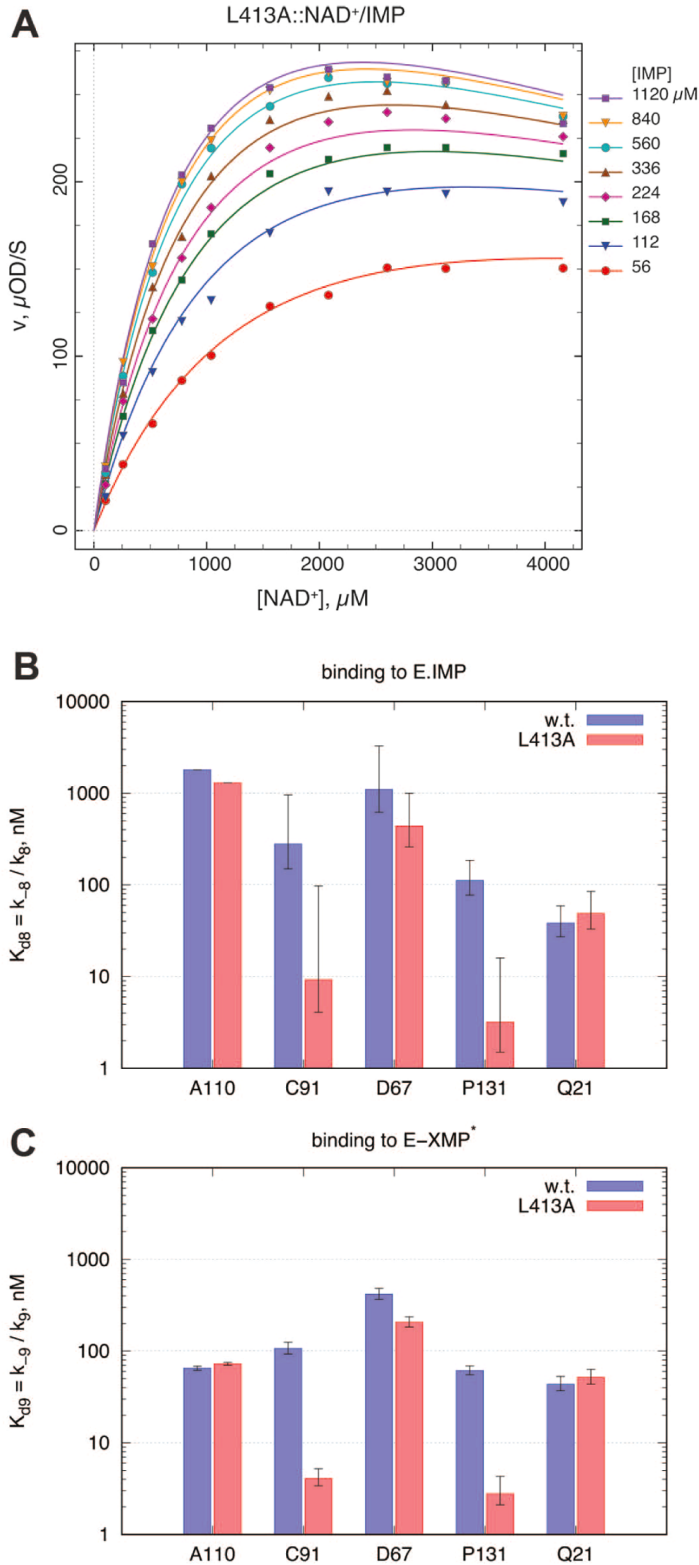
Determination of inhibitor binding to E·IMP and E-XMP*. (A) A representative subset of the multidimensional global of initial rate data involving the L413A mutant. NAD^+^ concentration is shown on the horizontal axis; the IMP concentration (μM) is shown in the legend on the right. (B, C) The best-fit values are plotted as a bar and the 90% confidence limits are shown as error bars. Numerical values of kinetic parameters are shown in the SI (Table S9). For further details, see the text.

A representative subset of the multidimensional global data set for L413A is shown in Figure 7. The full details, including the raw experimental data and the precise definition of the generalized numerical fitting model,^26^ are presented in the SI (Section 10). Importantly, all microscopic rate constants appearing in the transient kinetic model in Figure 6A except *k*_5_^′^ were held at fixed values determined in the stopped-flow experiments. The bimolecular association rate constant for XMP, *k*_–6_, was held fixed at the diffusion controlled value of 10^6^ M^–1^ s^–1^;^27^ the optimized parameters were the rate constants *k*_1_, *k*_–1_, *k*_5_, and *k*_6_.

As observed for NAD^+^ binding, the association and dissociation rate constants for IMP binding in L413A are slightly lower than those in wild-type, by a factor of approximately 2 (Table 3). However, the equilibrium constant *K*_*d*1_ = *k*_–1_/*k*_1_ only shifts 20% with overlapping 90% confidence intervals (wild-type, 116–209 μM versus mutant 90–180 μM). Similarly, the confidence intervals for the XMP dissociation equilibrium constant *K*_*d*6_ = *k*_6_/*k*_–6_ also overlap (359–456 μM for wild-type versus 386–437 μM for L413A). These results signify that IMP association and XMP dissociation remain essentially unchanged with the mutation. As for the hydrolysis step, the value of *k*_5_ decreases by a factor of 2. Importantly, the values of *k*_5_ are similar to those of the composite rate constant *k*_5_^′^ determined in the presteady-state experiments, indicating that hydrolysis is the rate-limiting step.

**Table 3:** Substrate-Kinetic Properties of IMPDH Variants Determined from Initial Rate Data to the Mechanism in Figure 6^a^

parameter	WT	L413A
*k*_1_, μM^–1^ s^–1^	0.279 [0.256, 0.306]	0.178 [0.158, 0.203]
*k*_–1_, s^–1^	43.7 [35.6, 53.6]	22.5 [18.2, 28.4]
*k*_5_, s^–1^	19.1 [17.3, 21.1]	9.03 [8.52, 9.56]
*k*_6_/*k*_–6_, μM	404 [359, 456]	411 [386, 437]
*k*_–1_/*k*_1_, μM	157 [116, 209]	126 [90, 180]

a The comma-separated values in square brackets are lower and upper limits, respectively, of the 90% confidence level intervals determined by the profile-*t* search method of Bates and Watts.^28−30^ For details, see the text.

### L413A Has Similar Effects on Inhibitor Affinity to E·IMP and E-XMP*

Five compounds from different series (**A110**, **C91**, **D67**, **P131**, and **Q21**) were subjected to a detailed inhibition kinetic study. Steady-state initial rate experiments were performed with simultaneous variation of three reaction components, namely, IMP, NAD^+^, and the inhibitor of interest. Each four-dimensional data set (three independent variables plus the reaction rate as the dependent variable) were fit globally^31^ to a generalized numerical model^26^ represented schematically in Figures 6A and S13. In the requisite system of simultaneous nonlinear algebraic equations, all microscopic rate constants except *k*_–8_ and *k*_–9_ were held fixed at values determined either in the relevant stopped-flow experiments (*k*_2_ through *k*_–7_ except *k*_5_^′^ and *k*_6_), or in the corresponding substrate-kinetic initial rate experiment (*k*_1_, *k*_–1_, *k*_5_ and *k*_6_). The bimolecular association rate constants *k*_8_ and *k*_9_ were held fixed at 10^6^ M^–1^ s^–1^. The best-fit values are summarized in Figure 7 and Table 4 with the exception of the values *K*_*d*8_ = *k*_–8_/*k*_8_ for **A110**, which are based on the lower limits at the 95% probability level. The 95% confidence intervals for all enzyme versus inhibitor combinations are listed in SI Section 9.4. The full details of this analysis are described in SI Section 10.

**Table 4:** Inhibitor Dissociation Constants from E·IMP and E-XMP* Determined by Steady-State Kinetics^a^

inhibitor	parameters	WT	L413A
**A110**	*k*_–8_/*k*_8_, nM	na	na
	*k*_–9_/*k*_9_, nM	65 [62, 68]	73 [70, 75]
**C91**	*k*_–8_/*k*_8_, nM	280 [150, 960]	9.3 [4.1, 97]
	*k*_–9_/*k*_9_, nM	110 [93, 130]	4.1 [3.4, 5.2]
**D67**	*k*_–8_/*k*_8_, nM	1100 [620, 3300]	440 [260, 1000]
	*k*_–9_/*k*_9_, nM	420 [370, 480]	210 [180, 240]
**P131**	*k*_–8_/*k*_8_, nM	110 [77, 190]	3.2 [1.5, 16]
	*k*_–9_/*k*_9_, nM	61 [55, 69]	2.8 [2.1, 4.3]
**Q21**	*k*_–8_/*k*_8_, nM	38 [27, 59]	49 [33, 85]
	*k*_–9_/*k*_9_, nM	43 [37, 53]	52 [43, 63]

a The comma-separated values in square brackets are lower and upper limits, respectively, of the 90% confidence level intervals determined by the profile-*t* search method of Bates and Watts.^28−29,30^ na, not applicable.

All tested inhibitors bind to both E·IMP and E-XMP* in wild-type and L413A. **A110** displays a 20-fold preference for E-XMP* in wild-type, in good agreement with the transient kinetics experiments (Table 2). **C91**, **D67**, and **P131** have only a 2-fold preference. Only **Q21** has the same affinity for both enzyme forms. The inhibitors′ responses to the mutation fall into three categories. Two inhibitors, **A110** and **Q21**, are entirely insensitive to the mutation. **D67** showed a modest 2-fold change in both *K*_*d*8_ and *K*_*d*9_. Lastly, **C91** and **P131** displayed an order of magnitude shift in the binding affinity for both E·IMP and E-XMP*. Importantly, the parallel changes to both *K*_*d*8_ and *K*_*d*9_ suggest that Leu413 plays a role similar to that of E·IMP and E-XMP*. Therefore the structures of the E·IMP complexes are useful models of the E-XMP* complexes.

## Conclusions

The experiments described above were designed to identify the structural determinants of the SAR of the bacterial IMPDH inhibitors. We considered two alternatives that would not be captured in the available crystal structures of the E·IMP complexes. First, the varying SAR originates in interactions with the covalent intermediate E-XMP*. Second, the varying SAR derives from interactions with the mobile active site flap that are not observed in the crystal structures. STD NMR experiments show that binding epitopes vary among bacterial IMPDHs despite the close similarity of the inhibitor binding sites and reveal that the largest differences are found in the portion of the inhibitor that interacts with the flap. Our kinetic characterization indicates that the substitution of Leu413 with Ala has equivalent effects on inhibitor affinity to both E·IMP and E-XMP*, which suggests that Leu413, and by extension the flap, plays a similar role in both complexes. However, the consequences of this substitution could not be readily predicted from the crystal structures. The Leu413Ala substitution increased the affinity of most inhibitors, even those that contacted this residue. These results demonstrate that protein-inhibitor contacts do not necessarily indicate favorable interactions, and poorly structured mobile regions should not be ignored when assessing binding determinants.

## Methods

Methods are included in the Supporting Information (Section 1).

## Supporting Information Available

Methods, additional figures and tables, mathematical models, nonlinear regression procedures and raw experimental data (PDF)

## Abbreviations

*Ba*: *Bacillus anthracis*
*Cj*: *Campylobacter jejuni*
*Clp*: *Clostridium perfringens*
*Cp*: *Cryptosporidium parvum*
*Sp*: *Streptococcus pyogenes*
*Sa*: *Staphylococcus aureus*
*Mtb*: *Mycobacterium tuberculosis*
*Bb*: *Borrelia burgdorferi*
DMSO: dimethyl sulfoxide
DTT: dithiothreitol
EDTA: ethylenediaminetetraacetic acid
GM: geometric mean
GSD: geometric standard deviation
IMPDH: inosine 5′-monophosphate dehydrogenase
IMP: inosine 5′-monophosphate
XMP: xanthosine 5′-monophosphate
SAR: structure–activity relationship
SI: Supporting Information document
