# Supplementary material for: Role of the Mobile Active Site Flap in IMP Dehydrogenase Inhibitor Binding
Source: ACS Infect Dis. 2025 Jan 29;11(2):442–52. doi: 10.1021/acsinfecdis.4c00636 (PMC11841048; doi:10.1021/acsinfecdis.4c00636)
Supplement: Supplementary file 1 — id4c00636_si_001.pdf [file id4c00636_si_001.pdf]

Supporting Information: The Role of the Mobile Active Site Flap in IMP Dehydrogenase Inhibitor Binding

Xingyou Wang,[†,@] Masha M. Rosenberg,[‡,△] Youngchang Kim,[¶,§] Natalia Maltseva,[¶,§] Gregory D. Cuny,[‖] Andrzej Joachimiak,[¶,§,⊥] Petr Kuzmič,[*,#] and Lizbeth Hedstrom[*,‡,†]

†*Department of Chemistry, Brandeis University, Waltham, Massachusetts, 02454, USA*

‡*Department of Biology, Brandeis University, Waltham, Massachusetts, 02454, USA*

¶*Center for Structural Biology of Infectious Diseases, Consortium for Advanced Science and Engineering, University of Chicago, Chicago, Illinois 60667, USA*

§*The Structural Biology Center, X-ray Science Division, Argonne National Laboratory, Lemont, Illinois 60439, USA*

‖*Department of Pharmacological and Pharmaceutical Sciences, College of Pharmacy, University of Houston, Houston, Texas 77204, USA*

⊥*Department of Biochemistry and Molecular Biology, University of Chicago, Chicago, Illinois 60367, USA*

#*BioKin Ltd., Watertown, Massachusetts, 02472, USA*

@*Present address: Lifemine Therapeutics, Cambridge, Massachusetts, 02140, USA*

△*Present address: Believer Meats, Moti Kind St 10, Rehovot 7638519 Israel*

E-mail: petr.kuzmic@gmail.com; hedstrom@brandeis.edu

Contents

1 Methods **S-5**

1.1 Protein purification and crystallography S-5

1.1.1 Protein production and crystallization S-5

1.1.2 Data Collection, structure solution and refinement S-6

1.2 Crystallographic structure analysis S-8

1.3 Determination of the apparent inhibition constant $K_{\mathrm{i,app}}$ S-8

1.4 Stopped-flow Experiments S-9

1.5 Determination of initial rates S-9

1.6 Data Analysis S-10

1.6.1 Analysis of transient kinetics data S-10

1.6.2 Analysis of initial rate data S-11

1.6.3 STD-NMR experiments S-11

2 Sequence alignment **S-12**

3 X-Ray crystal structures **S-13**

3.1 Ten newly described crystal structures in this study S-13

3.2 List of 25 analyzed crystal structures S-16

4 STD-NMR experiments **S-18**

5 Substrate kinetic properties of IMPDH mutants **S-20**

5.1 Theory S-20

5.2 Determination of apparent Michaelis constants S-21

5.2.1 Representative example S-21

5.2.2 Summary of results S-22

6 Effect of Leu413 mutations on inhibition **S-24**

6.1 Inhibitor structures S-24
6.2 Reagent concentrations S-25
6.3 Determination of initial reaction rates S-25
6.4 Analysis of initial reaction rates S-27
6.5 Sensitivity of $K_{\mathrm{i,app}}$ to mutations S-29

7 Simulation of effects of mutations on equilibrium between [E-XMP*]$_{\mathrm{open}}$ and [E-XMP*]$_{\mathrm{closed}}$ S-31

8 Multiple inhibitor experiment S-33

9 Stopped-flow transient kinetics S-34

9.1 Experimental data S-34
9.1.1 Data reduction S-34
9.1.2 Combinatorial replication S-34
9.2 Theoretical model S-35
9.2.1 Postulated stopped-flow kinetic mechanism S-35
9.2.2 Regression equation and the associated ODE system S-36
9.2.3 Global vs. local classification of model parameters S-38
9.2.4 Empirical confidence intervals S-39
9.3 Representative example S-39
9.3.1 Typical experimental data for the L413A mutant S-39
9.3.2 DynaFit script file listing S-43
9.3.3 Results of fit S-45
9.4 Summary of results S-47
9.4.1 Individual combinatorial replicates S-47
9.4.2 Geometric means and geometric standard deviations S-48

10 Steady-state initial rate kinetics S-50

10.1 Experimental data . . . S-50
10.2 Theoretical model . . . S-50
10.2.1 Postulated stopped-flow kinetic mechanism . . . S-50
10.2.2 Mathematical models . . . S-50
10.2.2.1 Model "S": Substrate catalysis . . . S-51
10.2.2.2 Model "A": Inhibitor binding to a single enzyme form (EQ) S-53
10.2.2.3 Model "B": Inhibitor binding simultaneously to two enzyme forms (EA, EQ) . . . S-55
10.2.2.4 Model "C": Inhibitor binding simultaneously to three enzyme forms (E, EA, EQ) . . . S-57
10.2.3 Stepwise regression procedure . . . S-60
10.2.4 Model selection method . . . S-61
10.3 Representative examples . . . S-61
10.3.1 Substrate kinetics of wild-type IMPDH . . . S-61
10.3.2 Kinetics of wild-type IMPDH inhibited by **C91** . . . S-66
Intermediate results . . . S-73
Kinetic constants . . . S-73
Information-Theoretic Criteria – Full Set . . . S-73
Final results . . . S-74
Information-Theoretic Criteria – Reduced Set . . . S-74
Model Discrimination Analysis – Conclusions . . . S-74
Acceptable parameters . . . S-74

References **S-76**

1 Methods

1.1 Protein purification and crystallography

1.1.1 Protein production and crystallization

The plasmid expressing His_6-tagged *Ba*IMPDH lacking the CBS subdomain (previously designated *Ba*IMPDHΔL) was described in previous ref.[S1] Quikchange PCR was performed to incorporate the L413A, L413V, and L413F point mutations. The plasmids of wild-type and mutant *Ba*IMPDH were transformed into *E. coli* BL21(Δ*guaB*) competent cells. Cells were grown in LB media at 37°C to OD_{600} 0.6–0.8 and then induced with 0.5 mM IPTG at 30°C for 20 hr. The overexpressed His_6-tagged *Ba*IMPDHs were purified by Ni-NTA column at 4°C in lysis buffer [50 mM phosphate buffer (pH 8.0), 500 mM KCl, 10 mM imidazole, 1 mM DTT, and 5% glycerol]. The enzymes were eluted with 250 mM imidazole in lysis buffer and were dialyzed in 4 L dialysis buffer [50 mM Tris-HCl (pH 8.0), 100 mM KCl, 3 mM EDTA, 1 mM DTT, 5% glycerol] with three buffer changes. The concentrations of purified wild-type and mutant *Ba*IMPDHs were determined by the Bradford assay using IgG as the standard and divided by a factor of 2.6. The purified proteins were stored at -80°C.

*Cj*IMPDHΔS (CBS domain V92-T195 deleted and replaced with G)[S1] and the ΔL mutant *Clp*IMPDHΔL (CBS domain Q89-R215 deleted and replaced with SGG)[S1] were expressed and purified according to the Center for Structural Genomics of Infectious Diseases (CSGID) standard protocol.[S2]

The proteins contained an N-terminal His6-tag plus Tobacco Etching Virus (TEV) protease cut site. Briefly, a culture of enriched M9 medium was grown at 37 °C with a 180 rpm shaking. At OD_{600} 0.9, the culture was cooled down to 4 °C for a half hour and supplied with 0.5 mM (final) isopropyl β-D-1-thiogalactopyranoside (IPTG). Protein expression was induced overnight at 18 °C. After a centrifugation, the cell pellet was resuspended in a lysis buffer containing 500 mM KCl, 5 % (v/v) glycerol, 50 mM HEPES, pH 8.0, 20 mM imidazole and 10 mM β-mercaptoethanol and treated with lysozyme (1 mg/ml) and sonica-

tion. The enzymes were purified using nickel(II) affinity chromatography (IMAC) using 5 ml HisTrap HP column (Cytiva, Marlborough, USA) on AKTA express (Cytiva, Marlborough, USA). The buffer solutions used in the purification included KCl in place of NaCl since most IMPDHs require K^+ to be most active. Purified fractions containing the IMPDH were characterized by SDS gel electrophoresis. *Clp*IMPDHΔL was treated with TEV protease to remove the His-tag and dialyzed into crystal buffer (20 mM HEPES, pH 8.0, 150 mM KCl and 1.5 mM TCEP). The His-tag of *Cj*IMPDHΔS was not removed; in this case protein was exchanged into crystal buffer with size exclusion chromatography (SEC). IMPDHs were concentrated to 29.1 mg/ml for *Cj*IMPDHΔS and 23.0 mg/ml for *Clp*IMPDHΔL, and flash-frozen in liquid nitrogen. The final yields for *Cj*IMPDHΔS and *Clp*IMPDHΔL were 4.1 and 4.2 mg/g cell paste , respectively.

Crystallization experiments were performed using the sitting-drop, vapor-diffusion method in 96-well CrystalQuick plates (Greiner Bio-One, Monroe, NC) with the liquid dispenser Mosquito (LabTech, Cambridge, MA). For co-crystallization trials, inhibitors were used at a 2-20 fold molar excess (typically 1.5-5 mM), and IMP was used at 10-fold molar excess (3-5 mM) over protein concentration (0.3-0.5 mM). For each condition, 0.4 μL of protein solution containing protein, IMP and inhibitor and 0.4 μL crystallization formulation were mixed and the mixture was equilibrated against a 135 μL reservoir. The crystal screens of index (Hampton Research, Aliso Viejo, CA), JBScreen Wizard (Jena Bioscience, Jena, Thuringia, Germany) and four MCSG crystallization screens (MCSG1-4, Anatrace Inc, Maumee, OH) were used and conditions yielding diffraction quality crystals typically appeared within 2–7 days. Crystallization conditions for the crystals used for data collections are listed in the Table S2.

1.1.2 Data Collection, structure solution and refinement

All the X-ray diffraction experiments were performed at the Structural Biology Center at the Advanced Photon Source, Argonne National Laboratory.[S3] Prior to flash-cooling in liquid

nitrogen, the crystals were cryoprotected in a solution containing the crystallization mother liquor plus 15% ethylene glycol or 20 % glycerol. All data were collected at 100 K on a Dectris Pilatus3 X 6M detector on 19-ID or ADSC Q210r detector on 19-BM using SBCCOLLECT with an x-ray energy near 12.66 keV. HKL3000 suite27 was used to process the diffraction images and to scale to final data sets for structure determination and refinement.[S4]

All crystal structures reported here were determined by the molecular replacement (MR) method using Molrep[S5] and REFMAC[S6] as a part of HKL3000 suite. The structure of the *Cj*IMPDHΔS•IMP complex (PDBID 4R7J), after removing IMP, was used as a search model to determine all structures of *Cj*IMPDHΔS•IMP•inhibitor complexes by molecular replacement. For the *Clp*IMPDHΔL•IMP•inhibitor complexes, the crystal structure of the *Clp*IMPDHΔL complexed with IMP and the inhibitor **C91** (PDBID 4Q32)[S1] or the crystal structure of the *Clp*IMPDHΔL complexed with IMP and the inhibitor **A110** (PDBID 4Q33),[S1] after IMP and the inhibitor were removed, was used as a search model for molecular replacement. In each of resulting MR structures, the presence of the well-defined extra electron density was apparent in the IMP site as well as the cofactor binding site from the initial electron density map (*Fo*). The subsequent iterative steps of alternating manual adjustments and computational refinements were done using Coot[S7] and Phenix[S8] until the convergence was achieved with an optimal stereochemistry for each structure. To reduce the model bias, all structures were subjected to 1-2 cycles of simulated annealing with the starting temperature of 5000 K at early stages of the refinement. All structures were checked with PROCHECK[S9] and Molprobity[S10] and Ramachandran plot and validated with PDB validation server. The refinement statistics for the converged final models are given in Table S2.

The atomic coordinates and structure factors have been deposited in the Protein Data Bank (PDB) and the accession codes are 5UQF, 5UQH, 5UQG, 5URQ, 5UWX, 5UXE, 5UZE, 5UZC, 5UZS and 5VSV for the structures of *Cj*IMPDHΔS•IMP•**P225**, *Cj*IMPDHΔS•IMP• **P200**, *Cj*IMPDHΔS•IMP•**P182**, *Cj*IMPDHΔS•IMP•**P176**, *Clp*IMPDHΔL•IMP•**P176**,

*Clp*IMPDHΔL•IMP•**P178**, *Clp*IMPDHΔL•IMP•**P182**, *Clp*IMPDHΔL•IMP•**P221**, *Clp*IMPDHΔL•IMP•**P200**, and *Clp*IMPDHΔL•IMP•**P225**, respectively.

1.2 Crystallographic structure analysis

Twenty-five E•IMP•I crystal structures from RCSB Protein Data Bank were analyzed using UCSF Chimera.[S11] The contacts between enzyme and inhibitors were defined as protein atoms within a -0.4 Å overlap with with inhibitor atoms. Contacts were identified in all active sites in the asymmetric unit of each crystal structure. Contacts were further classified as hydrogen bond/dipole (includes halogen bonds), hydrophobic or pi-stacking. The number of active sites in the asymmetric unit varied from 1 to 8 among the crystal structures. The conformation of the inhibitor and protein side chains often varied in different active sites within an asymmetric unit. We determined the percentage of active sites that display each contact as a measure of this heterogeneity. The percentage of contacts were calculated for each residue as in Eqn (S1).

$$Contact\ \% = \frac{number\ of\ subunits\ with\ the\ contact}{total\ number\ of\ subunits} \times 100 \tag{S1}$$

1.3 Determination of the apparent inhibition constant $K_{\mathrm{i,app}}$

The inhibition assays were performed on BioTek Synergy H1 microplate reader using 96-well plates. Inhibitor stocks and dilutions were prepared in DMSO, and substrate stocks were prepared in MilliQ water. The enzymes were preincubated with inhibitor in assay buffer [50 mM Tris-HCl (pH 8.0), 100 mM KCl, 3 mM EDTA, 1 mM DTT] at room temperature for 5 minutes. Reactions were initiated by adding NAD^+ and IMP. The conditions used for each enzymes are listed in Section 6. The production of NADH at 12 varying inhibitor concentrations (0 to 5 μM, 1:2 dilution ratio) was monitored by UV/Vis absorbance at 340 nm for 15 minutes. Initial reaction rates were determined by fitting the four data points collected over the first five minutes to the straight-line model. $K_{\mathrm{i,app}}$ values were

determined by nonlinear least-squares fit of the initial rates, v, to Eqn (S2), where V_0 is the uninhibited reaction rate; [E] and [I] are total or analytic concentrations of the enzyme and inhibitor, respectively; and $K_{\mathrm{i,app}}$ is the apparent inhibition constant. The total enzyme concentration [E] was optionally treated as an adjustable model parameter, according to the method described previously.[S12] For details, see Section 6.

$$v = V_0 \frac{[\mathrm{E}] - [\mathrm{I}] - K_{\mathrm{i,app}} + \sqrt{([\mathrm{E}] - [\mathrm{I}] - K_{\mathrm{i,app}})^2 + 4\,[\mathrm{E}]\,K_{\mathrm{i,app}}}}{2\,[\mathrm{E}]} \tag{S2}$$

1.4 Stopped-flow Experiments

The stopped-flow experiments were performed on an Applied Photophysics SX20 spectrophotometer. Syringe A contained wild-type (8 μM) or L413A (16 μM) enzymes with IMP (1 mM) and DTT (2 mM) in assay buffer (50 mM Tris, pH 8.0, 100 mM KCl, 30 mM EDTA) with 2.5% DMSO. Syringe B contained varying concentrations of NAD^+ (0.5, 1, 2, 4, 8, 12, 16 mM) and IMP (1 mM) in assay buffer with 2.5% DMSO. NADH (120 μM) or **A110** (12 μM) was optionally added to syringe B to probe product inhibition and **A110** inhibition. The solutions in both syringes were 1:1 mixed after preincubation at 25°C for 5 min. The absorbance at 340 nm was recorded to monitor the NADH production. For each progress curve, 10000 time-points were recorded during the initial 2.5 seconds of the reaction. The traces used in the data analysis were the average of 12 recorded progress curves by injections from the same sample preparation. Replicated experiments were repeated on different days starting from fresh stock solutions, to ensure true randomization. Each full kinetic trace containing 10000 time-points was filtered with exponential spacing. Only the resulting 25 sampled time-points were analyzed; see the Section 9 for details.

1.5 Determination of initial rates

The steady-state kinetic experiments were performed by measuring the initial velocities of reactions where the concentrations of NAD^+, IMP, NADH, XMP, and inhibitors (**A110** or

C91) were varied. The experiments were performed either on Shimadzu UV-1800 spectrometer using 1 cm cuvettes or on BioTek Synergy H1 microplate reader using 96-well plates. NADH production was monitored for each reaction by the absorbance at 340 nm. The total reaction time was 5 min. The resulting kinetic trace containing 8 individual time points. The initial reaction rate, in absorbance units per second, was obtained as the slope of the linear least-squares fit of the raw time vs. absorbance data.

1.6 Data Analysis

All data analysis was performed using the software package DynaFit.[S13,S14] Full details, including raw experimental data and the detailed specification of the numerical fitting models, are described in section 9.

1.6.1 Analysis of transient kinetics data

Stopped-flow transient kinetic data was analyzed by using the combinatorial replicate method described previously in ref.[S15] Each global combinatorial replicate was fit to a system of simultaneous first-order ordinary differential equations, which correspond to the kinetic mechanism shown in Figure 6A. The relevant ordinary differential equation system is listed in section 9. Note that the free enzyme E does not appear in the kinetic model because all stopped-flow experiments were performed at saturating concentrations of IMP. Therefore, the k'_5 in the kinetic mechanism is a complex rate constant, consisting of hydrolysis, XMP dissociation and IMP association.

All 13 microscopic rate constants in minimal kinetic mechanism shown in Figure 6A were treated as adjustable model parameters. Empirical confidence intervals were computed according to the method described elsewhere.[S16] The most plausible values of rate constants were computed as geometric means (GM) and the associated geometric standard deviations (GSD) from combinatorial replicates (n = 12 for wild-type and n = 18 for the mutant enzyme). In both wild-type and mutant enzymes, only the lower limits[S16–S18] could be

determined for the NADH binding steps k_4 and k_{-4}. For these two rate constants, the most plausible values were computed as geometric means and the associated geometric standard deviations of lower limit estimates.

1.6.2 Analysis of initial rate data

Initial rate data were analyzed by using a minor variation of the general numerical method described in our previous work.[S19] Unlike in the original report,[S19] in this research the steady-state concentrations of all substrates and products were assumed to be fixed constants. In contrast, the steady-state concentration of inhibitors were treated as nonlinear parameters, in order to account for the possibility of inhibitor depletion ("tight-binding").[S20–S22] The global nonlinear fitting model consisted of a system of simultaneous nonlinear algebraic equations listed in section 10, which correspond to the kinetic mechanism shown in Figure 6A.

1.6.3 STD-NMR experiments

NMR spectra were acquired in a Bruker Avance spectrometer operating at 800.13 MHz (1H) and equipped with 5 mm TXI cryoprobe and pulsed field gradients using 0.5-1.0 mM inhibitor (**P32** and **P131**), 10 μM IMPDH and 100 μM IMP in 50 mM deuterated Tris-HCl (pH 8.0), 100 mM KCl, 1 mM DTT. All experiments were performed at 298 K. Saturation of the protein resonances was achieved by 3 s train of Gaussian pulses at - 0.7 ppm and -30 ppm for the on-resonance and off-resonance spectra, respectively and using 1600 scans and 64K time domain points. To suppress a water signal, excitation sculpting with gradients was used. NMR data were processed and analyzed using TOPSPIN (Bruker Biospin Inc.). STD effect was determined using amplification factor (STD-AF) that was obtained by multiplying the relative STD effect of a given hydrogen ($(\mathrm{I_0} - \mathrm{I_{SAT}})/\mathrm{I_0}$) at a given ligand concentrations ($[\mathrm{L}]_\mathrm{T}$) with the molar ratio of ligand in excess relative to the protein ($[\mathrm{L}]_\mathrm{T}/[\mathrm{P}]$):

$$\text{STD-AF} = \frac{(\mathrm{I_0} - \mathrm{I_{SAT}})}{\mathrm{I_0}} \cdot \frac{[\mathrm{L}]_\mathrm{T}}{[\mathrm{P}]} \tag{S3}$$

2 Sequence alignment

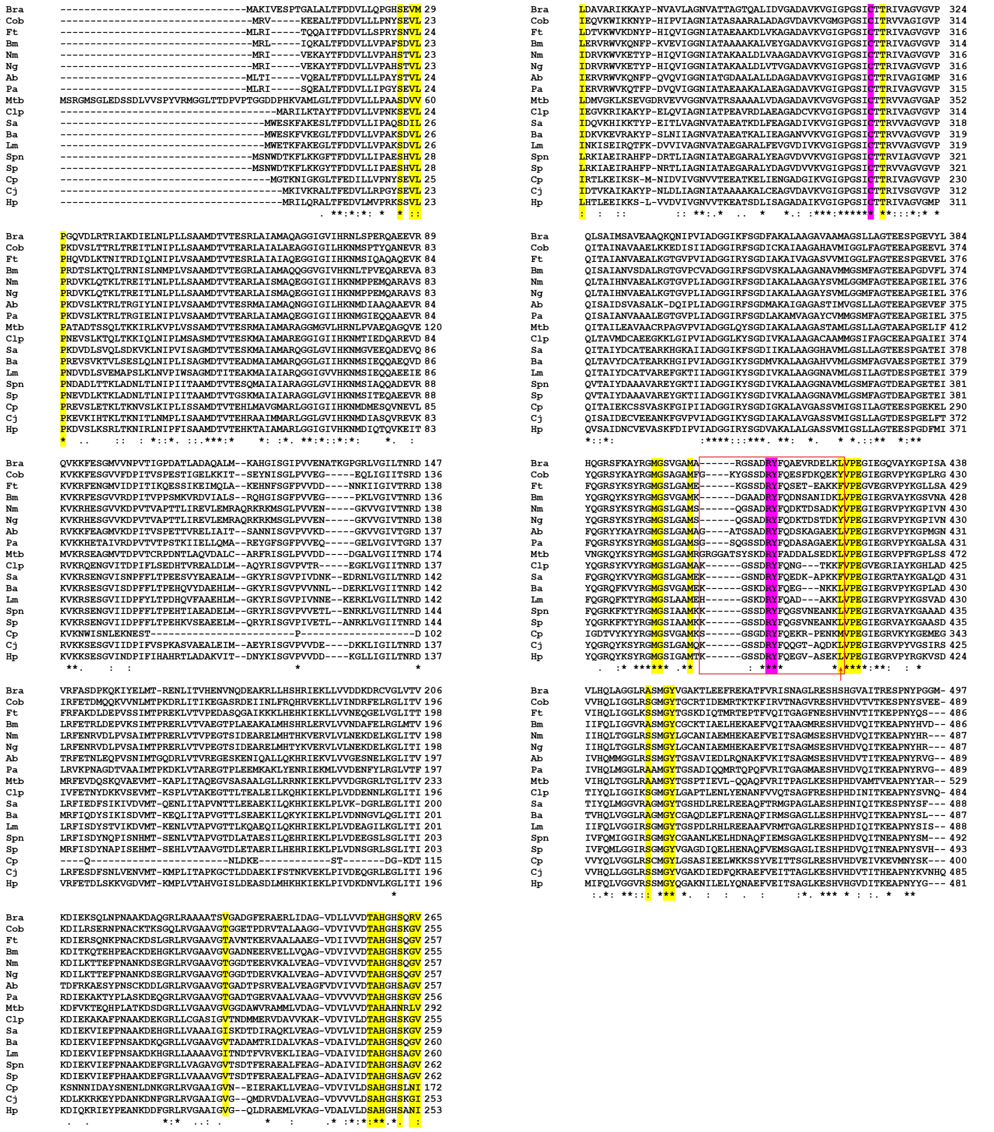

Figure S1: **The sequence alignment of bacterial IMPDHs.** The residues involved in inhibitor binding are highlighted yellow, catalytic residues are highlighted pink, flap region are included in red box and Leu413 is indicated by red arrow. Ab, *Acinetobacter baumannii* (CAP02716.1); Ba, *Bacillus anthracis* (AAP24065); Bra, *Brucella abortus* (YP_223584.1); Bm, *Burkholderia mallei* (ZP_04884831); Cj, *Campylobacter jejuni* (WP_002852968); Clp, *Clostridium perfringens* (ZP_02642385); Cob, *Coxiella burnetii* (NP_820331); Cp, *Cryptosporidium parvum* (XP_625342.1); Ft, *Francisella tularensis* (YP_514129); Hp, *Helicobacter pylori* (WP_001221712.1); Lm, *Listeria monocytogenes* (ZP_05294383); Mtb, *Mycobacterium tuberculosis* guaB2 (CAB01012); Nm, *Neisseria meningitis* (YP_003083209); Ng, *Neisseria gonorrhoeae* (AAW89513); Pa, *Pseudomonas aeruginosa* (NP_252459); Sa, *Staphylococcus aureus* (YP_039865); Sp, *Streptococcus pyogenes* (AAB03846); Spn, *Streptococcus pneumoniae* (ABJ54261).

3 X-Ray crystal structures

3.1 Ten newly described crystal structures in this study

Table S1: X-ray crystal structures of E•IMP•inhibitor complexes reported in this study.

Enzyme	Inhibitor	Structure	PDB ID	Resolution (Å)	$K_{\mathrm{i,app}}$
*Cj*IMPDHΔS	**P176**	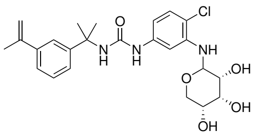	5URQ	2.7	177 ± 34
*Cj*IMPDHΔS	**P182**	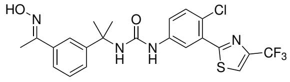	5UQH	2.2	32 ± 6
*Cj*IMPDHΔS	**P200**	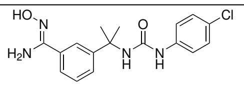	5UQG	2.03	145 ± 40
*Cj*IMPDHΔS	**P225**	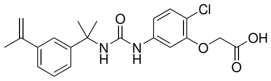	5UQF	2.73	20 ± 10
*Clp*IMPDHΔL	**P176**	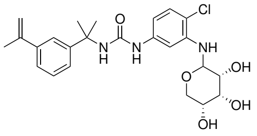	5UWX	1.85	53 ± 4
*Clp*IMPDHΔL	**P178**	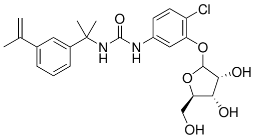	5UXE	2.1	97 ± 13
*Clp*IMPDHΔL	**P182**	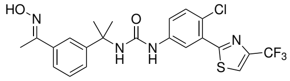	5UZE	2.27	16 ± 3
*Clp*IMPDHΔL	**P200**	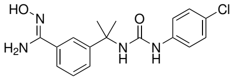	5UZS	2.37	67 ± 10
*Clp*IMPDHΔL	**P221**	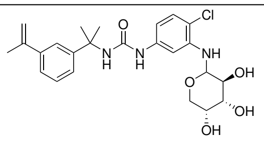	5UZC	1.85	42 ± 4
*Clp*IMPDHΔL	**P225**	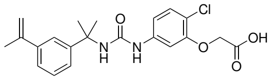	5VSV	2.21	7.4 ± 2.2

Table S2: Crystal and Data Collection and Refinement Statistics.

	*Cj*IMPDHΔS •IMP•**P176**	*Cj*IMPDHΔS •IMP•**P182**	*Cj*IMPDHΔS •IMP•**P200**	*Cj*IMPDHΔS •IMP•**P225**	*Clp*IMPDHΔL •IMP•**P176**
Data collection					
Space group	$P2_1$	$P2_1$	$P2_1$	I422	C2
Cell dimensions,					
a, b, c (Å),	98.49, 141.79, 121.64	98.44, 141.28, 121.06	95.89, 137.96, 116.55	a=b=118.53, 451.30	128.28, 118.94. 96.99,
α, β, γ (°)	90.0, 94.5, 90.0	90.0, 94.4, 90.0	90.0, 96.0, 90.0	α=β=γ =90.0	90.0, 102.9, 90.00
Protein molecules/ASU	8	8	8	3	4
Wavelength (Å)	0.97932	0.97932	0.97934	0.97918	0.97918
Resolution (Å)*	2.70 (2.75-2.70)	2.20 (2.24-2.20)	2.03 (2.07-2.03)	2.72 (2.78-2.72)	1.85 (1.88-1.85)
Unique reflections	91374 (4135)	163771 (7421)	194228 (9561)	43356 (2145)	118158 (5833)
R_{merge} †	0.115 (0.736)	0.090(0.538)	0.119 (0.772)	0.137 (0.757)	0.096 (0.705)
$<I>/<\sigma I>$	10.8 (1.3)	13.8(1.3)	11.6 (1.6)	10.7 (2.5)	16.3 (1.8)
Completeness (%)	98.9 (89.7)	99.3 (89.3)	99.6 (98.9)	99.3 (99.2)	97.5 (96.7)
Redundancy	3.3 (2.7)	4.0 (1.6)	3.7 (3.2)	4.3 (4.2)	5.9 (5.7)
$CC_{1/2}$	0.491	0.704	0.558	0.696	0.804
Refinement					
MR model used	4R7J	4R7J	4R7J	4R7J	4Q32
Resolution (Å)	2.70 (2.71-2.70)	2.20(2.24-2.20)	2.03 (2.05-2.03)	2.72 (2.78-2.72)	1.85 (1.87-1.85)
Reflections: work/test set	86687/4505	150699/7426	184481/9631	41112/2092	112143/5793
R_{work}/R_{free}‡	0.180/0.235	0.172/0.213	0.166/0.203	0.175/0.228	0.163/0.197
No. of atoms: protein/ligands§/water	21152/456/17	20304/480/593	211745/392/769	7908/244 /70	10131/276/477
Mean B factor (Å^2): protein/ligands/water	64.4/58.8/49.4	39.6/42.2/35.8	30.6/27.4/32.5	45.1/62.1/40.4	43.2/47.6/42.3
RMSD Bond lengths (Å)/Bond angles (°)	0.008/1.101	0.003/0.855	0.008/1.152	0.008/1.079	0.008/1.021
Ramachandran plot: most favored/outliers, %	94.36/0.75	95.99/0.67	96.02/0.61	95.49/0.38	96.35/0.53
PDB code	5URQ	5UQH	5UQG	5UQF	5UWX
Crystallization conditions	10 % (v/v) 2-propanol, 0.1 M Tris pH 8.5, 16°C	10 % (v/v) 2-propanol, 0.1 M Tris pH 8.5, 16°C	5 % (w/v) 2-propanol, 0.1 M HEPES pH 7.5, 16°C	1.2 M sodium dihydrogen phosphate, 0.8 M potassium hydrogen phosphate, 0.1 M CAPS pH 10.5, 0.2 M lithium sulfate, 16°C	0.2M ammonium acetate, 0.1M Bis-Tris pH 6.5, 45% MPD, 16°C

ASU, Asymmetric Unit. *Values in parentheses correspond to the highest-resolution shell. †$R_{merge} = \Sigma_{hkl}\Sigma_i|I_i(\text{hkl}) - <\text{I(hkl)}>|/\Sigma_{hkl}\Sigma_i|<I_i(\text{hkl})>|$, where I_i(hkl) is the intensity for the ith measurement of an equivalent reflection with indices h, k, and l. ‡$R = \Sigma_{hkl}||F_{obs}| - |F_{calc}||/\ \Sigma_{hkl}\ |F_{obs}|$, where F_{obs} and F_{calc} are observed and calculated structure factors, respectively. R_{free} is calculated analogously for the test reflections, which were randomly selected and excluded from the refinement. §Ligands include all atoms excluding protein and water atoms.

Table S2: Crystal and Data Collection and Refinement Statistics - continued.

	*Clp*IMPDHΔL •IMP•**P182**	*Clp*IMPDHΔL •IMP•**P200**	*Clp*IMPDHΔL •IMP•**P178**	*Clp*IMPDHΔL •IMP•**P221**	*Clp*IMPDHΔL •IMP•**P225**
Data collection					
Space group	P1	$P2_12_12$	C2	C2	P1
Cell dimensions,					
a, b, c (Å),	62.78, 77.66, 80.32	111.12, 144.40, 87.53	127.94, 119.10, 96.97	128.02, 119.31, 97.20	62.86, 77.64, 78.97
α, β, γ (°)	110.0, 104.0, 105.5		90.0, 102.9, 90.0	90.0, 102.8, 90.0	110.8, 104.1, 105.4
Protein molecules/ASU	4	4	4	4	4
Wavelength (Å)	0.97919	0.97919	0.97918	0.97918	0.97919
Resolution (Å)*	2.27 (2.31-2.27)	2.35 (2.39-2.35)	2.10 (2.14-2.10)	1.85 (1.88-1.85)	2.20 (2.24-2.20)
Unique reflections	60049 (3604)	59812 (2954)	80071 (3680)	120850 (5984)	59509 (2929)
R_{merge} †	0.083 (0.502)	0.125 (0.699)	0.086 (0.614)	0107 (0.797)	0.148 (0.689)
$<I>/<\sigma I>$	8.9 (2.0)	17.0 (2.3)	11.1 (2.0)	15.1 (1.0)	6.3 (1.6)
Completeness (%)	97.3 (97.0)	99.9 (100)	94.4 (88.1)	99.3 (98.1)	96.3 (94.1)
Redundancy	2.1 (2.1)	8.0 (7.9)	2.7 (2.2)	5.0 (4.7)	2.0 (1.8)
$CC_{1/2}$	0.784	0.861	0.625	0.67	0.561
Refinement					
MR model used	4Q32	4Q33	4Q32	4Q32	4Q32
Resolution (Å)	2.27 (2.29-2.27)	2.35 (2.41-2.35)	2.07 (2.09-2.07)	1.85 (1.87-1.85)	2.20 (2.24-2.20)
Reflections: work/test set	56563/2855	57229/2838	75965/3908	114873/5934	56217/3003
R_{work}/R_{free}‡	0.194/0.255	0.170/0.238	0.173/0.223	0.171/0.201	0.198/0.241
No. of atoms: protein/ligands§/water	10075/232/156	10214/304/147	10115/251/232	10143/174/409	10085/204/212
Mean B factor (Å^2): protein/ligands/water	51.6/49.6/42.3	38.8/41.4/32.4	41.2/39.4/35.5	43.7/46.6/42.5	40.6/31.4/33.4
RMSD Bond lengths (Å)/Bond angles (°)	0.009/1.224	0.008/1.020	0.007/0.919	0.011/1.2089	0.007/0.956
Ramachandran plot: most favored/outliers, %	93.63/0.96	95.91/0.45	96.22/0.39	96.48/0.38	95.99/0.99
PDB code	5UZE	5UZS	5UXE	5UZC	5VSV
Crystallization conditions	20 % PEG600, 50mM ADA pH 6.8, 16°C	0.05 M acetate pH 5.2, 45% PEG 200, 16°C	0.2M ammonium acetate, 0.1M Bis-Tris pH 6.5, 45% MPD, 16°C	0 0.2M ammonium acetate, 0.1M Tris pH 8.5, 45% MPD, 16°C	17.1% PEG600, 50mM Bicine pH 8.4, 4.3% PEG2000MME, 16°C

ASU, Asymmetric Unit. *Values in parentheses correspond to the highest-resolution shell. $^\dagger R_{merge} = \Sigma_{hkl}\Sigma_i|I_i(\text{hkl}) - <\text{I(hkl)}>|/\Sigma_{hkl}\Sigma_i|<I_i(\text{hkl})>|$, where I_i(hkl) is the intensity for the ith measurement of an equivalent reflection with indices h, k, and l. $^\ddagger \text{R} = \Sigma_{hkl}||F_{obs}| - |F_{calc}||/ \Sigma_{hkl} |F_{obs}|$, where F_{obs} and F_{calc} are observed and calculated structure factors, respectively. R_{free} is calculated analogously for the test reflections, which were randomly selected and excluded from the refinement. §Ligands include all atoms excluding protein and water atoms.

3.2 List of 25 analyzed crystal structures

X-ray crystal structures of E•IMP•inhibitor complexes analyzed in this study are listed in Table S3.

Table S3: X-ray crystal structures of E•IMP•inhibitor complexes analyzed in this study.

Enzyme	Inhibitor	PDB	Resolution (Å)	Active site number	Reference
*Ba*IMPDHΔL	**A110**	4MYA	1.9	2	[S1]
*Ba*IMPDHΔL	**C91**	4MY9	2.59	8	[S1]
*Ba*IMPDHΔL	**D67**	4QM1	2.8	4	[S1]
*Ba*IMPDHΔL	**P32**	4MYX	2.7	8	[S1]
*Ba*IMPDHΔL	**P68**	4MY1	2.6	8	[S1]
*Ba*IMPDHΔL	**P176**	7MTX	2.44	8	[S23]
*Ba*IMPDHΔL	**P178**	5URS	2.39	8	[S23]
*Ba*IMPDHΔL	**P182**	5UUV	2.75	4	[S23]
*Ba*IMPDHΔL	**P200**	5UUZ	2.5	8	[S23]
*Ba*IMPDHΔL	**P221**	7MTU	2.34	8	[S23]
*Ba*IMPDHΔL	**Q21**	4MY8	2.29	4	[S1]
*Cj*IMPDHΔS	**C91**	4MZ8	2.5	4	[S1]
*Cj*IMPDHΔS	**P12**	4MZ1	2.4	3	[S1]
*Cj*IMPDHΔS	**P176**	5URQ	2.7	8	this study
*Cj*IMPDHΔS	**P182**	5UQH	2.2	8	this study
*Cj*IMPDHΔS	**P200**	5UQG	2.03	8	this study
*Cj*IMPDHΔS	**P225**	5UQF	2.73	3	this study
*Clp*IMPDHΔL	**A110**	4Q33	2.88	8	[S1]
*Clp*IMPDHΔL	**C91**	4Q32	2.79	4	[S1]
*Clp*IMPDHΔL	**P176**	5UWX	1.85	4	this study
*Clp*IMPDHΔL	**P178**	5UXE	2.1	4	this study
*Clp*IMPDHΔL	**P182**	5UZE	2.27	4	this study
*Clp*IMPDHΔL	**P200**	5UZS	2.37	4	this study
*Clp*IMPDHΔL	**P221**	5UZC	1.85	4	this study
*Clp*IMPDHΔL	**P225**	5VSV	2.21	4	this study

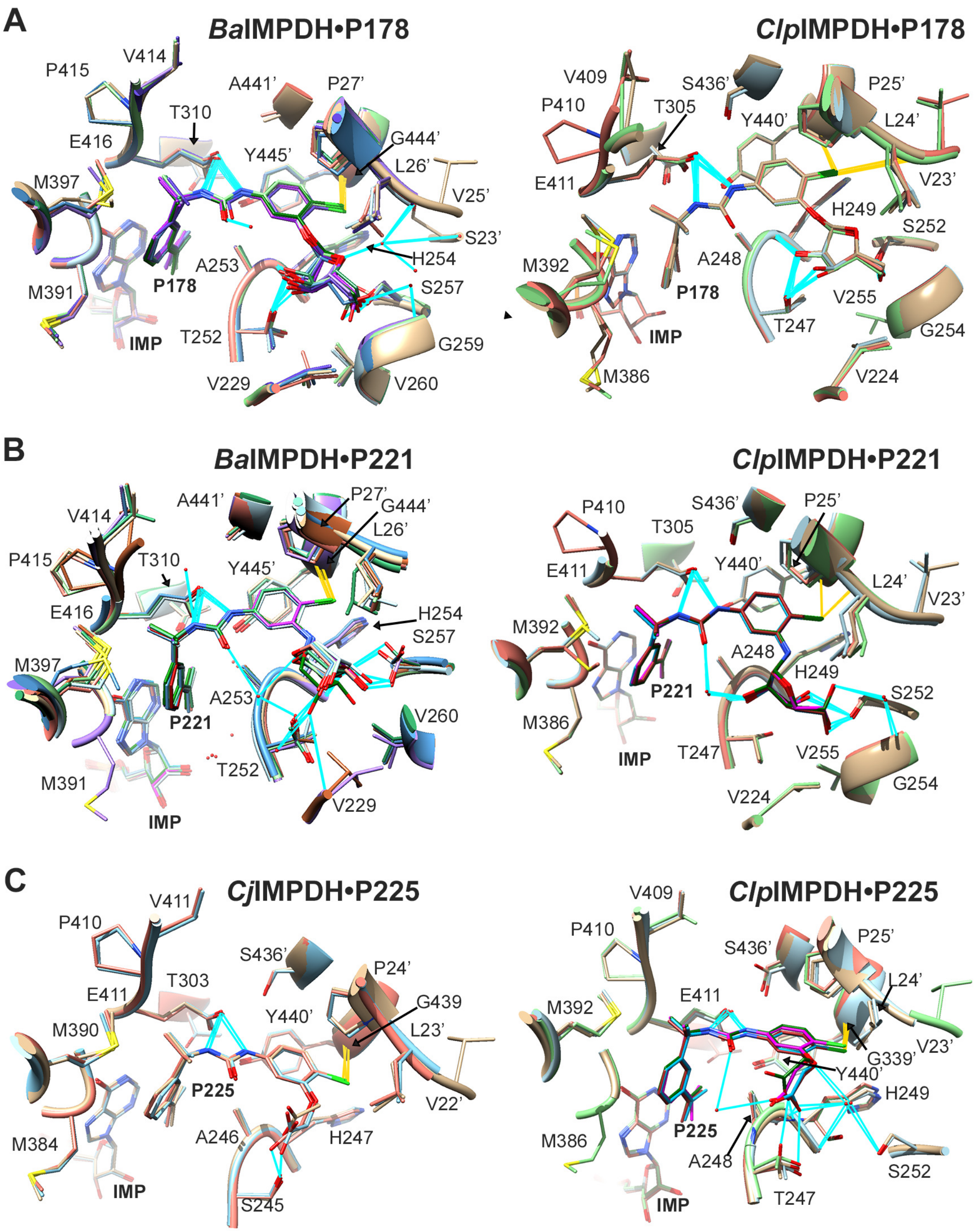

Figure S2: **Structures of inhibitor complexes. A.** The structures of E•IMP•**P178** complexes. The asymmetric units of *Ba-* (PDB:5URS) and *Clp*IMPDH (PDB:5UXE) contained 8 and 4 active sites, respectively, consisting of two subunits. The IMP containing subunits of each active sites within a given structure were overlayed. Hydrogen bonds are depicted in cyan and halogen bonds are shown in gold. **B.** The structures of E•IMP•**P221** complexes. The asymmetric units of *Ba-* (PDB:7MTU) and *Clp*IMPDH (PDB:5UZC) contained 8 and 4 active sites, respectively. **C.** The structures of E•IMP•**P225** complexes. The asymmetric units of *Cj-* (PDB:5UQF) and *Clp*IMPDH (PDB:5UVSV) contained 3 and 4 active sites, respectively. Panels A-C were rendered in UCSF Chimera.[S24]

4 STD-NMR experiments

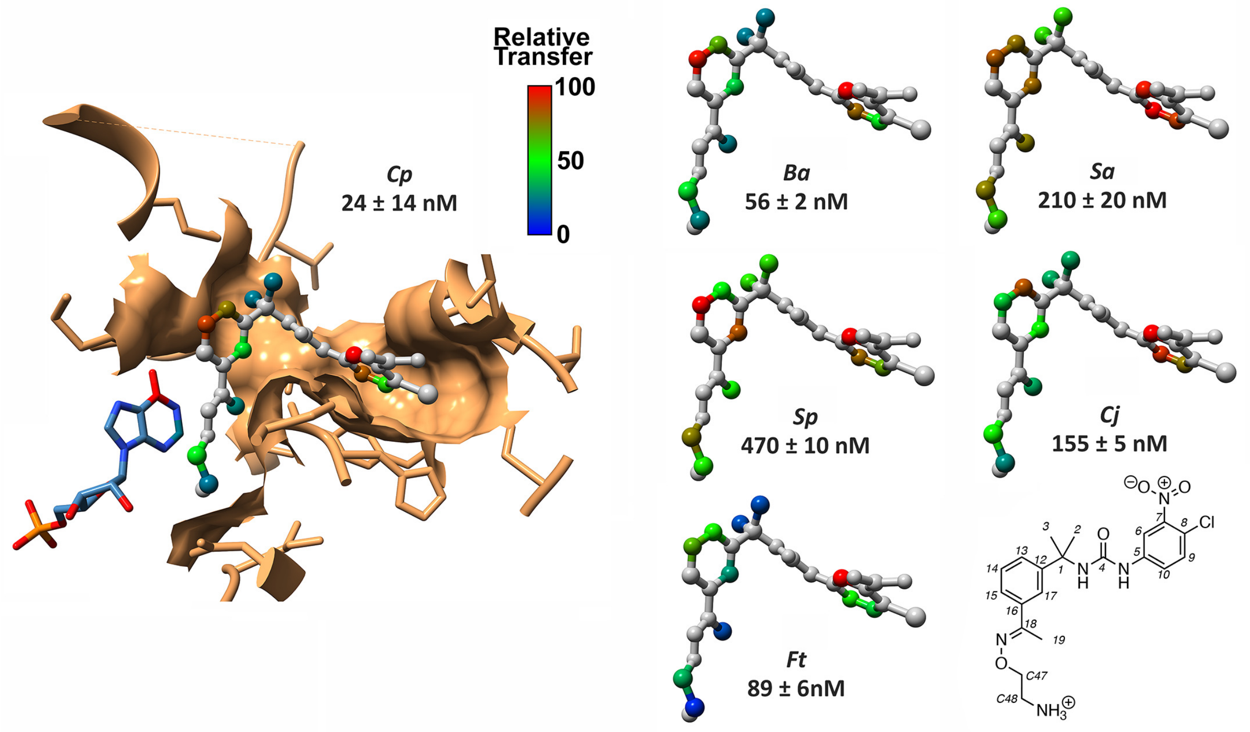

Figure S3: **Inhibitor P131 binding epitopes measured by STD-NMR.** The E•IMP•**P131** complex of *Cp*IMPDH (PDB 4RV8, AD active site) is shown.[S25] Surfaces denote where the protein (coral) contacts the inhibitor. **P131** is colored by relative STD signals. Structure of **P131** is numbered as found in 4RV8. The values of K_{iapp} are shown. Figure rendered in UCSF Chimera.[S11]

Table S4: STD data for the binding of **P32** to bacterial IMPDHs.

Atom	Peak (ppm)	Relative Transfer (%)					
		Cp (N=3)	Sp (N=2)	Ba (N=3)	Sa (N=1)	Ft (N=1)	Cj (N=1)
C2 and C3	1.59	25 ± 1	46 ± 3	41 ± 15	20	16	31
C6	7.61	56 ± 1	72 ± 3	69 ± 4	46	44	59
C9	7.5	51 ± 6	78 ± 1	77 ± 7	51	50	63
C10	7.42	59 ± 3	86 ± 4	87 ± 3	73	78	82
C13	7.25	100 ± 1	99 ± 1	91 ± 5	97	93	96
C14	7.39	56 ± 1	81 ± 7	93 ± 8	69	67	80
C15	7.33	98 ± 3	100 ± 1	95 ± 6	98	90	97
C17	7.32	86 ± 4	96 ± 2	94 ± 9	100	100	100
C19	2.21	42 ± 2	76 ± 1	50 ± 4	56	54	70

Table S5: STD data for the binding of **P131** to bacterial IMPDHs.

Atom	Peak (ppm)	Relative Transfer (%)					
		Cp	Sp	Ba	Sa	Ft	Cj
C6	7.91	100	100	100	95	100	100
C17	7.63	43	84	38	80	28	52
C9	7.52	57	69	45	88	40	77
C10	7.46	83	80	80	100	53	91
C13	7.38	77	48	67	78	54	84
C14	7.37	88	0	96	83	66	40
C19	4.34	26	53	24	76	15	32
C47	3.29	22	55	25	60	8	26
C48	2.24	45	76	42	76	33	53
C2 and C3	1.6	23	58	23	59	15	32

5 Substrate kinetic properties of IMPDH mutants

5.1 Theory

Substrate kinetics of IMPDH essentially follows the the Order Bi Bi[S26] mechanism accompanied by uncompetitive product inhibition by NAD^+. The requisite initial rate equation has been derived by Cleland and Cook [p. 173, Eqn (6-103)][S27] in the double-reciprocal form. An algebraic rearrangement yields Eqn (S4), where [E] is the enzyme concentration; [A] and [B] are the concentrations of IMP and NAD^+, respectively; k_{cat} is the turnover number; K_{dA} is the dissociation equilibrium constants of the E•IMP complex; K_{mA} and K_{mB} are the Michaelis constants of IMP and NAD^+, respectively; and K_{iB} is the uncompetitive substrate inhibition constant of NAD^+.

$$v = [\mathrm{E}]\, k_{cat} \frac{[\mathrm{A}]\,[\mathrm{B}]}{K_{dA}\, K_{mB} + K_{mB}\,[\mathrm{A}] + [\mathrm{A}]\,[\mathrm{B}]\,\left(1 + [\mathrm{B}]/K_{iB}\right)} \tag{S4}$$

Eqn (S4) can be further rearranged to define the apparent Michaelis constants of IMP and NAD^+. At an arbitrary fixed concentration of IMP ([A]), the initial reaction rate depends on the concentration of NAD^+ ([B]) as is shown in Eqns (S5)–(S8), where $k_{cat}^{*,B}$ is the apparent turnover number; K_{mB}^* is the apparent Michaelis constant of NAD^+ at the given level of [A]; and K_{iB}^* is the apparent substrate inhibition constant of NAD^+.

$$v = [\mathrm{E}]\, k_{cat}^{*,B} \frac{[\mathrm{B}]}{K_{mB}^* + [\mathrm{B}]\,\left(1 + [\mathrm{B}]/K_{iB}\right)} \tag{S5}$$

$$k_{cat}^{*,B} = k_{cat} \frac{[\mathrm{A}]}{[\mathrm{A}] + K_{dA}} \tag{S6}$$

$$K_{mB}^* = K_{mB} \frac{[\mathrm{A}] + K_{dA}}{[\mathrm{A}] + K_{mA}} \tag{S7}$$

$$K_{iB}^* = K_{iB}\,\left(1 + K_{mA}/[\mathrm{A}]\right) \tag{S8}$$

Note that the apparent Michaelis constant of NAD^+ approaches the ratio K_{dA}/K_{mA} at [A] approaching zero. In independent experiments (results not shown) we have established that the K_{dA}/K_{mA} ratio is approximately equal to 1.2 for the wild-type enzyme and 2.3 for the L413A mutant. Importantly, the algebraic form of Eqn (S7) shows that at sufficiently high [A] concentrations (i.e. [A] >> K_{dA} and [A] >> K_{mA}), the apparent Michaelis constant for NAD^+ (a) approaches the true Michaelis constant for NAD^+ and (b) is no longer sensitive to [A].

Similarly, at an arbitrary fixed concentration of NAD^+ ([B]), the initial reaction rate depends on the concentration of IMP ([A]) as is shown in Eqns (S9)–(S11), where $k_{cat}^{*,A}$ is again the apparent turnover number and K_{mA}^* is the apparent Michaelis constant of IMP at the given level of NAD^+.

$$v = [E]\, k_{cat}^{*,A} \frac{[A]}{K_{mA}^* + [A]} \tag{S9}$$

$$k_{cat}^{*,A} = k_{cat} \frac{[B]}{K_{mB}^* + [B]\ (1 + [B]/K_{iB})} \tag{S10}$$

$$K_{mA}^* = K_{dA} \frac{K_{mB}^* + [B]\, K_{mA}/K_{dA}}{K_{mB}^* + [B]\ (1 + [B]/K_{iB})} \tag{S11}$$

5.2 Determination of apparent Michaelis constants

5.2.1 Representative example

Relying on the theoretical analysis presented above, we have determined the apparent Michaelis constants K_{mA}^* for all four enzyme variants at suitably chosen fixed concentrations of NAD^+, and also the apparent Michaelis constants K_{mB}^* at suitably chosen fixed concentrations of IMP. An illustrative example involving the L413F mutant is shown in Figure S4.

The left-hand panel in Figure S4 shows the variation of in the initial reaction rates in

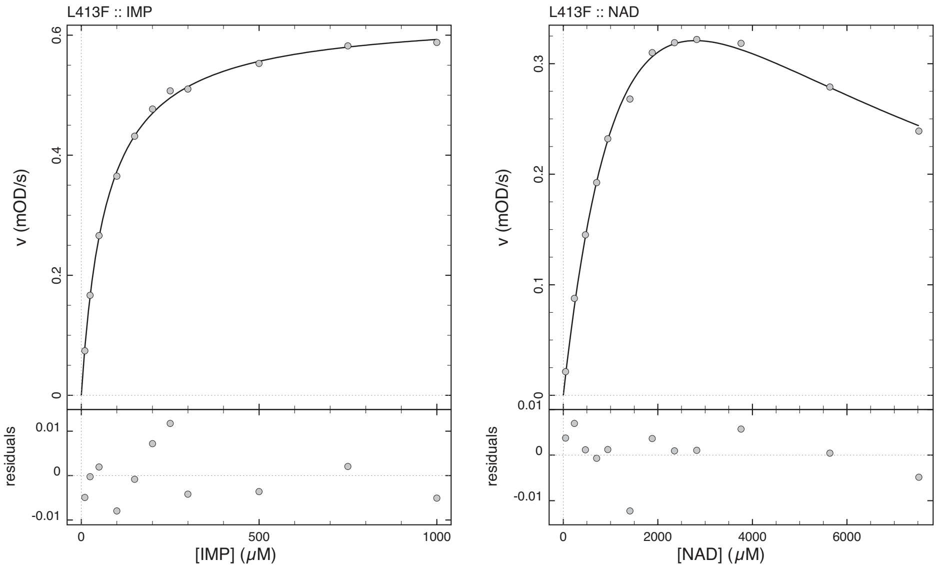

Figure S4: **A representative example of determining the apparent Michaelis constants K^*_{mA} and K^*_{mB} for the L413F mutant. For details see text**

dependence on the concentration of IMP at a fixed concentration NAD^+ [B] = 2.5 mM. The data were fit to Eqn (S9). The best-fit value of the apparent Michaelis constant for IMP was $K^*_{\mathrm{mA}} = (70.1 \pm 2.1)$ [65.6, 74.9] μM, where the values in square brackets represent the 95% confidence level interval determined by the profile-t method of Bates and Watts.[S28,S29]

The right-hand panel in Figure S4 shows the variation of in the initial reaction rates in dependence on the concentration of NAD^+ at a fixed concentration IMP [A] = 1.0 mM. The data were fit to Eqn (S5). The best-fit value of the apparent Michaelis constant for NAD^+ was $K^*_{\mathrm{mB}} = (2060 \pm 240)$ [1600, 2750] μM, where the values in square brackets again represent the 95% confidence level interval.

5.2.2 Summary of results

The results are summarized in Table S6 and Table S7. In both tables, the second column labeled n represents the number of independent replicates similar to the experiment depicted in Figure S4. The requisite apparent K_{m} values were determined by the *global*[S30] fit of all combined replicates. The third column shows the concentration of the fixed component (i.e., the fixed concentration of IMP in the determination of K^*_{mB} and the fixed concentration of

NAD$^+$ in the determination of K^*_{mA}) in each replicate. In the fourth column, the values in square brackets are 95%-confidence level intervals determined by the profile-t method of Bates and Watts.[S28,S29]

Table S6: Determination of apparent Michaelis constants for IMP: summary of results. For details see text.

	n	[NAD$^+$] (μM)	K_{mA} (μM)
WT	3	2500, 2225, 2059	58.2 [54.9 , 61.6]
L413A	2	2500, 2080, 2600	57.6 [51.4 , 64.4]
L413V	1	2500	85.1 [73.7 , 98.0]
L413F	1	2500	70.1 [65.6 , 74.9]

Table S7: Determination of apparent Michaelis constants for NAD$^+$: summary of results. For details see text.

	n	[IMP] (μM)	K_{mB} (μM)
WT	3	1000, 1000, 1000	683 [601 , 779]
L413A	2	1000, 1120	1100 [808 , 1550]
L413V	2	1000, 1000	3650 [2460 , 6100]
L413F	1	1000	2060 [1600 , 2750]

Note that the apparent Michaelis constants for IMP determined at fixed NAD$^+$ concentrations (K^*_{mA}) are nearly identical for all four enzyme forms. In contrast, the apparent Michaelis constants for NAD$^+$ determined at fixed IMP concentrations (K^*_{mB}) show a significant sensitivity to the Leu413 mutations. The apparent K_{m} values listed in the tables above were utilized in the design of experiments for the determination of apparent inhibition constants $K_{\mathrm{i,app}}$ (see section below).

6 Effect of Leu413 mutations on inhibition

6.1 Inhibitor structures

Chemical structures of the 23 IMPDH inhibitors utilized to characterize the effects of the Leu413 mutations are shown in Figure S5.

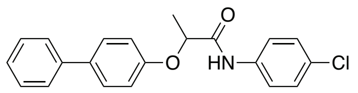

A50

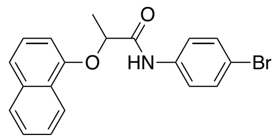

A61

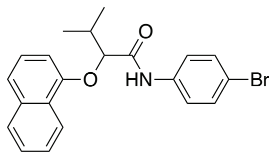

A68

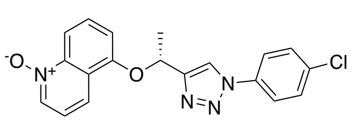

A109

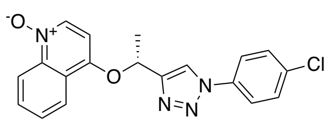

A110

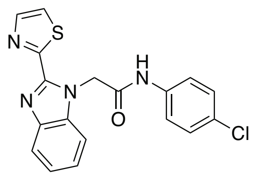

C61

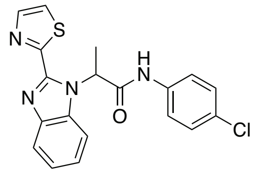

C74

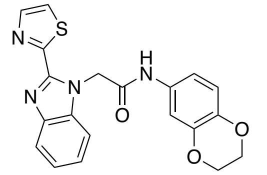

C83

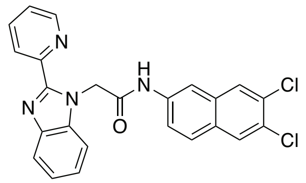

C85

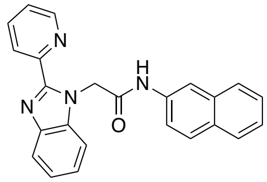

C91

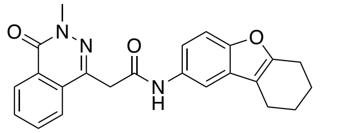

D67

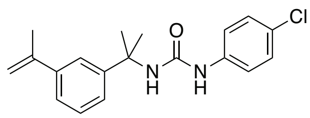

P11

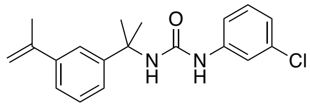

P15

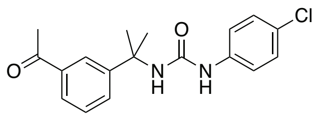

P20

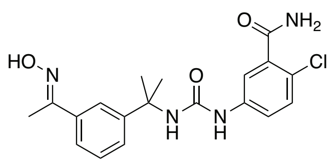

P32

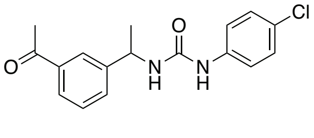

P52

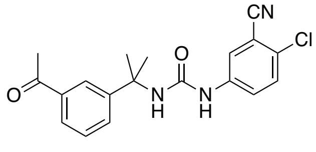

P63

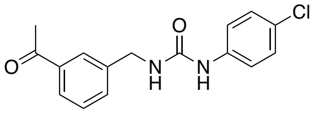

P77

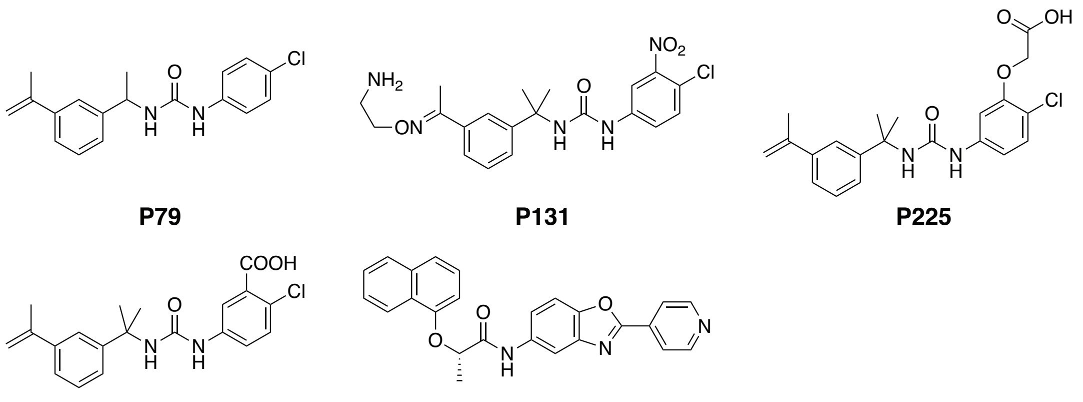

Figure S5: **Chemical structures of the 23 IMPDH inhibitors utilized to characterize the effects of the Leu413 mutations.**

6.2 Reagent concentrations

Reagent concentrations utilized in the determination apparent K_{i} values are listed in Table S8.

Table S8: Reagent concentrations in the determination apparent K_{i} values. For details see text.

	WT	L413A	L413V	L413F
[E] (nM)	20	40	20	20
[IMP] (μM)	600	700	850	700
[NAD] (μM)	1800	2400	3000	2500

The IMP concentrations were chosen such that [IMP] >> K^{*}_{mIMP}. NAD^{+} concentrations were chosen where enzymatic activities reached maximum.

6.3 Determination of initial reaction rates

A representative set of three examples from the determination of initial rates is shown in Figure S6. Note that the instantaneous reaction rate (middle column in Figure S6) changes by approximately 30–50% over the course of the 15-minute assay. This means that a linear fit to the complete curve would introduce systematic distortions into the (presumably) ini-

tial reaction rates. Therefore, initial reaction rates were determined by *piecewise-linear fit* with $n = 3$ equal length segments. Specifically, each 15-minute progress curve was divided into three 5-minute increments, and initial rates were determined by linear least-squares regression of of the first segment only ($t < 5$ min).

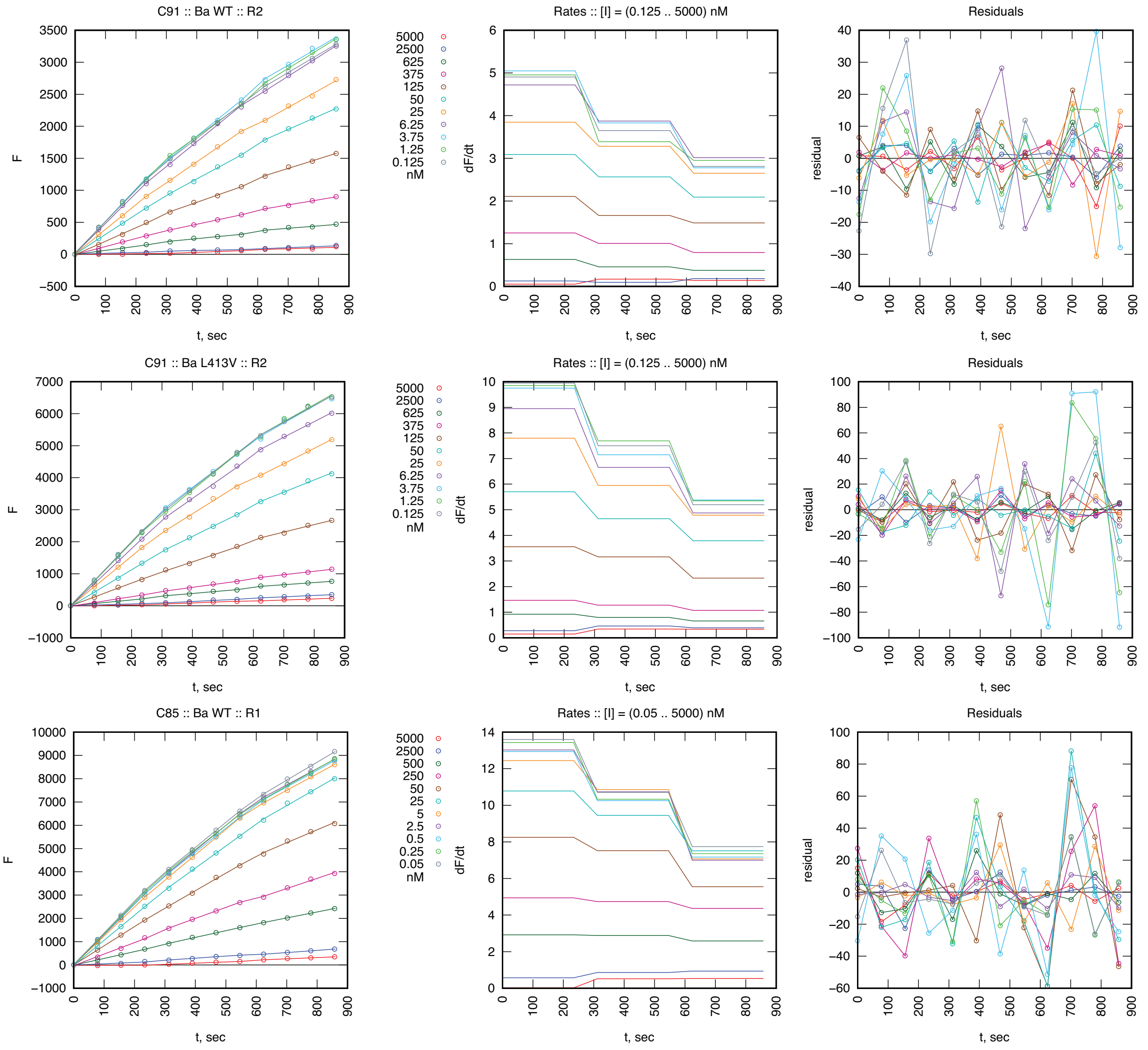

Figure S6: **Representative reaction progress curves used to determine initial reaction rates in the analysis of flap mutation sensitivity.** *Top row:* Inhibitor **C91** vs. wild-type enzyme, replicate 2 of 2. *Middle row:* Inhibitor **C91** vs. L413 mutant, replicate 2 of 2. *Bottom row:* Inhibitor **P85** vs. L413 mutant, replicate 1 of 2. *Left column:* Raw experimental data and results of a piecewise linear fit. *Middle column:* Instantaneous reaction rates. *Right column:* Residual plots.

6.4 Analysis of initial reaction rates

Initial reaction rates determined by the piecewise-linear fit of reaction progress curves, as described in the previous section, were analyzed by nonlinear least-squares fit to Eqn (S12), where v_0 is the initial reaction rate in the absence of inhibitors; [E] is the total or analytic concentration of the enzyme; [I] is the varied concentration of the inhibitor; and $K_{\mathrm{i,app}}$ is the apparent inhibition constant.[S22] Note that, unlike IC_{50}, $K_{\mathrm{i,app}}$ depends only on the substrate concentrations utilized in the assay, but does not depend on the active enzyme concentration. In contrast, $\mathrm{IC}_{50} = K_{\mathrm{i,app}} + [\mathrm{E}]/2$.[S31]

$$v = v_0 \frac{[\mathrm{E}] - [\mathrm{I}] - K_{\mathrm{i,app}} + \sqrt{([\mathrm{E}] - [\mathrm{I}] - K_{\mathrm{i,app}})^2 + 4\,[\mathrm{E}]\,K_{\mathrm{i,app}}}}{2\,[\mathrm{E}]} \tag{S12}$$

In Eqn (S12), the active enzyme concentration [E] was treated either as fixed constant (model "A"), or as an optimized model parameter (model "B"), according to the general data-analytic approach reported earlier.[S12]

Model selection consisted of three separate steps. First, the acceptability of of models "A" vs. "B" was evaluated by the Akaike Information Criterion (AIC)[S32] and the Bayesian Information Criterion (BIC).[S33] Second, if model "B" (optimized [E]) was preferred by both AIC and BIC, we examined whether or not the confidence interval for [E] computed according to the profile-t method of Bates and Watts[S28,S29] was closed at the 68% confidence level (formally corresponding to one standard deviation). Third, if and when the confidence interval for [E] was actually closed, in that both the upper bound and lower bound were both determined at $P = 68\%$, we examined the best fit value of [E] and compared it with the nominal value determined by the Bradford assay. Model "B" was then accepted if and only if the best-fit value of [E] fell within 0.1-fold and 2.0-fold of the nominal value.

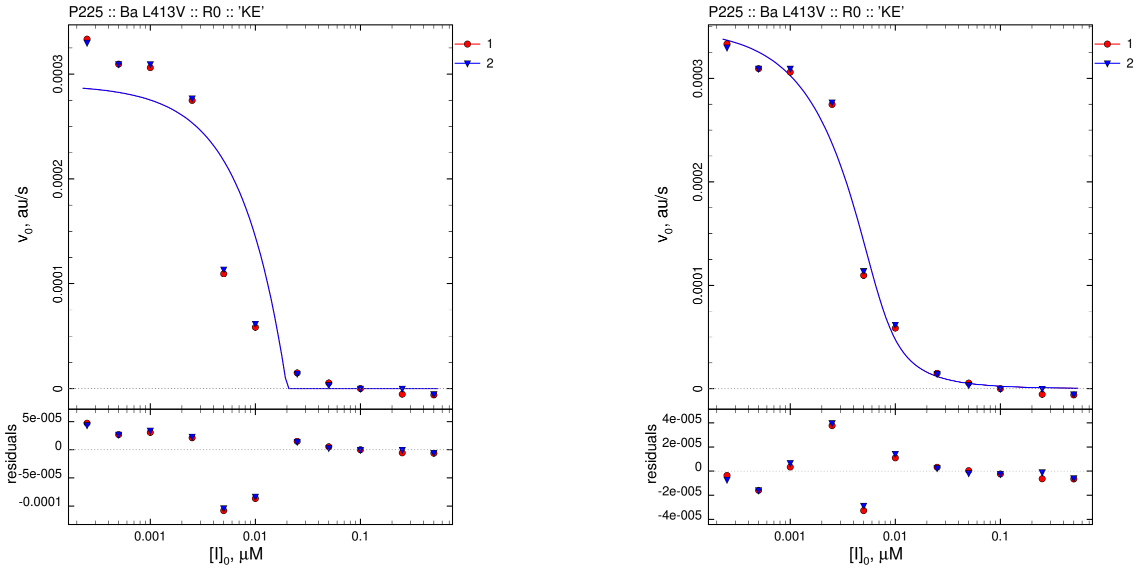

Model "A": fixed [E] Model "B": optimized [E]

Figure S7: **Representative example of model selection.** *Left*: model "A" - fixed [E] = 20 nM. *Left*: model "B" - optimized [E] = 6.9 $[6.0, 7.7]$ nM. The values in square brackets represent the 68% confidence interval (formally corresponding to one standard deviation).

A representative example showing the results of model selection according to the procedure described above is shown in Figure S7. According to model "A" (fixed [E] = 20 nM, left-hand panel), the best-fit value of $K_{\mathrm{i,app}}$ could not be determined. [1] According to model "B" (optimized [E]), the best-fit value of the apparent inhibition constant was $K_{\mathrm{i,app}} = 0.6$ $[0.4, 0.9]$ nM, where values in square brackets represent the 68% confidence interval. The best-fit value of the active enzyme concentration was [E] = 6.9 $[6.0, 7.7]$ nM, which suggests that this particular preparation of the L413V mutant enzyme was approximately $6.9/20.0 \times 100 = 35$ % catalytically active. These results illustrate the importance of appropriately treating the active enzyme concentration either as a fixed parameter, or as an optimized parameter, according to the general method reported elsewhere.[S12] The summary of best-fit values of $K_{\mathrm{i,app}}$ according to model "A" or "B", as appropriate in any particular case, is shown in Table S9.

[1] Nominally, the best-fit value was $K_{\mathrm{i,app}} = 10^{-12}$ nM, but this was actually the arbitrary lower limit imposed on $K_{\mathrm{i,app}}$ in the least-squares regression.

Table S9: Apparent inhibition constants $K_{\mathrm{i,app}}$ (nM) determined by the fit of the initial rates to Eqn (S12). The values in squares brackets represent 68% confidence level intervals determined by the profile-t method of Bates and Watts.[S28,S29]

	$K_{\mathrm{i,app}}$ (nM)			
Compound	wild-type	L413A	L413F	L413V
**A109**	69 [61 , 79]	61 [53 , 70]	57 [48 , 67]	85 [75 , 98]
**A110**	81 [78 , 85]	75 [72 , 78]	72 [69 , 76]	144 [135 , 154]
**A50**	331 [307 , 356]	116 [98 , 138]	187 [171 , 205]	283 [250 , 320]
**A61**	145 [127 , 164]	111 [96 , 127]	83 [75 , 93]	115 [104 , 128]
**A68**	76 [68 , 85]	58 [52 , 66]	36 [30 , 42]	33 [29 , 37]
**C61**	107 [99 , 117]	32 [31 , 33]	47 [45 , 49]	94 [92 , 97]
**C74**	62 [59 , 66]	67 [64 , 70]	45 [42 , 47]	134 [128 , 139]
**C83**	707 [633 , 790]	159 [146 , 173]	274 [253 , 296]	456 [426 , 489]
**C85**	102 [93 , 112]	10 [8 , 13]	28 [25 , 30]	109 [98 , 122]
**C91**	67 [61 , 72]	7 [5 , 9]	22 [21 , 23]	67 [65 , 70]
**D67**	698 [608 , 804]	269 [235 , 307]	272 [248 , 299]	615 [557 , 679]
**P11**	6.8 [5.9 , 7.7]	1.2 [1.1 , 1.4]	3.0 [2.6 , 3.5]	6.5 [6.0 , 7.0]
**P131**	94 [89 , 99]	22 [19 , 24]	49 [45 , 54]	64 [60 , 69]
**P138**	18 [16 , 20]	3 [2 , 4]	11 [11 , 12]	15 [14 , 16]
**P15**	283 [265 , 302]	86 [78 , 96]	126 [118 , 134]	178 [138 , 230]
**P20**	53 [49 , 58]	15 [14 , 16]	33 [32 , 35]	43 [41 , 44]
**P225**	0.43 [0.26 , 0.64]	0.18 [0.04 , 0.38]	0.27 [0.11 , 0.49]	0.64 [0.41 , 0.94]
**P32**	52 [48 , 58]	15 [13 , 18]	36 [32 , 42]	40 [35 , 46]
**P52**	70 [65 , 74]	73 [69 , 76]	80 [75 , 85]	95 [92 , 97]
**P63**	67 [63 , 72]	12 [9 , 16]	36 [35 , 38]	44 [40 , 48]
**P77**	2010 [1920 , 2100]	838 [807 , 869]	1190 [1130 , 1250]	1530 [1450 , 1620]
**P79**	173 [164 , 182]	266 [238 , 297]	203 [185 , 224]	135 [129 , 141]
**Q21**	63 [59 , 67]	54 [51 , 57]	53 [49 , 58]	81 [75 , 87]

6.5 Sensitivity of $K_{\mathrm{i,app}}$ to mutations

Sensitivity of $K_{\mathrm{i,app}}$ values to mutations in the flap is summarized in Figure S8. Compounds that were selected for detailed kinetic investigations by the steady-state initial rate method are identified with and asterisk (*). The top panel in Figure S8 shows absolute values of $K_{\mathrm{i,app}}$ results. The error bars in the upper panel depict the upper and lower bounds, respectively, of the 68% confidence interval, formally corresponding to one standard deviation, as determined by the profile-t method of Bates and Watts.[S28,S29] The error bars in the lower panel ($K_{\mathrm{i,app}}$ ratios) were computed by considering the lower bound of $K_{\mathrm{i,app}}$ for the wild-type enzyme

divided by the upper bound of $K_{\mathrm{i,app}}$ for the given mutant, and vice versa. Upon visual examination, the largest shifts in $K_{\mathrm{i,app}}$ values are seen in the L413A mutant. In contrast, the results for the L413V mutant show essentially negligible changes in $K_{\mathrm{i,app}}$ (within a factor of 2 in either direction for all 23 inhibitors). The L413F mutant represents an intermediate case.

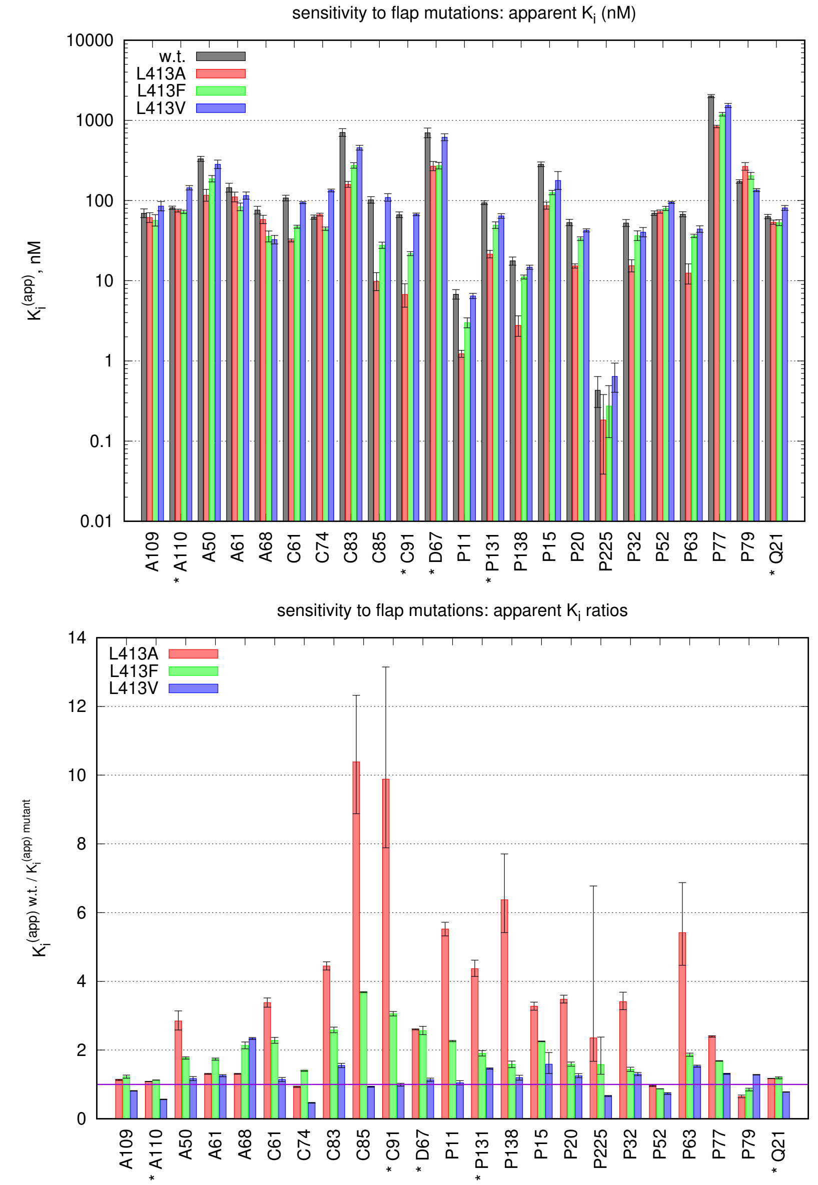

Figure S8: **Sensitivity of $K_{\mathrm{i,app}}$ values to flap mutations.** Asterisks denote inhibitors characterized in detail. For details see text.

7 Simulation of effects of mutations on equilibrium between $[\mathrm{E\text{-}XMP^*}]_{\mathrm{open}}$ and $[\mathrm{E\text{-}XMP^*}]_{\mathrm{closed}}$

We hypothesized that the mutations of Leu413 would affect the equilibrium between open and closed conformation (K_c) of E-XMP* to the same extent that the analogous mutations destabilize folded proteins. Therefore we used the online application SAAFEC-SEQ to calculate the effects of the Leu413 mutations on the stability of $[\mathrm{E\text{-}XMP^*}]_{\mathrm{closed}}$.[S34] Substitution of Leu413 with Ala, Val, and Phe destabilized $[\mathrm{E\text{-}XMP^*}]_{\mathrm{closed}}$ by ΔΔG of 2.42, 1.63 and 1.71 kcal/mol, respectively, corresponding to changes in values of the K_c by factors of 60, 16, and 18 relative to wild-type enzyme.

$$\Delta G = -RT\ln(K) \tag{S13}$$

The inhibitors bind to $[\mathrm{E\text{-}XMP^*}]_{\mathrm{open}}$, so we are interested in changes to the fraction of $[\mathrm{E\text{-}XMP^*}]_{\mathrm{open}}$ rather than the value of K_c per se. Therefore we simulated the fraction of $[\mathrm{E\text{-}XMP^*}]_{\mathrm{open}}$ for varying values of K_c of wild-type enzyme (Figure S9). If *Ba*IMPDH prefers open conformation ($K_c < 1$), then the mutations will have little effect on the fraction of $[\mathrm{E\text{-}XMP^*}]_{\mathrm{open}}$ even when the value of K_c decreases by a factor of 16 or more.

L413V mutation has little effect on the potency of the inhibitors, with an average potency change ($K_{i,app}(\mathrm{WT})/K_{i,app}(\mathrm{mutant})$) of 1.2. These results suggest that *Ba*IMPDH prefers open conformation and the K_c for the wild-type enzyme is no more than 0.2. Further, we suggest that a change in potency of 1.2 results from destabilization of $[\mathrm{E\text{-}XMP^*}]_{\mathrm{closed}}$, and changes in $K_{i,app}$ beyond 1.2 can be attributed to inhibitor-specific effects of the mutation.

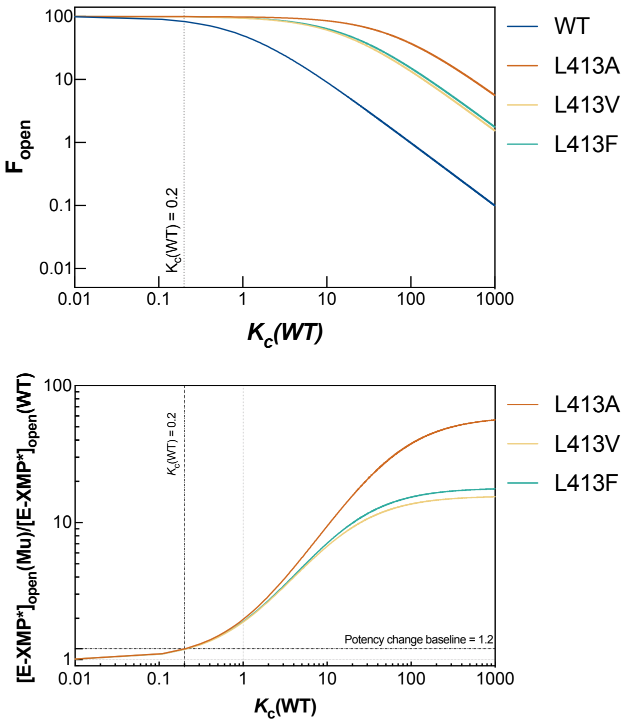

Figure S9: Upper: the fraction of E-XMP* in open conformation for both wild-type and mutant enzymes under different K_c(WT) are plotted. Lower: A plot on [E-XMP*]$_\mathrm{open}$(Mu)/[E-XMP*]$_\mathrm{open}$(WT) vs K_c(WT) to show the fold change of [E-XMP*]$_\mathrm{open}$ in the mutant enzymes. The K_c(WT) of 0.2 suggests that potency change ($K_\mathrm{i,app}$(WT)/$K_\mathrm{i,app}$(mutant)) at 1.2 results from destabilization of [E-XMP*]$_\mathrm{closed}$. See text for more details.

8 Multiple inhibitor experiment

A multiple inhibitor experiment was performed to evaluate if the open conformation is favored in *Ba*IMPDH. The reactions were performed with 20 nM *Ba*IMPDH, 0.6 mM IMP and 1.8 mM NAD^+. The production of NADH at 12 varying tiazofurin or ADP concentrations (0 to 5 mM) was monitored by UV/Vis absorbance at 340 nm for 15 minutes. The results are listed in the following table. The results show that the presence of ADP does not increase the potency of tiazofurin, which suggests the open conformation predominates in *Ba*IMPDH.

Table S10: The results of multiple inhibitor experiments.

	$K_{\mathrm{i,app}}$	lo	hi
Tiazofurin	0.62	0.57	0.67
Tiazofurin + 4 mM ADP	0.55	0.51	0.58
ADP	6.2	5.9	6.7
ADP + 2 mM Tiazofurin	3.7	3.3	4

9 Stopped-flow transient kinetics

9.1 Experimental data

9.1.1 Data reduction

Each raw kinetic trace consisted of 10,000 data points spanning from $t = 0$ to $t = 2.5$ s (0.25 m increment). Preliminary data reduction and adjustment procedure followed a four-step data adjustment protocol, as was described previously.[S15] First, data points collected at $t < 4$ ms, corresponding to the dead time of a stopped-flow instrument, were excluded from analysis. Second, data points corresponding to $t > 0.5$ s were excluded from analysis for kinetic traces that did not involve the inhibitor **A110**. Third, the remaining time-series were reduced to $n = 25$ time points spaced exponentially. See the discussion in ref.[S15] (*Supporting Information*), which documents the benefits of this particular data reduction protocol in the parameter estimation from stopped-flow experiments involving IMPDH. Finally, each individual kinetic trace was shifted on the vertical (absorbance) axis such that individual traces comprising a global combinatorial data set (see below) would not overlap in a graphical display. Importantly, such arbitrary vertical shift does not influence the results of the global regression analysis in particular because the individual offsets on the signal axis are treated as adjustable model parameters.

9.1.2 Combinatorial replication

In this work, we followed the combinatorial replication protocol explained in detail in ref.[S15] (*Supporting Information*). In particular, multiple sets of seven kinetic traces were collected at NAD^+ final concentrations equal to $[NAD]^+ = 0.25$, 0.5, 1.0, 2.0, 4.0, 6.0 and 8.0 mM. Each of the seven traces was obtained as the average of twelve independent injections. There were three types of such experiments designated "N", "H", and "I" as specified in Table S11. The concentration of IMP was equal to [IMP] = 1.0 mM in each instance.

Thus, each dataset for global[S30] regression analysis consisted of 21 kinetic traces, namely,

Table S11: Three types of stopped-flow experiments performed in this study. For details see text.

Experiment type	$[NAD^+]$, mM	[NADH], μM	[A110], μM	replicates WT	replicates L413A
"N"	0.25, 0.5, ..., 6, 8	0	0	2	2
"H"	0.25, 0.5, ..., 6, 8	60	0	2	3
"I"	0.25, 0.5, ..., 6, 8	0	6	3	3

seven traces where $[NAD^+]$ was varied in the absence of any additional component; seven traces where $[NAD^+]$ was varied in the presence of added NADH (product inhibition); and finally seven traces where $[NAD^+]$ was varied in the presence of the inhibitor **A110**.

Given the number of available replicates of each particular experiment type, in the case of the wild-type enzyme there exist $3 \times 2 \times 2 = 12$ ways in which a group of seven stopped-flow traces can be systematically drawn from the combined data library. Similarly, in the case of the L413A mutant, there exist $2 \times 3 \times 3 = 18$ combinatorial replicates. All microscopic rate constants reported in this document, as well as the lower and upper limits of their respective confidence intervals, are geometric means and geometric standard deviations determined from such (combinatorially) replicated global data sets.

9.2 Theoretical model

9.2.1 Postulated stopped-flow kinetic mechanism

The postulated kinetic model for the stopped-flow experiments is shown in Figure S10, were the symbols A, B, P, Q, I represent IMP, NAD^+, NADH, XMP, and **A110**, respectively.

Note that the free enzyme E is absent from this mechanistic scheme, because the concentration of IMP was purposely chosen such that [IMP] >> $K_m^{(IMP)}$. In all experiments, the enzyme and IMP were preincubated in syringe A. Thus, the enzyme is always fully saturated with IMP at the start of the transient kinetic recording. Consequently the apparent rate constant k'_5 in Figure S10 is an amalgamation of three separate microscopic processes: (1) irreversible hydrolysis of the covalent intermediate E-XMP* (abbreviated as EQ); (2)

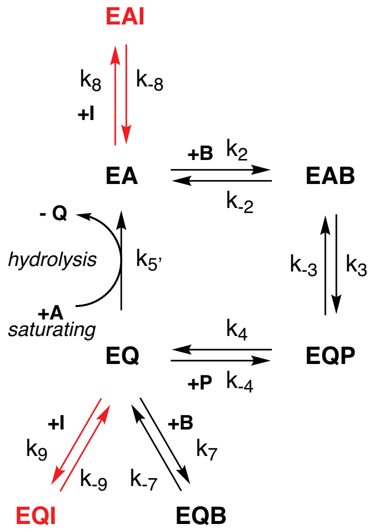

Figure S10: Minimal stopped-flow kinetic mechanism for the the inhibition *Ba*IMPDH by **A110**. E: IMPDH, A: IMP, B: NAD^+, Q: XMP, P: NADH, I: **A110**. Among enzyme complexes, EQ stands for E-XMP*. For details see text.

reversible release of XMP as the final product; and (3) reversible binding of IMP to the liberated free enzyme.

9.2.2 Regression equation and the associated ODE system

The nonlinear least-squares regression model for individual kinetic traces is defined by Eqn (S14), where A is the absorbance (in mOD, 10^{-3} dimensionless absorbance units) at 340 nm at reaction time $t > 0.004$ sec; A_0 is the adjustable baseline offset at $t_0 = 0.004$ sec, essentially a property of the instrument; $r_\mathrm{P} = 6.22\ \mathrm{mOD}/\mu\mathrm{M}$ is the molar response coefficient of NADH[2]; [P] is the concentration of NADH at time t; and [EQP] is the concentration of the ternary complex E-XMP*·NADH.

$$A = A_0 + r_\mathrm{P}\left([\mathrm{P}] + [\mathrm{EQP}]\right) \tag{S14}$$

The species concentrations [P] and [EQP] at time t were computed from their initial

[2]Note that the molar response coefficient is *not* identical to the extinction coefficient of NADH, but rather it is the extinction coefficient multiplied by the pathlength, in dimensionless mOD units (10^{-3} dimensionless absorbance units) per micromole/liter of NADH generated in the reaction.

concentrations at time zero, by numerically solving an initial-value problem defined by the ODE system in Eqns (S15)–(S25).

$$\frac{\mathrm{d[EA]}}{\mathrm{d}t} = -k_2[\mathrm{EA}][\mathrm{B}] + k_{-2}[\mathrm{EAB}] + k_5[\mathrm{EQ}] - k_8[\mathrm{EA}][\mathrm{I}] + k_{-8}[\mathrm{EAI}] \tag{S15}$$

$$\frac{\mathrm{d[B]}}{\mathrm{d}t} = -k_2[\mathrm{EA}][\mathrm{B}] + k_{-2}[\mathrm{EAB}] - k_7[\mathrm{EQ}][\mathrm{B}] + k_{-7}[\mathrm{EQB}] \tag{S16}$$

$$\frac{\mathrm{d[EAB]}}{\mathrm{d}t} = +k_2[\mathrm{EA}][\mathrm{B}] - k_{-2}[\mathrm{EAB}] - k_3[\mathrm{EAB}] + k_{-3}[\mathrm{EQP}] \tag{S17}$$

$$\frac{\mathrm{d[EQP]}}{\mathrm{d}t} = +k_3[\mathrm{EAB}] - k_{-3}[\mathrm{EQP}] - k_4[\mathrm{EQP}] + k_{-4}[\mathrm{EQ}][\mathrm{P}] \tag{S18}$$

$$\frac{\mathrm{d[EQ]}}{\mathrm{d}t} = +k_4[\mathrm{EQP}] - k_{-4}[\mathrm{EQ}][\mathrm{P}] - k_5[\mathrm{EQ}] - k_7[\mathrm{EQ}][\mathrm{B}] + k_{-7}[\mathrm{EQB}] - k_9[\mathrm{EQ}][\mathrm{I}] + k_{-9}[\mathrm{EQI}] \tag{S19}$$

$$\frac{\mathrm{d[P]}}{\mathrm{d}t} = +k_4[\mathrm{EQP}] - k_{-4}[\mathrm{EQ}][\mathrm{P}] \tag{S20}$$

$$\frac{\mathrm{d[P]}}{\mathrm{d}t} = +k_5[\mathrm{EP}] \tag{S21}$$

$$\frac{\mathrm{d[EQB]}}{\mathrm{d}t} = +k_7[\mathrm{EQ}][\mathrm{B}] - k_{-7}[\mathrm{EQB}] \tag{S22}$$

$$\frac{\mathrm{d[I]}}{\mathrm{d}t} = -k_8[\mathrm{EA}][\mathrm{I}] + k_{-8}[\mathrm{EAI}] - k_9[\mathrm{EQ}][\mathrm{I}] + k_{-9}[\mathrm{EQI}] \tag{S23}$$

$$\frac{\mathrm{d[EAI]}}{\mathrm{d}t} = +k_8[\mathrm{EA}][\mathrm{I}] - k_{-8}[\mathrm{EAI}] \tag{S24}$$

$$\frac{\mathrm{d[EQI]}}{\mathrm{d}t} = +k_9[\mathrm{EQ}][\mathrm{I}] - k_{-9}[\mathrm{EQI}] \tag{S25}$$

The model equations listed above were automatically derived by the software package DynaFit[S14] by using the coding listed below. The [mechanism] section is used within DynaFit to derive the ODE system Eqns (S15)–(S25). The [responses] section is used internally to derive the regression Eqn (S14).

```
[mechanism]

   E.A + B <==> E.A.B      :   k2    k-2

   E.A.B <==> E.Q.P        :   k3    k-3
```

```
   E.Q.P <==> E.Q + P      :   k4    k-4
   E.Q ---> E.A + Q        :   k5
   E.Q + B <==> E.Q.B      :   k7    k-7
   E.A + I <==> E.A.I      :   k8    k-8
   E.Q + I <==> E.Q.I      :   k9    k-9
...
[responses]
   P = 6.22
   E.Q.P = 1 * P
```

The system of differential equations was solved numerically by using the LSODE algorithm devised by Hindmarsh *et al.*[S35,S36] The relative truncation error tolerance in this predictor-corrector method was $\epsilon_{\mathrm{R}} = 10^{-8}$; the absolute error tolerance was $\epsilon_{\mathrm{A}} = 10^{-15}$.

9.2.3 Global vs. local classification of model parameters

Each global[S30] data set, analyzed as a single unit, consisted of 21 kinetic traces of three different types as was described in section *Experimental data.* The nonlinear least-squares regression model for each global set of $7 \times 25 = 525$ data points contained 36 adjustable parameters, some of which were global, i.e., associated with all 21 data traces; and some of which were *local*, i.e., associated with individual traces. This breakdown of regression parameters into two categories is explained in Table S12.

Table S12: Organization of regression parameters into categories. For details see text.

parameter no.	*parameter*	*type*
1–13	$k_2 - k_{-9}$	global
14	initial $[\mathrm{EA}]_0$	global
15–35	offset A_0	local

9.2.4 Empirical confidence intervals

Empirical confidence intervals for microscopic rate constants appearing in Eqns (S15)–(S25) were determined by the heuristic method advocated by Johnson,[S16–S18] at $\Delta SSQ = 5\%$. The confidence interval search otherwise followed the profile-t algorithm proposed by Bates and Watts.[S28,S29,S37] Unlike in the least-squares fit proper, all locally optimized parameters (namely, the 21 signal offset values for individual progress curves) were held as fixed constants during the confidence-interval search.

9.3 Representative example

9.3.1 Typical experimental data for the L413A mutant

The tables shown immediately below list typical absorbance values (scaled by the factor of 1000 and adjusted as described in section *Experimental data:Data reduction* from three sets of stopped-flow experiments involving the L413A mutant enzyme.

Table S13: **In the absence of NADH and A110**: Replicate 1 / 2

	[NAD$^+$], μM						
t (s)	250	500	1000	2000	4000	6000	8000
0.00400	0.000	1.000	2.000	4.000	6.000	10.000	16.000
0.00500	-0.046	1.029	2.127	3.966	6.016	10.377	15.889
0.00600	0.042	1.161	2.147	4.279	6.180	10.629	15.977
0.00750	0.059	1.446	2.362	4.405	6.761	11.202	16.981
0.00900	-0.063	1.252	2.390	4.722	7.113	11.468	17.483
0.01100	0.030	1.499	2.554	4.880	7.685	12.158	18.305
0.01350	0.061	1.513	2.708	5.328	8.364	12.979	19.312
0.01650	0.177	1.654	3.105	5.900	9.279	14.119	20.553
0.02025	0.394	1.978	3.415	6.540	10.085	15.192	21.592
0.02450	0.497	2.117	3.856	7.211	11.105	16.374	22.927
0.03000	0.622	2.334	4.274	7.770	12.095	17.444	24.364
0.03675	0.744	2.613	4.736	8.595	13.283	18.917	25.706
0.04475	1.023	2.930	5.329	9.741	14.439	20.036	26.443
0.05475	1.176	3.489	6.172	10.879	15.618	21.233	27.463
0.06700	1.303	3.837	6.945	11.927	16.870	22.654	28.086
0.08200	1.896	4.642	8.291	13.560	18.411	24.049	29.236
0.10025	2.207	5.230	9.498	14.974	19.904	25.280	30.394
0.12250	2.759	6.221	10.783	16.622	21.456	26.544	31.492
0.14975	3.225	7.115	12.193	18.239	22.860	27.867	32.433
0.18300	3.865	8.304	13.767	20.138	24.427	29.156	33.521
0.22375	4.674	9.709	15.630	22.178	26.210	30.836	34.792
0.27350	5.642	11.295	17.592	24.500	28.303	32.548	36.308
0.33450	6.838	13.092	20.153	27.195	30.705	34.684	38.458
0.40900	8.218	15.193	23.101	30.095	33.421	37.195	40.888
0.50000	9.809	17.859	26.174	33.665	36.819	40.437	43.518

Table S14: **In the presence of NADH**: Replicate 1 / 3

	[NAD+], μM						
t (s)	250	500	1000	2000	4000	6000	8000
0.00400	0.000	1.000	2.000	4.000	6.000	9.000	12.000
0.00500	0.057	1.099	2.012	3.492	5.984	8.588	12.269
0.00600	-0.163	1.033	1.870	3.318	5.906	8.401	12.235
0.00750	0.227	1.372	2.033	3.942	6.437	9.367	13.020
0.00900	0.201	1.319	2.132	4.405	6.919	10.178	13.602
0.01100	0.623	1.145	2.321	4.895	7.543	10.829	14.211
0.01350	0.425	1.265	2.227	5.014	8.016	11.479	14.949
0.01650	0.484	1.345	2.658	5.456	8.654	12.295	15.646
0.02025	0.644	1.673	3.194	6.436	9.653	13.447	16.837
0.02450	0.491	1.808	3.285	6.721	10.338	14.207	17.902
0.03000	1.019	2.214	3.690	7.540	11.365	15.752	19.491
0.03675	1.194	2.293	4.124	8.300	12.380	16.705	20.648
0.04475	0.840	2.655	4.565	8.428	13.029	17.227	21.510
0.05475	0.839	2.892	5.149	8.910	14.079	17.869	22.417
0.06700	0.865	2.989	5.527	9.965	15.035	18.615	23.368
0.08200	1.194	3.640	6.538	11.156	16.488	20.020	24.568
0.10025	1.379	4.131	7.393	12.497	17.900	21.215	25.580
0.12250	1.885	4.946	8.639	14.250	19.305	22.350	26.718
0.14975	2.324	5.564	9.659	15.666	20.510	23.589	27.503
0.18300	3.027	6.537	11.111	17.242	21.880	24.607	28.479
0.22375	3.781	7.828	12.836	18.915	23.432	25.887	29.748
0.27350	4.736	9.325	14.419	20.744	24.966	27.478	31.280
0.33450	5.853	10.905	16.441	22.900	26.934	29.306	33.043
0.40900	6.777	12.452	18.500	25.250	29.081	31.362	34.926
0.50000	8.537	14.587	21.331	28.204	31.894	33.808	37.347

Table S15: **In the presence of A110**: Replicate 1 / 3

	[NAD$^+$], μM						
t (s)	250	500	1000	2000	4000	6000	8000
0.00400	0.000	1.000	2.000	4.000	6.000	9.000	12.000
0.00525	0.071	1.004	1.958	4.033	6.013	9.087	12.107
0.00700	0.076	1.207	1.935	4.005	6.328	9.271	12.543
0.00900	0.114	1.272	2.216	4.524	6.782	9.793	13.303
0.01175	0.078	1.336	2.365	4.682	7.110	10.516	13.789
0.01550	0.150	1.355	2.429	5.012	7.763	11.228	14.934
0.02025	0.329	1.525	2.734	5.435	8.430	12.226	15.650
0.02625	0.431	1.733	3.096	5.938	9.244	13.017	16.808
0.03425	0.295	1.798	3.225	6.377	9.909	13.954	17.837
0.04475	0.587	2.020	3.578	6.734	10.466	14.616	18.563
0.05850	0.747	2.267	4.059	7.490	11.162	15.164	19.065
0.07650	0.891	2.280	4.506	8.346	11.982	15.746	19.764
0.10025	1.132	2.612	5.135	9.355	13.425	16.939	21.125
0.13100	1.469	3.061	6.023	10.817	15.045	18.678	22.716
0.17100	1.705	3.567	6.885	12.148	16.599	20.236	24.122
0.22375	2.074	4.399	8.110	13.733	18.180	21.447	25.094
0.29250	2.794	5.628	9.750	15.375	19.851	22.819	26.308
0.38250	3.577	6.552	11.391	17.301	21.343	23.987	27.113
0.50025	4.356	8.129	13.233	18.970	22.496	24.742	27.761
0.65400	5.502	9.630	14.993	20.463	23.304	25.285	28.178
0.85500	6.789	11.508	17.015	21.523	23.964	25.892	28.803
1.11825	8.268	13.538	18.757	22.523	24.520	26.316	29.299
1.46225	10.118	15.259	19.908	23.503	25.113	27.035	29.884
1.91200	12.053	17.389	21.417	24.170	25.786	27.809	30.787
2.50000	14.143	19.042	22.326	25.437	26.826	28.609	31.724

9.3.2 DynaFit script file listing

The coding below contains the input text for the software DynaFit[S14] utilized to perform the global[S30] fit of combined progress curves listed in the previous section. For details, see the DynaFit *Scripting Manual* distributed with the software.[3]

```
[task]
   task = fit
   data = progress
[mechanism]
   EA + B <==> EAB        :   k2    k-2
   EAB <==> EQP           :   k3    k-3
   EQP <==> EQ + P        :   k4    k-4
   EQ ---> EA + Q         :   k5
   EQ + B <==> EQB        :   k7    k-7
   EA + I <==> EAI        :   k8    k-8
   EQ + I <==> EQI        :   k9    k-9
[constants]
   k2    = 0.01 ??   ,   k-2   = 10 ??
   k3    = 100 ??    ,   k-3   = 10 ??
   k4    = 10000 ??  ,   k-4   = 10 ??
   k5    = 10 ??
   k7    = 0.001 ??  ,   k-7   = 10 ??
   k8    = 10 ??     ,   k-8   = 10 ??
   k9    = 1 ??      ,   k-9   = 0.1 ??
[concentrations]
   EA = 2.5 ?
```

[3]DynaFit is available free of charge to all academic and non-profit institutions as a download from http://www.biokin.com.

```
[responses]
   P = 6.22
   EQP = 1 * P
[data]
   directory ./project/IMPDH/_FINAL/SF-f/L413A/data-f
   monitor   EA, EAB, EQP, EQ, EQB, EAI, EQI
   plot      logarithmic
graph [NADH] = 0, [A110] = 0
sheet L413A-N1-f.csv
   column 2 | offset auto ? | conc B = 250 | label 0.25
   column 3 | offset auto ? | conc B = 500 | label 0.5
   column 4 | offset auto ? | conc B = 1000 | label 1
   column 5 | offset auto ? | conc B = 2000 | label 2
   column 6 | offset auto ? | conc B = 4000 | label 4
   column 7 | offset auto ? | conc B = 6000 | label 6
   column 8 | offset auto ? | conc B = 8000 | label 8 mM
graph [NADH] = 60 {/Symbol m}M, [A110] = 0
sheet L413A-H1-f.csv
   column 2 | offset -373 ? | conc P = 60, B = 250 | label 0.25
   column 3 | offset -372 ? | conc P = 60, B = 500 | label 0.5
   column 4 | offset -371 ? | conc P = 60, B = 1000 | label 1
   column 5 | offset -370 ? | conc P = 60, B = 2000 | label 2
   column 6 | offset -368 ? | conc P = 60, B = 4000 | label 4
   column 7 | offset -365 ? | conc P = 60, B = 6000 | label 6
   column 8 | offset -362 ? | conc P = 60, B = 8000 | label 8 mM
graph [NADH] = 0, [A110] = 6 {/Symbol m}M
sheet L413A-I1-f.csv
```

```
   column 2 | offset auto ? | conc I = 6, B = 250 | label 0.25
   column 3 | offset auto ? | conc I = 6, B = 500 | label 0.5
   column 4 | offset auto ? | conc I = 6, B = 1000 | label 1
   column 5 | offset auto ? | conc I = 6, B = 2000 | label 2
   column 6 | offset auto ? | conc I = 6, B = 4000 | label 4
   column 7 | offset auto ? | conc I = 6, B = 6000 | label 6
   column 8 | offset auto ? | conc I = 6, B = 8000 | label 8 mM
[output]
   directory ./project/IMPDH/SF-f/L413A/fit-reps/output/fit-N1H1I1
[settings]
{Filter}
   TimeFirstMesh = 0.003
{ConfidenceIntervals}
   OnlyConstants = y
   SquaresIncreasePercent = 5
   ParameterMaxRatio = 10
{Output}
   XAxisLabel = t, s
   YAxisLabel = 1000 {/Symbol D}A_{340}
[end]
```

9.3.3 Results of fit

The best-fit values of adjustable model parameters for the representative example data set are listed in the table immediately below. The columns labeled "low" and "high" are the lower and upper bounds, respectively, of the empirical confidence intervals as defined above section.

Table S16: The best-fit values of adjustable model parameters for the representative example data set

#	par/set	initial	final ± std.err.	cv,%	low	high	note
1	k_2	0.01	0.00868 ± 0.00028	6.5	0.008	0.00978	
2	k_{-2}	10	9.6 ± 2.2	46.4	3.4	21	
3	k_3	100	144 ± 18	25.3	120	>> 29100	
4	k_{-3}	10	216 ± 75	70.3	78.4	5750	
5	k_4	10000	100000 ± 5.1e+006	>> 100	260	>> 7.6e+007	
6	k_{-4}	10	230 ± 12000	>> 100	0.4	>> 170000	
7	k_5	10	6.42 ± 0.19	6.0	5.83	7.13	
8	k_7	0.001	0.00104 ± 0.00011	21.4	0.000647	0.00163	
9	k_{-7}	10	4.51 ± 0.73	32.8	1.89	8.04	
10	k_8	10	11.65 ± 0.98	17.0	9.049	15.44	
11	k_{-8}	10	28.3 ± 2.4	17.2	21.5	38.6	
12	k_9	1	2.93 ± 0.14	9.7	2.45	3.49	
13	k_{-9}	0.1	0.23 ± 0.029	25.5	0.117	0.367	
14	[EA]	2.5	2.032 ± 0.023	2.3			
15	offset / 1	0	-0.068 ± 0.063	> 100			
16	offset / 2	1	1.166 ± 0.067	11.6			
17	offset / 3	2	2.125 ± 0.071	6.8			
18	offset / 4	4	3.941 ± 0.078	4.0			
19	offset / 5	6	5.526 ± 0.098	3.6			
20	offset / 6	10	9.54 ± 0.11	2.3			
21	offset / 7	16	14.58 ± 0.13	1.8			
22	offset / 8	-373	-373.191 ± 0.063	0.0			
23	offset / 9	-372	-372.254 ± 0.066	0.0			
24	offset / 10	-371	-371.524 ± 0.07	0.0			
25	offset / 11	-370	-369.605 ± 0.077	0.0			
26	offset / 12	-368	-367.733 ± 0.094	0.1			
27	offset / 13	-365	-365.39 ± 0.1	0.1			
28	offset / 14	-362	-362.01 ± 0.11	0.1			
29	offset / 15	0	0.257 ± 0.065	51.2			
30	offset / 16	1	1.035 ± 0.068	13.3			
31	offset / 17	2	1.931 ± 0.071	7.4			
32	offset / 18	4	3.799 ± 0.076	4.0			
33	offset / 19	6	5.203 ± 0.085	3.3			
34	offset / 20	9	7.43 ± 0.091	2.5			
35	offset / 21	12	10.373 ± 0.097	1.9			

N.B.: Empirical coefficients of variations (CV) assuming 10-fold ratio of data points over optimized model parameters.

The results of fit are illustrated graphically in Figure S11.

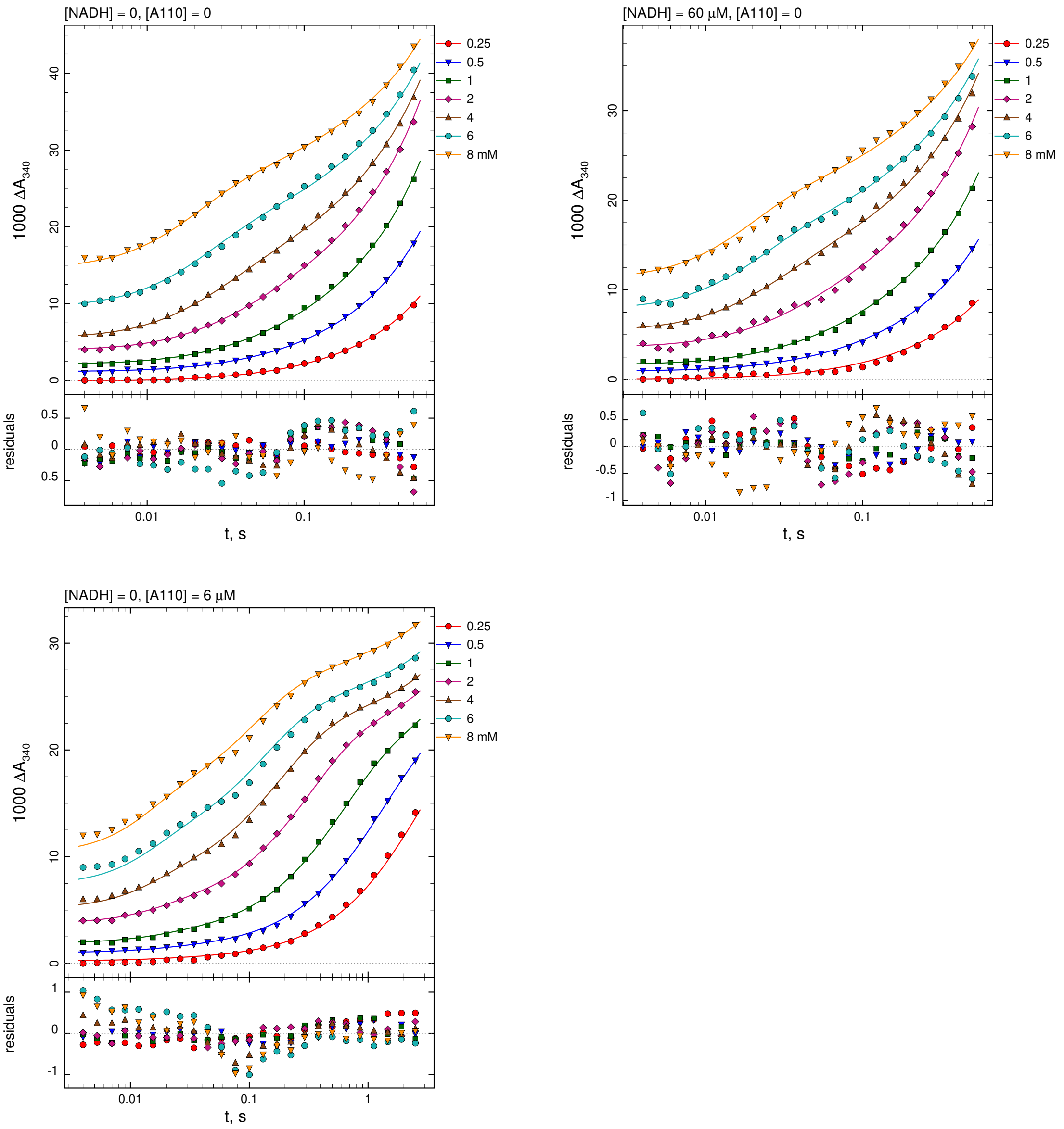

Figure S11: Data vs. model overlay from the least-squares fit of the example data set. For details see text.

9.4 Summary of results

9.4.1 Individual combinatorial replicates

Best-fit values and/or lower limits for microscopic rate constants determined in the stopped-flow experiments are shown in Figure S12 for individual combinatorial replicates. The micro-

scopic rate constants k_4 and k_{-4} that characterize the dissociation and rebinding of NADH, respectively, were essentially undefined by the experimental data, in that (a) individual combinatorial replicates produced vastly different best-fit values; and (b) the upper limits of the confidence intervals for either rate constant were approaching positive infinity. In contrast, the lower limits of both k_4 and k_{-4} were very well reproduced (see Figure S12).

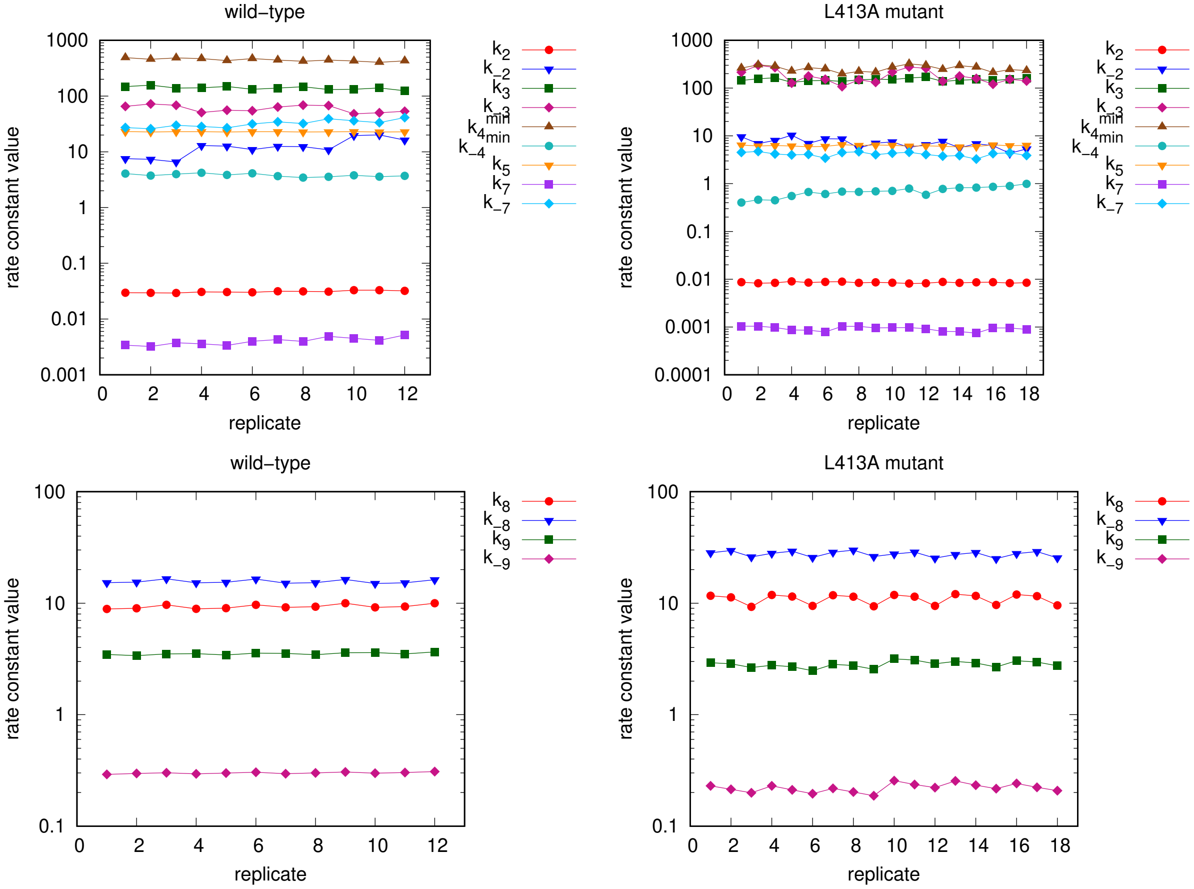

Figure S12: Best-fit values and/or lower limits for microscopic rate constants determined in the stopped-flow experiments: Individual combinatorial replicates.

9.4.2 Geometric means and geometric standard deviations

Geometric means and geometric standard deviations from the combinatorial replicates are summarized in *Figure 7*, along with relevant ratios of certain rate constants that constitute the corresponding equilibrium dissociation constants. Note that the equilibrium dissociation constant for NADH (k_4/k_{-4}) is very well defined by the stopped-flow data, for both the

wild-type enzyme and the L413A mutant, despite the fact that the individually replicated values of k_4 and k_{-4} are not defined.

10 Steady-state initial rate kinetics

10.1 Experimental data

The experimental data for detailed mechanistic initial rate studies described in this section consisted of a multi-dimensional grid of initial rate measurements, where both substrates (IMP and NAD^+), both products (NADH and XMP), and the inhibitor (e.g., **A110**) were varied simultaneously and the composite data were analyzed by the global regression approach.[S30] Thus, each global multi-dimensional data set consisted of five independent variables.

10.2 Theoretical model

10.2.1 Postulated stopped-flow kinetic mechanism

The postulated kinetic model for the initial rate experiments described in this section is shown in Figure S13, were the symbols A, B, P, Q, I again represent IMP, NAD^+, NADH, XMP, and **A110**, respectively. Note that the free enzyme E is present explicitly, because the concentration of IMP was purposely chosen such that $[\text{IMP}] \approx K_\text{m}^{(\text{IMP})}$. Thus, unlike in the stopped-flow experiments discussed in the above section, the mole fraction of the free enzyme E cannot be neglected in the mass balance. Also note that the microscopic rate constant k_5 in Figure S13 now represents the hydrolytic step alone. Unlike in Figure S10, the reversible release of XMP as the final product is characterized by microscopic rate constants k_6 and k_{-6}, respectively. In its turn, the reversible binding of IMP to the liberated free enzyme is characterized by microscopic rate constants k_1 and k_{-1}.

10.2.2 Mathematical models

In this work we have utilized four different variations of the overall mechanistic scheme displayed in Figure S13. In the notation below, square brackets without subscript zero represent the steady-state concentrations, whereas square brackets with subscript zero represent total

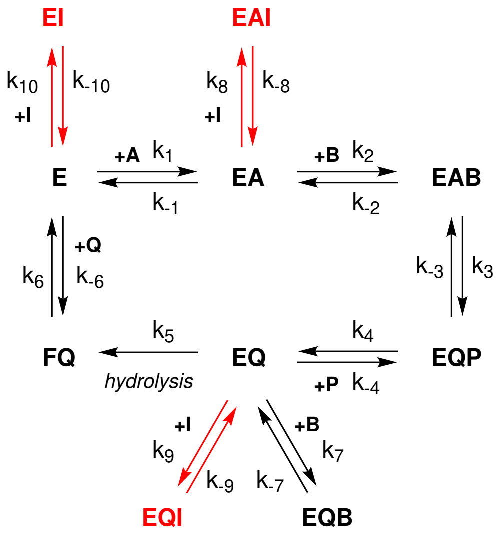

Figure S13: Initial rate kinetic mechanism for the the inhibition *Ba*IMPDH by **A110**. E, F: IMPDH, A: IMP, B: NAD^+, Q: XMP, P: NADH, I: **A110**. Among enzyme complexes, EQ stands for E-XMP*and FQ stands for E•XMP. For details see text.

or analytic concentrations. In each case, the initial rate equation, the nonlinear algebraic system, and the associated Jacobian matrix represent the general numerical for steady-state enzyme kinetics described elsewhere.[S19]

10.2.2.1 Model "S": Substrate catalysis

Mechanism

$$\mathbf{A + B} \;\rightarrow\; \mathbf{P + Q}$$

$$\begin{array}{rcll} & & & \rightarrow \quad \leftarrow \\ \mathrm{E + A} & \rightleftharpoons & \mathrm{EA} & : \; k_1 \quad k_{-1} \\ \mathrm{EA + B} & \rightleftharpoons & \mathrm{EAB} & : \; k_2 \quad k_{-2} \\ \mathrm{EAB} & \rightleftharpoons & \mathrm{EQP} & : \; k_3 \quad k_{-3} \\ \mathrm{EQP} & \rightleftharpoons & \mathrm{EQ + P} & : \; k_4 \quad k_{-4} \\ \mathrm{EQ} & \rightarrow & \mathrm{FQ} & : \; k_5 \\ \mathrm{FQ} & \rightleftharpoons & \mathrm{E + Q} & : \; k_6 \quad k_{-6} \\ \mathrm{B + EQ} & \rightleftharpoons & \mathrm{EQB} & : \; k_7 \quad k_{-7} \end{array}$$

Initial rate equation

$$v = \mathrm{d[P]}/\mathrm{d}t \;=\; k_4[\mathrm{EQP}] - k_{-4}[\mathrm{EQ}][\mathrm{P}] \tag{S26}$$

Nonlinear algebraic system

$$\textsc{e} : 0 \;=\; [\mathrm{E}] + [\mathrm{EA}] + [\mathrm{EAB}] + [\mathrm{EQP}] + [\mathrm{EQ}] + [\mathrm{FQ}] + [\mathrm{EQB}] - [\mathrm{E}]_0 \tag{S27}$$

$$\textsc{ea} : 0 \;=\; k_1[\mathrm{E}][\mathrm{A}] - k_{-1}[\mathrm{EA}] - k_2[\mathrm{EA}][\mathrm{B}] + k_{-2}[\mathrm{EAB}] \tag{S28}$$

$$\textsc{eab} : 0 \;=\; k_2[\mathrm{EA}][\mathrm{B}] - k_{-2}[\mathrm{EAB}] - k_3[\mathrm{EAB}] + k_{-3}[\mathrm{EQP}] \tag{S29}$$

$$\textsc{eqp} : 0 \;=\; k_3[\mathrm{EAB}] - k_{-3}[\mathrm{EQP}] - k_4[\mathrm{EQP}] + k_{-4}[\mathrm{EQ}][\mathrm{P}] \tag{S30}$$

$$\textsc{eq} : 0 \;=\; k_4[\mathrm{EQP}] - k_{-4}[\mathrm{EQ}][\mathrm{P}] - k_5[\mathrm{EQ}] - k_7[\mathrm{B}][\mathrm{EQ}] + k_{-7}[\mathrm{EQB}] \tag{S31}$$

$$\textsc{fq} : 0 \;=\; k_5[\mathrm{EQ}] - k_6[\mathrm{FQ}] + k_{-6}[\mathrm{E}][\mathrm{Q}] \tag{S32}$$

$$\textsc{eqb} : 0 \;=\; k_7[\mathrm{B}][\mathrm{EQ}] - k_{-7}[\mathrm{EQB}] \tag{S33}$$

Jacobian matrix:

$$\mathbf{J} = \begin{array}{c} \\ \textsc{e} \\ \textsc{ea} \\ \textsc{eab} \\ \textsc{eqp} \\ \textsc{eq} \\ \textsc{fq} \\ \textsc{eqb} \end{array} \begin{array}{c} \begin{array}{ccccccc} \textsc{e} & \textsc{ea} & \textsc{eab} & \textsc{eqp} & \textsc{eq} & \textsc{fq} & \textsc{eqb} \end{array} \\ \begin{pmatrix} 1 & 1 & 1 & 1 & 1 & 1 & 1 \\ k_1[\mathrm{A}] & J_{2,2} & k_{-2} & . & . & . & . \\ . & k_2[\mathrm{B}] & J_{3,3} & k_{-3} & . & . & . \\ . & . & k_3 & J_{4,4} & k_{-4}[\mathrm{P}] & . & . \\ . & . & . & k_4 & J_{5,5} & . & k_{-7} \\ k_{-6}[\mathrm{Q}] & . & . & . & k_5 & -k_6 & . \\ . & . & . & . & k_7[\mathrm{B}] & . & -k_{-7} \end{pmatrix} \end{array} \tag{S34}$$

$$J_{2,2} = -k_{-1} - k_2[\mathrm{B}] \quad \text{(S35)}$$

$$J_{3,3} = -k_{-2} - k_3 \quad \text{(S36)}$$

$$J_{4,4} = -k_{-3} - k_4 \quad \text{(S37)}$$

$$J_{5,5} = -k_{-4}[\mathrm{P}] - k_5 - k_7[\mathrm{B}] \quad \text{(S38)}$$

10.2.2.2 Model "A": Inhibitor binding to a single enzyme form (EQ)

Mechanism

$$\mathbf{A} + \mathbf{B} \rightarrow \mathbf{P} + \mathbf{Q}$$

				→	←
E + A	⇌	EA	:	k_1	k_{-1}
EA + B	⇌	EAB	:	k_2	k_{-2}
EAB	⇌	EQP	:	k_3	k_{-3}
EQP	⇌	EQ + P	:	k_4	k_{-4}
EQ	→	FQ	:	k_5	
FQ	⇌	E + Q	:	k_6	k_{-6}
B + EQ	⇌	EQB	:	k_7	k_{-7}
EQ + I	⇌	EQI	:	k_9	k_{-9}

Initial rate equation

$$v = \mathrm{d}[\mathrm{P}]/\mathrm{d}t = k_4[\mathrm{EQP}] - k_{-4}[\mathrm{EQ}][\mathrm{P}] \quad \text{(S39)}$$

Nonlinear algebraic system

$$\begin{aligned}
\text{E} : 0 &= [\text{E}] + [\text{EA}] + [\text{EAB}] + [\text{EQP}] + [\text{EQ}] + [\text{FQ}] + [\text{EQB}] + [\text{EQI}] - [\text{E}]_0 && \text{(S40)}\\
\text{I} : 0 &= [\text{I}] + [\text{EQI}] - [\text{I}] && \text{(S41)}\\
\text{EA} : 0 &= k_1[\text{E}][\text{A}] - k_{-1}[\text{EA}] - k_2[\text{EA}][\text{B}] + k_{-2}[\text{EAB}] && \text{(S42)}\\
\text{EAB} : 0 &= k_2[\text{EA}][\text{B}] - k_{-2}[\text{EAB}] - k_3[\text{EAB}] + k_{-3}[\text{EQP}] && \text{(S43)}\\
\text{EQP} : 0 &= k_3[\text{EAB}] - k_{-3}[\text{EQP}] - k_4[\text{EQP}] + k_{-4}[\text{EQ}][\text{P}] && \text{(S44)}\\
\text{EQ} : 0 &= k_4[\text{EQP}] - k_{-4}[\text{EQ}][\text{P}] - k_5[\text{EQ}] - k_7[\text{B}][\text{EQ}] + k_{-7}[\text{EQB}] && \text{(S45)}\\
&\quad -k_9[\text{EQ}][\text{I}] + k_{-9}[\text{EQI}] &&\\
\text{FQ} : 0 &= k_5[\text{EQ}] - k_6[\text{FQ}] + k_{-6}[\text{E}][\text{Q}] && \text{(S46)}\\
\text{EQB} : 0 &= k_7[\text{B}][\text{EQ}] - k_{-7}[\text{EQB}] && \text{(S47)}\\
\text{EQI} : 0 &= k_9[\text{EQ}][\text{I}] - k_{-9}[\text{EQI}] && \text{(S48)}
\end{aligned}$$

Jacobian matrix:

$$\mathbf{J} = \begin{array}{c} \\ \text{E} \\ \text{I} \\ \text{EA} \\ \text{EAB} \\ \text{EQP} \\ \text{EQ} \\ \text{FQ} \\ \text{EQB} \\ \text{EQI} \end{array} \begin{array}{c} \begin{array}{ccccccccc} \text{E} & \text{I} & \text{EA} & \text{EAB} & \text{EQP} & \text{EQ} & \text{FQ} & \text{EQB} & \text{EQI} \end{array} \\ \begin{pmatrix}
1 & . & 1 & 1 & 1 & 1 & 1 & 1 & 1 \\
. & 1 & . & . & . & . & . & . & 1 \\
k_1[\text{A}] & . & J_{3,3} & k_{-2} & . & . & . & . & . \\
. & . & k_2[\text{B}] & J_{4,4} & k_{-3} & . & . & . & . \\
. & . & . & k_3 & J_{5,5} & k_{-4}[\text{P}] & . & . & . \\
. & -k_9[\text{EQ}] & . & . & k_4 & J_{6,6} & . & k_{-7} & k_{-9} \\
k_{-6}[\text{Q}] & . & . & . & . & k_5 & -k_6 & . & . \\
. & . & . & . & . & k_7[\text{B}] & . & -k_{-7} & . \\
. & k_9[\text{EQ}] & . & . & . & k_9[\text{I}] & . & . & -k_{-9}
\end{pmatrix} \end{array} \qquad \text{(S49)}$$

$$J_{3,3} = -k_{-1} - k_2[\mathrm{B}] \quad \text{(S50)}$$

$$J_{4,4} = -k_{-2} - k_3 \quad \text{(S51)}$$

$$J_{5,5} = -k_{-3} - k_4 \quad \text{(S52)}$$

$$J_{6,6} = -k_{-4}[\mathrm{P}] - k_5 - k_7[\mathrm{B}] - k_9[\mathrm{I}] \quad \text{(S53)}$$

10.2.2.3 Model "B": Inhibitor binding simultaneously to two enzyme forms (EA, EQ)

Mechanism

$$\mathbf{A} + \mathbf{B} \rightarrow \mathbf{P} + \mathbf{Q}$$

$$\begin{array}{rcll} & & & \rightarrow \quad \leftarrow \\ \mathrm{E} + \mathrm{A} & \rightleftharpoons & \mathrm{EA} & : \; k_1 \quad k_{-1} \\ \mathrm{EA} + \mathrm{B} & \rightleftharpoons & \mathrm{EAB} & : \; k_2 \quad k_{-2} \\ \mathrm{EAB} & \rightleftharpoons & \mathrm{EQP} & : \; k_3 \quad k_{-3} \\ \mathrm{EQP} & \rightleftharpoons & \mathrm{EQ} + \mathrm{P} & : \; k_4 \quad k_{-4} \\ \mathrm{EQ} & \rightarrow & \mathrm{FQ} & : \; k_5 \\ \mathrm{FQ} & \rightleftharpoons & \mathrm{E} + \mathrm{Q} & : \; k_6 \quad k_{-6} \\ \mathrm{B} + \mathrm{EQ} & \rightleftharpoons & \mathrm{EQB} & : \; k_7 \quad k_{-7} \\ \mathrm{EA} + \mathrm{I} & \rightleftharpoons & \mathrm{EAI} & : \; k_8 \quad k_{-8} \\ \mathrm{EQ} + \mathrm{I} & \rightleftharpoons & \mathrm{EQI} & : \; k_9 \quad k_{-9} \end{array}$$

Initial rate equation

$$v = \mathrm{d}[\mathrm{P}]/\mathrm{d}t = k_4[\mathrm{EQP}] - k_{-4}[\mathrm{EQ}][\mathrm{P}] \quad \text{(S54)}$$

Nonlinear algebraic system

$$\begin{aligned}
\text{E}: 0 &= [\text{E}] + [\text{EA}] + [\text{EAB}] + [\text{EQP}] + [\text{EQ}] + [\text{FQ}] + [\text{EQB}] + [\text{EAI}] + [\text{EQI}] \\
&\quad -[\text{E}] && \text{(S55)}\\
\text{I}: 0 &= [\text{I}] + [\text{EAI}] + [\text{EQI}] - [\text{I}] && \text{(S56)}\\
\text{EA}: 0 &= k_1[\text{E}][\text{A}] - k_{-1}[\text{EA}] - k_2[\text{EA}][\text{B}] + k_{-2}[\text{EAB}] - k_8[\text{EA}][\text{I}] && \text{(S57)}\\
&\quad +k_{-8}[\text{EAI}] \\
\text{EAB}: 0 &= k_2[\text{EA}][\text{B}] - k_{-2}[\text{EAB}] - k_3[\text{EAB}] + k_{-3}[\text{EQP}] && \text{(S58)}\\
\text{EQP}: 0 &= k_3[\text{EAB}] - k_{-3}[\text{EQP}] - k_4[\text{EQP}] + k_{-4}[\text{EQ}][\text{P}] && \text{(S59)}\\
\text{EQ}: 0 &= k_4[\text{EQP}] - k_{-4}[\text{EQ}][\text{P}] - k_5[\text{EQ}] - k_7[\text{B}][\text{EQ}] + k_{-7}[\text{EQB}] && \text{(S60)}\\
&\quad -k_9[\text{EQ}][\text{I}] + k_{-9}[\text{EQI}] \\
\text{FQ}: 0 &= k_5[\text{EQ}] - k_6[\text{FQ}] + k_{-6}[\text{E}][\text{Q}] && \text{(S61)}\\
\text{EQB}: 0 &= k_7[\text{B}][\text{EQ}] - k_{-7}[\text{EQB}] && \text{(S62)}\\
\text{EAI}: 0 &= k_8[\text{EA}][\text{I}] - k_{-8}[\text{EAI}] && \text{(S63)}\\
\text{EQI}: 0 &= k_9[\text{EQ}][\text{I}] - k_{-9}[\text{EQI}] && \text{(S64)}
\end{aligned}$$

Jacobian matrix:

$$
\mathbf{J} = \begin{array}{c} \\ \text{E} \\ \text{I} \\ \text{EA} \\ \text{EAB} \\ \text{EQP} \\ \text{EQ} \\ \text{FQ} \\ \text{EQB} \\ \text{EAI} \\ \text{EQI} \end{array}
\begin{array}{c}
\begin{array}{cccccccccc} \text{E} & \text{I} & \text{EA} & \text{EAB} & \text{EQP} & \text{EQ} & \text{FQ} & \text{EQB} & \text{EAI} & \text{EQI} \end{array} \\
\begin{pmatrix}
1 & . & 1 & 1 & 1 & 1 & 1 & 1 & 1 & 1 \\
. & 1 & . & . & . & . & . & . & 1 & 1 \\
k_1[\text{A}] & -k_8[\text{EA}] & J_{3,3} & k_{-2} & . & . & . & . & k_{-8} & . \\
. & . & k_2[\text{B}] & J_{4,4} & k_{-3} & . & . & . & . & . \\
. & . & . & k_3 & J_{5,5} & k_{-4}[\text{P}] & . & . & . & . \\
. & -k_9[\text{EQ}] & . & . & k_4 & J_{6,6} & . & k_{-7} & . & k_{-9} \\
k_{-6}[\text{Q}] & . & . & . & . & k_5 & -k_6 & . & . & . \\
. & . & . & . & . & k_7[\text{B}] & . & -k_{-7} & . & . \\
. & k_8[\text{EA}] & k_8[\text{I}] & . & . & . & . & . & -k_{-8} & . \\
. & k_9[\text{EQ}] & . & . & . & k_9[\text{I}] & . & . & . & -k_{-9}
\end{pmatrix}
\end{array}
\quad \text{(S65)}
$$

$$J_{3,3} = -k_{-1} - k_2[\text{B}] - k_8[\text{I}] \quad \text{(S66)}$$

$$J_{4,4} = -k_{-2} - k_3 \quad \text{(S67)}$$

$$J_{5,5} = -k_{-3} - k_4 \quad \text{(S68)}$$

$$J_{6,6} = -k_{-4}[\text{P}] - k_5 - k_7[\text{B}] - k_9[\text{I}] \quad \text{(S69)}$$

10.2.2.4 Model "C": Inhibitor binding simultaneously to three enzyme forms (E, EA, EQ)

Mechanism

$$\mathbf{A} + \mathbf{B} \rightarrow \mathbf{P} + \mathbf{Q}$$

$$\begin{array}{rcllll}
 & & & & \rightarrow & \leftarrow \\
\mathrm{E} + \mathrm{A} & \rightleftharpoons & \mathrm{EA} & : & k_1 & k_{-1} \\
\mathrm{EA} + \mathrm{B} & \rightleftharpoons & \mathrm{EAB} & : & k_2 & k_{-2} \\
\mathrm{EAB} & \rightleftharpoons & \mathrm{EQP} & : & k_3 & k_{-3} \\
\mathrm{EQP} & \rightleftharpoons & \mathrm{EQ} + \mathrm{P} & : & k_4 & k_{-4} \\
\mathrm{EQ} & \rightarrow & \mathrm{FQ} & : & k_5 & \\
\mathrm{FQ} & \rightleftharpoons & \mathrm{E} + \mathrm{Q} & : & k_6 & k_{-6} \\
\mathrm{B} + \mathrm{EQ} & \rightleftharpoons & \mathrm{EQB} & : & k_7 & k_{-7} \\
\mathrm{EA} + \mathrm{I} & \rightleftharpoons & \mathrm{EAI} & : & k_8 & k_{-8} \\
\mathrm{EQ} + \mathrm{I} & \rightleftharpoons & \mathrm{EQI} & : & k_9 & k_{-9} \\
\mathrm{E} + \mathrm{I} & \rightleftharpoons & \mathrm{EI} & : & k_{10} & k_{-10}
\end{array}$$

Initial rate equation

$$v = \mathrm{d}[\mathrm{P}]/\mathrm{d}t \quad = \quad k_4[\mathrm{EQP}] - k_{-4}[\mathrm{EQ}][\mathrm{P}] \tag{S70}$$

Nonlinear algebraic system

$$\textsc{e}: 0 = [\mathrm{E}] + [\mathrm{EA}] + [\mathrm{EAB}] + [\mathrm{EQP}] + [\mathrm{EQ}] + [\mathrm{FQ}] + [\mathrm{EQB}] + [\mathrm{EAI}] + [\mathrm{EQI}] + [\mathrm{EI}] - [\mathrm{E}] \quad \text{(S71)}$$

$$\textsc{i}: 0 = [\mathrm{I}] + [\mathrm{EAI}] + [\mathrm{EQI}] + [\mathrm{EI}] - [\mathrm{I}] \quad \text{(S72)}$$

$$\textsc{ea}: 0 = k_1[\mathrm{E}][\mathrm{A}] - k_{-1}[\mathrm{EA}] - k_2[\mathrm{EA}][\mathrm{B}] + k_{-2}[\mathrm{EAB}] - k_8[\mathrm{EA}][\mathrm{I}] + k_{-8}[\mathrm{EAI}] \quad \text{(S73)}$$

$$\textsc{eab}: 0 = k_2[\mathrm{EA}][\mathrm{B}] - k_{-2}[\mathrm{EAB}] - k_3[\mathrm{EAB}] + k_{-3}[\mathrm{EQP}] \quad \text{(S74)}$$

$$\textsc{eqp}: 0 = k_3[\mathrm{EAB}] - k_{-3}[\mathrm{EQP}] - k_4[\mathrm{EQP}] + k_{-4}[\mathrm{EQ}][\mathrm{P}] \quad \text{(S75)}$$

$$\textsc{eq}: 0 = k_4[\mathrm{EQP}] - k_{-4}[\mathrm{EQ}][\mathrm{P}] - k_5[\mathrm{EQ}] - k_7[\mathrm{B}][\mathrm{EQ}] + k_{-7}[\mathrm{EQB}] - k_9[\mathrm{EQ}][\mathrm{I}] + k_{-9}[\mathrm{EQI}] \quad \text{(S76)}$$

$$\textsc{fq}: 0 = k_5[\mathrm{EQ}] - k_6[\mathrm{FQ}] + k_{-6}[\mathrm{E}][\mathrm{Q}] \quad \text{(S77)}$$

$$\textsc{eqb}: 0 = k_7[\mathrm{B}][\mathrm{EQ}] - k_{-7}[\mathrm{EQB}] \quad \text{(S78)}$$

$$\textsc{eai}: 0 = k_8[\mathrm{EA}][\mathrm{I}] - k_{-8}[\mathrm{EAI}] \quad \text{(S79)}$$

$$\textsc{eqi}: 0 = k_9[\mathrm{EQ}][\mathrm{I}] - k_{-9}[\mathrm{EQI}] \quad \text{(S80)}$$

$$\textsc{ei}: 0 = k_{10}[\mathrm{E}][\mathrm{I}] - k_{-10}[\mathrm{EI}] \quad \text{(S81)}$$

Jacobian matrix:

$$
\mathbf{J} = \begin{array}{l} \\ \text{E} \\ \text{I} \\ \text{EA} \\ \text{EAB} \\ \text{EQP} \\ \text{EQ} \\ \text{FQ} \\ \text{EQB} \\ \text{EAI} \\ \text{EQI} \\ \text{EI} \end{array}
\begin{array}{c}
\begin{array}{ccccccccccc} \text{E} & \text{I} & \text{EA} & \text{EAB} & \text{EQP} & \text{EQ} & \text{FQ} & \text{EQB} & \text{EAI} & \text{EQI} & \text{EI} \end{array} \\
\left(\begin{array}{ccccccccccc}
1 & . & 1 & 1 & 1 & 1 & 1 & 1 & 1 & 1 & 1 \\
. & 1 & . & . & . & . & . & . & 1 & 1 & 1 \\
k_1[\mathrm{A}] & -k_8[\mathrm{EA}] & J_{3,3} & k_{-2} & . & . & . & . & k_{-8} & . & . \\
. & . & k_2[\mathrm{B}] & J_{4,4} & k_{-3} & . & . & . & . & . & . \\
. & . & . & k_3 & J_{5,5} & k_{-4}[\mathrm{P}] & . & . & . & . & . \\
. & -k_9[\mathrm{EQ}] & . & . & k_4 & J_{6,6} & . & k_{-7} & . & k_{-9} & . \\
k_{-6}[\mathrm{Q}] & . & . & . & . & k_5 & -k_6 & . & . & . & . \\
. & . & . & . & . & k_7[\mathrm{B}] & . & -k_{-7} & . & . & . \\
. & k_8[\mathrm{EA}] & k_8[\mathrm{I}] & . & . & . & . & . & -k_{-8} & . & . \\
. & k_9[\mathrm{EQ}] & . & . & . & k_9[\mathrm{I}] & . & . & . & -k_{-9} & . \\
k_{10}[\mathrm{I}] & k_{10}[\mathrm{E}] & . & . & . & . & . & . & . & . & -k_{-10}
\end{array}\right)
\end{array}
\tag{S82}
$$

$$J_{3,3} = -k_{-1} - k_2[\mathrm{B}] - k_8[\mathrm{I}] \tag{S83}$$

$$J_{4,4} = -k_{-2} - k_3 \tag{S84}$$

$$J_{5,5} = -k_{-3} - k_4 \tag{S85}$$

$$J_{6,6} = -k_{-4}[\mathrm{P}] - k_5 - k_7[\mathrm{B}] - k_9[\mathrm{I}] \tag{S86}$$

10.2.3 Stepwise regression procedure

In the first step, a global data set consisting of varied substrates (IMP, NAD^+) and products (NADH, XMP) was fit to model "S", in which only the microscopic rate constants k_1, k_{-1}, k_5, and k_6 were treated as optimized model parameters, whereas the remaining rate constants were held fixed at values imported from the results of the relevant stopped-flow experiment. The bimolecular association rate constant k_{-6} was held fixed at the arbitrary

value of 1 $\mu M^{-1}s^{-1}$, which approximately corresponds to the diffusion limited association rate constants.[S39] We had verified that, as expected from the theory of steady-state initial rate kinetics,[S40] this arbitrary choice of k_{-6} (1, 10, 100, or 1000 $\mu M^{-1}s^{-1}$) has no effect on the final results. In the second step of the regression procedure, the best-fit values of k_1, k_{-1}, k_5, and k_6 were utilized to fit the initial rate data alternately to models "A", "B", and "C".

10.2.4 Model selection method

A decision in favor of either inhibition model ("A", "B" or "C") was based on the same set of acceptance criteria as was described above in section 6.4.

In particular, each model as evaluated for acceptability using the Akaike Information Criterion (AIC)[S32] and the Bayesian Information Criterion (BIC).[S33] Those models that passed the information-theoretic acceptance test were further scrutinized by the confidence interval method of Bates and Watts.[S28,S29]

10.3 Representative examples

10.3.1 Substrate kinetics of wild-type IMPDH

The following example illustrates the method of determining the microscopic rate constants k_1, k_{-1}, k_5, and k_6 for the wild-type enzyme.

DynaFit input script

```
;-----------------------------------------------------------------------
[task]
   task = fit
   data = rates
   approximation = steady-state
[mechanism]
reaction A + B --> P + Q
```

```
   E + A <==> EA         :    k1    k-1
   EA + B <==> EAB       :    k2    k-2
   EAB <==> EQP          :    k3    k-3
   EQP <==> EQ + P       :    k4    k-4
   EQ ---> FQ            :    k5
   FQ <==> E + Q         :    k6    k-6
   EQ + B <==> EQB       :    k7    k-7
[constants]
   k1 = 1 ?? , k-1 = 10 ??
   k2 = 0.03088 , k-2 = 11.74
   k3 = 139.7 , k-3 = 59.27
   k4 = 124 , k-4 = 1
   k5 = 22.85 ??
   k6 = 100 ?? , k-6 = 1
   k7 = 0.003982 , k-7 = 31.82
[parameters]
   cE1 = 0.02 ?
   cE2 = 0.02 ?
   cE4 = 0.02 ?
   cE5 = 0.02 ?
[responses]
   P = 6220
[data]
   directory ./project/IMPDH/_FINAL/IR/WT/data
   transform EH
variable B
sheet WT-NAD-IMP.csv
graph WT::NAD/IMP
   column 3 | conc E = 1 * cE1, A = 20 | label A= 20
```

```
   column 4 | conc E = 1 * cE1, A = 30 | label A= 30
   column 5 | conc E = 1 * cE1, A = 50 | label A= 50
   column 6 | conc E = 1 * cE1, A = 75 | label A= 75
   column 7 | conc E = 1 * cE1, A = 100 | label A= 100
   column 8 | conc E = 1 * cE1, A = 150 | label A= 150
   column 9 | conc E = 1 * cE1, A = 200 | label A= 200
   column 10 | conc E = 1 * cE1, A = 300 | label A= 300
   column 11 | conc E = 1 * cE1, A = 500 | label A= 500
   column 12 | conc E = 1 * cE1, A = 750 | label A= 750
   column 13 | conc E = 1 * cE1, A = 1000 | label A= 1000 uM
sheet WT-NAD-NADH.csv
graph WT::NAD/NADH
   column 3 | conc E = 1 * cE4, A = 186, P = 28.2 | label P= 28
   column 4 | conc E = 1 * cE4, A = 186, P = 56.4 | label P= 56
   column 5 | conc E = 1 * cE4, A = 186, P = 84.7 | label P= 85
   column 6 | conc E = 1 * cE4, A = 186, P = 112.9 | label P= 113
   column 7 | conc E = 1 * cE4, A = 186, P = 141.1 | label P= 141 uM
sheet WT-NAD-XMP.csv
graph WT::NAD/XMP
   column 3 | conc E = 1 * cE5, A = 205.5, Q = 131.2 | label Q= 131.2
   column 4 | conc E = 1 * cE5, A = 205.5, Q = 262.4 | label Q= 262.4
   column 5 | conc E = 1 * cE5, A = 205.5, Q = 524.8 | label Q= 524.8
   column 6 | conc E = 1 * cE5, A = 205.5, Q = 1049.5 | label Q= 1049.5
   column 7 | conc E = 1 * cE5, A = 205.5, Q = 2099.0 | label Q= 2099.0 uM
variable A
sheet WT-IMP-XMP.csv
graph WT::IMP/XMP
   column 3 | conc E = 1 * cE2, B = 2012, Q = 131.2 | label Q= 131.2
   column 4 | conc E = 1 * cE2, B = 2012, Q = 262.4 | label Q= 262.4
```

```
   column 5 | conc E = 1 * cE2, B = 2012, Q = 524.8 | label Q= 524.8
   column 6 | conc E = 1 * cE2, B = 2012, Q = 1049.5 | label Q= 1049.5
   column 7 | conc E = 1 * cE2, B = 2012, Q = 2099.0 | label Q= 2099.0 uM
[output]
   directory ./project/IMPDH/_FINAL/IR/WT/output/fit-sub-eh
[settings]
{ConfidenceIntervals}
   LevelPercent = 90
{Filter}
   XMax = 6500
{Output}
   ResidualsEPS = n
   XAxisUnit = {/Symbol m}OD/s/{/Symbol m}M
   YAxisLabel = v, {/Symbol m}OD/s
[end]
;----------------------------------------------------------------------
;----------------------------------------------------------------------
```

Results of fit

The best-fit model parameters are listed in Table S17. The “low” and “high” columns represent the lower and upper bounds, respectively, of the 90% confidence level interval.[S28] The parameters labeled as cE1 – cE4 are locally optimized enzyme concentrations in micromolar units. Note that all four microscopic rate constants are well defined by the initial rate experimental data, conditioned on the assumed values of the remaining rate constants imported from the results of the relevant stopped-flow experiment.

The experimental data overlaid on the best-fit regression model is shown in Figure S14. Note that, as expected, the IMP / XMP data set shows a strictly competitive product inhibition pattern in that the Eadie-Hofstee plots intersect on the vertical axis.

Table S17: Best-fit values of regression model parameters in the determination of the microscopic rate constants k_1, k_{-1}, k_5, and k_6 for the wild-type enzyme. For details see text.

#	par/set	initial	final ± std.err.	cv,%	low	high	note
1	k_1	1	0.254 ± 0.015	5.9	0.232	0.28	
2	k_{-1}	10	44.9 ± 5.9	13.1	36.4	55.4	
3	k_5	22.85	18.6 ± 1.3	7.0	16.6	20.9	
4	k_6	100	403 ± 31	7.7	356	458	
5	cE1	0.02	0.00738 ± 0.00035	4.7			
6	cE2	0.02	0.00627 ± 0.00031	4.9			
7	cE4	0.02	0.00763 ± 0.00043	5.6			
8	cE5	0.02	0.00617 ± 0.00033	5.3			

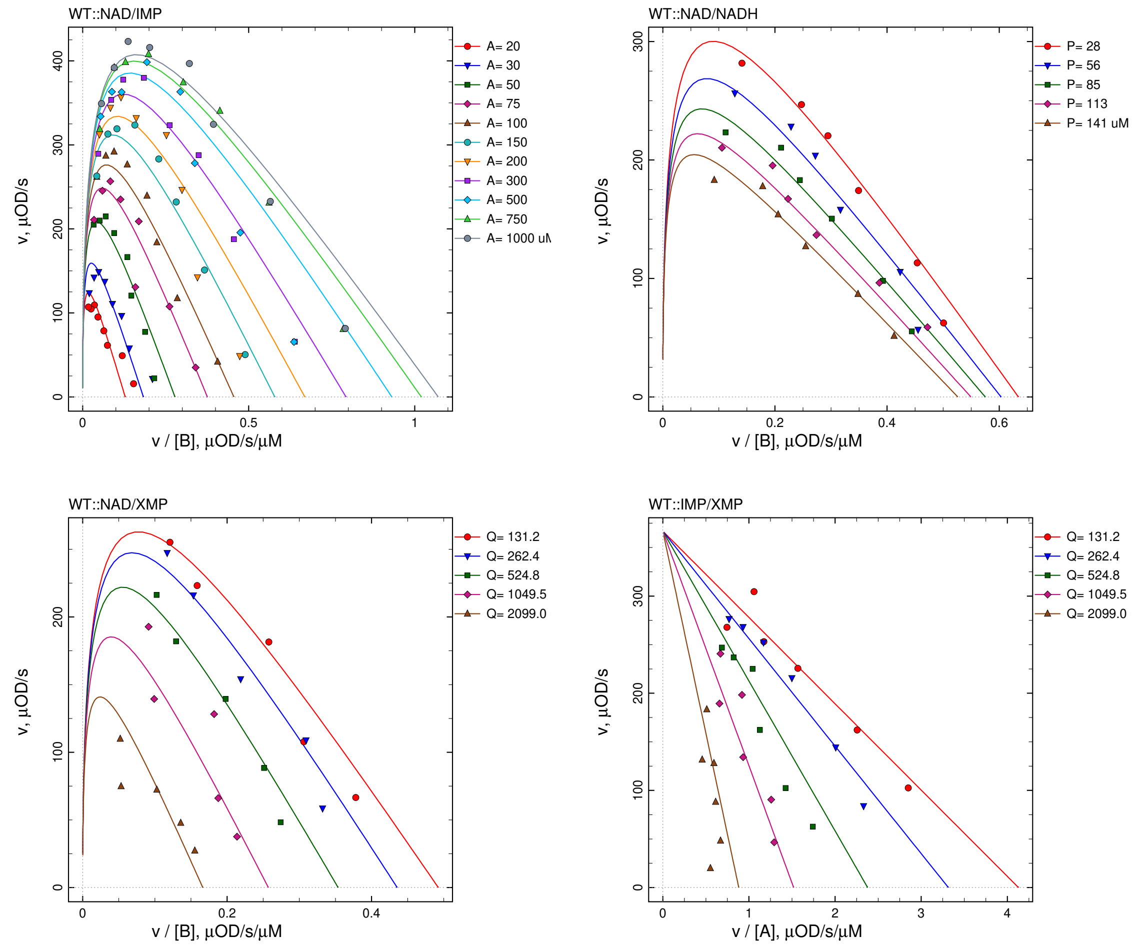

Figure S14: Data vs. model overlay (Eadie-Hofstee plots) resulting from the determination of microscopic rate constants k_1, k_{-1}, k_5, and k_6 for the wild-type enzyme.

10.3.2 Kinetics of wild-type IMPDH inhibited by C91

The following example illustrates the method of determining the microscopic rate constants k_{-8}, k_{-9}, and k_{-10} for the wild-type enzyme inhibited by the compound **C91**. All three fitting models ("A", "B", "C") are examined in their turn and a model selection is performed as described in section 10.2.4.

DynaFit input script

```
;----------------------------------------------------------------------
[task]
   task = fit
   data = rates
   approximation = steady-state
   model = E+EA+EQ ?
[mechanism]
reaction A + B --> P + Q
modifiers I
   E + A <==> EA            :    k1    k-1
   EA + B <==> EAB          :    k2    k-2
   EAB <==> EQP             :    k3    k-3
   EQP <==> EQ + P          :    k4    k-4
   EQ ---> FQ               :    k5
   FQ <==> E + Q            :    k6    k-6
   EQ + B <==> EQB          :    k7    k-7
   EA + I <==> EAI          :    k8    k-8
   EQ + I <==> EQI          :    k9    k-9
   E + I <==> EI            :    k10   k-10
[constants]
   k1 = 0.254 , k-1 = 44.9
```

```
   k2 = 0.03088 , k-2 = 11.74
   k3 = 139.7 , k-3 = 59.27
   k4 = 124 , k-4 = 1
   k5 = 18.6
   k6 = 403 , k-6 = 1
   k7 = 0.003982 , k-7 = 31.82
   k8 = 1 , k-8 = 1 ??
   k9 = 1 , k-9 = 0.1 ??
   k10 = 1 , k-10 = 10 ??
[parameters]
   cE1 = 0.02 ?
[responses]
   P = 6220
[data]
   directory ./project/IMPDH/_FINAL/IR/WT/data
   transform EH
   monitor E, EAI, EQI
;-------------------
variable B
;-------------------
   sheet WT-NAD-C91.csv
graph WT::NAD/C91
   column 2  | conc E = 1 * cE1, A = 200, I = 0  | label I= 0
   column 3  | conc E = 1 * cE1, A = 200, I = 0.025  | label I= 25
   column 4  | conc E = 1 * cE1, A = 200, I = 0.05  | label I= 50
   column 5  | conc E = 1 * cE1, A = 200, I = 0.1  | label I= 100
   column 6  | conc E = 1 * cE1, A = 200, I = 0.2  | label I= 200
   column 7  | conc E = 1 * cE1, A = 200, I = 0.4  | label I= 400 nM
;-------------------
```

```
variable A
;-------------------
   sheet WT-IMP-c91.csv
graph WT::IMP/C91
   column 2  | conc E = 1 * cE1, B = 2000, I = 0  | label I= 0
   column 3  | conc E = 1 * cE1, B = 2000, I = 0.025  | label I= 25
   column 4  | conc E = 1 * cE1, B = 2000, I = 0.05  | label I= 50
   column 5  | conc E = 1 * cE1, B = 2000, I = 0.1  | label I= 100
   column 6  | conc E = 1 * cE1, B = 2000, I = 0.2  | label I= 200
   column 7  | conc E = 1 * cE1, B = 2000, I = 0.4  | label I= 400 nM
[output]
   directory ./project/IMPDH/_FINAL/IR/WT/output/models-C91-eh
[settings]
{ConfidenceIntervals}
   LevelPercent = 90
{Filter}
   XMax = 6500
{Output}
   ResidualsEPS = n
   XAxisUnit = {/Symbol m}M
   YAxisLabel = v, {/Symbol m}OD/s
;----------------------------------------------------------------------
[task]
   task = fit
   data = rates
   approximation = steady-state
   model = EA+EQ ?
[mechanism]
reaction A + B --> P + Q
```

```
modifiers I
   E + A <==> EA           :    k1    k-1
   EA + B <==> EAB         :    k2    k-2
   EAB <==> EQP            :    k3    k-3
   EQP <==> EQ + P         :    k4    k-4
   EQ ---> FQ              :    k5
   FQ <==> E + Q           :    k6    k-6
   EQ + B <==> EQB         :    k7    k-7
   EA + I <==> EAI         :    k8    k-8
   EQ + I <==> EQI         :    k9    k-9
[constants]
   k1 = 0.254 , k-1 = 44.9
   k2 = 0.03088 , k-2 = 11.74
   k3 = 139.7 , k-3 = 59.27
   k4 = 124 , k-4 = 1
   k5 = 18.6
   k6 = 403 , k-6 = 1
   k7 = 0.003982 , k-7 = 31.82
   k8 = 1 , k-8 = 1 ??
   k9 = 1 , k-9 = 0.1 ??
;----------------------------------------------------------------------
[task]
   task = fit
   data = rates
   approximation = steady-state
   model = EQ ?
[mechanism]
reaction A + B --> P + Q
modifiers I
```

```
   E + A <==> EA             :    k1    k-1
   EA + B <==> EAB           :    k2    k-2
   EAB <==> EQP              :    k3    k-3
   EQP <==> EQ + P           :    k4    k-4
   EQ ---> FQ                :    k5
   FQ <==> E + Q             :    k6    k-6
   EQ + B <==> EQB           :    k7    k-7
   EQ + I <==> EQI           :    k9    k-9
[constants]
   k1 = 0.254 , k-1 = 44.9
   k2 = 0.03088 , k-2 = 11.74
   k3 = 139.7 , k-3 = 59.27
   k4 = 124 , k-4 = 1
   k5 = 18.6
   k6 = 403 , k-6 = 1
   k7 = 0.003982 , k-7 = 31.82
   k9 = 1 , k-9 = 0.1 ??
[end]
;----------------------------------------------------------------------
;----------------------------------------------------------------------
```

Results of fit

The experimental data overlaid on the best-fit model curves generated for the regression model "A" is shown in Figure S15. Note that the diagnostic Eadie-Hofstee plots very clearly illustrate that **C91** is strictly *uncompetitive* with respect to IMP and, at the same time, it is *mixed-type noncompetitive* with respect to NAD$^+$. The uncompetitive pattern with respect to IMP signifies that **C91** does not bind to the free enzyme. The mixed-type noncompetitive pattern with respect to NAD$^+$ signifies that **C91** binds to both E•IMP and E-XMP*. The

more conventionally used Lineweaver-Burk plots lead to the same diagnostic conclusions, although arguably with less visual clarity.

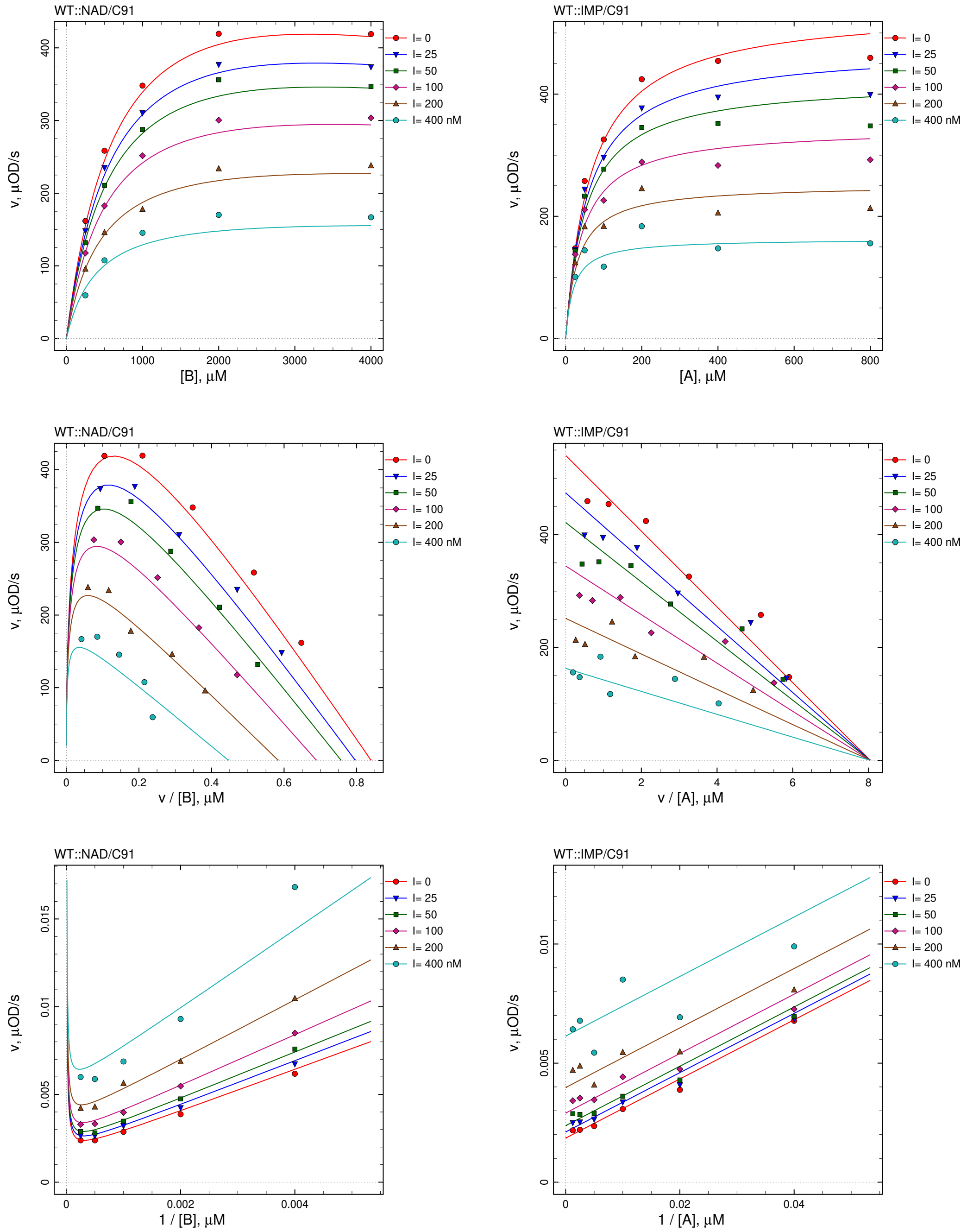

Figure S15: Data vs. model overlay (Eadie-Hofstee plots) resulting from the determination of microscopic rate constants k_{-8}, k_{-9}, and k_{-10} for the wild-type enzyme inhibited by the compound **C91**. *Top row:* Direct plot of original data. *Middle row:* Corresponding Eadie-Hofstee plots. *Bottom row:* Corresponding Lineweaver-Burk plots.

Model discrimination analysis

The model discrimination analysis performed automatically by the DynaFit software package[S14] is summarized below.

Intermediate results

Kinetic constants

Table S18: The best-fit values of kinetic constants for different models

Model	Parameter	Value	StdErr	CV %	Low	High	P(Low) %	P(High) %
A: EQ	k-9	0.0844	0.0047	10.5	0.0769	0.0929	90	90
B: EA+EQ	k-8	0.239	0.1	65.3	0.135	0.687	90	90
	k-9	0.0976	0.0084	13.1	0.0853	0.114	90	90
C: E+EA+EQ	k-8	0.239	0.11	59.4	0.134	0.694	90	90
	k-9	0.0976	0.0087	11.7	0.0852	0.114	90	90
	k-10	1e+007	6.7e+013	884367316.6	4.12	1e+007	90	0

Information-Theoretic Criteria – Full Set

model	n_{P}	SSQ_{r}	ΔAIC	ΔBIC	$w^{(\mathrm{AIC})}$	$w^{(\mathrm{BIC})}$	parameters
A: EQ	2	1.116	5.0	2.8	0.060	0.186	OK
B: EA+EQ	3	1.000	0.0	0.0	0.717	0.738	OK
C: E+EA+EQ	4	1.000	2.3	4.5	0.222	0.076	FAIL:UB

Parameter failures – Legend

:LB ... Missing lower bound for at least one parameter

:UB ... Missing upper bound

:UB/LB ... Upper-to-lower bound ratio too large

:CV ... Coefficient of variation too large

Final results

Information-Theoretic Criteria – Reduced Set

model	n_P	SSQ_r	ΔAIC	ΔBIC	$w^{(AIC)}$	$w^{(BIC)}$	status
A: EQ	2	1.116	5.0	2.8	0.078	0.201	OK
B: EA+EQ	3	1.000	0.0	0.0	0.922	0.799	OK

Model Discrimination Analysis – Conclusions

2 Different candidate models pass all acceptance criteria:

task no.	model ID	n_P	SSQ_r	Δ AIC	Δ BIC
2	B: EA+EQ	3	1	0	0
3	A: EQ	2	1.1156	4.95138	2.76172

Acceptable parameters

Model	Parameter	Value	Low	High
A: EQ	k-9	0.0844	0.0769	0.0929
B: EA+EQ	k-8	0.239	0.135	0.687
	k-9	0.0976	0.0853	0.114

The results show that either model "A" (inhibitor binding to E-XMP* only) or model "B" (binding to both E•IMP and E-XMP*) explain the experimental data approximately equally well and nominally both are acceptable. However, the Akaike weight parameter w_{AIC}[S32] is greater than 0.90 for model "B", corresponding to greater than 90% statistical probability that **C91** does bind to both enzyme forms.

In contrast, model "C", according to which the inhibitor is additionally binding also the free enzyme E, is clearly disqualified for two independent reasons. First, the relative residual sum of squares SSQ_r is identical for models "B" and "C", while model "B" has fewer adjustable parameters. Thus, model "C" is rejected by the Occam's Razor rule, also reflected in the

fact that $\Delta AIC << 5$ for model "C".[S32] Secondly, the upper limit of the confidence interval for k_{-10} could not be determined at the 90% confidence level.

The overall conclusion from this auto-generated model discrimination analysis is that the inhibitor **C91** binds to both E•IMP and E-XMP*, but it does not bind to the free enzyme E. Similar analyses were performed for all remaining combinations of enzymes (wild-type or L413A mutant) and inhibitors (**A110**, **C91**, **P131**, **D67**, and **Q21**). The results are summarized in the main manuscript.

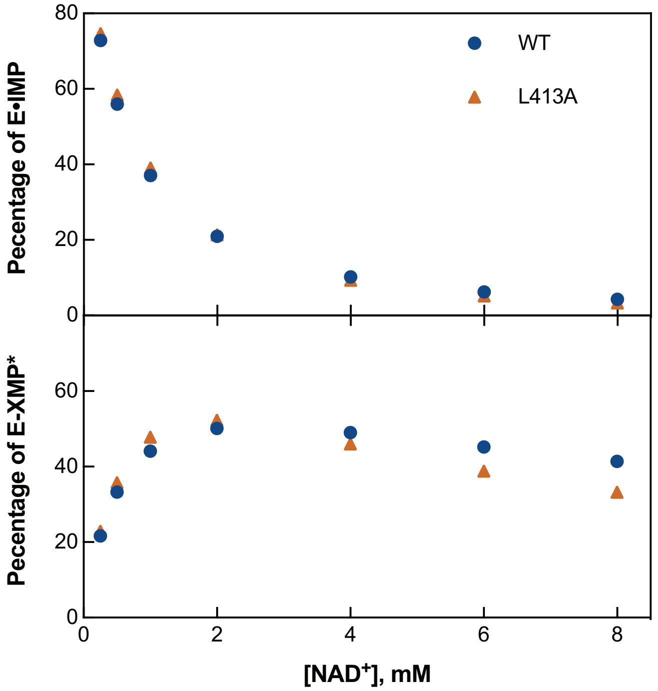

Figure S16: **The accumulation of E•IMP and E-XMP***

References

(S1) Makowska-Grzyska, M.; Kim, Y.; Maltseva, N.; Osipiuk, J.; Gu, M.; Zhang, M.; Mandapati, K.; Gollapalli, D. R.; Gorla, S. K.; Hedstrom, L.; Joachimiak, A. A novel cofactor-binding mode in bacterial IMP dehydrogenases explains inhibitor selectivity. *J. Biol. Chem.* **2015**, *290*, 5893–5911.

(S2) Kim, Y.; Babnigg, G.; Jedrzejczak, R.; Eschenfeldt, W. H.; Li, H.; Maltseva, N.; Hatzos-Skintges, C.; Gu, M.; Makowska-Grzyska, M.; Wu, R.; An, H.; Chhor, G.; Joachimiak, A. High-throughput protein purification and quality assessment for crystallization. *Methods* **2011**, *55*, 12–28.

(S3) Rosenbaum, G.; Alkire, R. W.; Evans, G.; Rotella, F. J.; Lazarski, K.; Zhang, R. G.; Ginell, S. L.; Duke, N.; Naday, I.; Lazarz, J.; Molitsky, M. J.; Keefe, L.; Gonczy, J.; Rock, L.; Sanishvili, R.; Walsh, M. A.; Westbrook, E.; Joachimiak, A. The Structural Biology Center 19ID undulator beamline: facility specifications and protein crystallographic results. *J Synchrotron Radiat* **2006**, *13*, 30–45.

(S4) Minor, W.; Cymborowski, M.; Otwinowski, Z.; Chruszcz, M. HKL-3000: the integration of data reduction and structure solution–from diffraction images to an initial model in minutes. *Acta Crystallogr D Biol Crystallogr.* **2006**, *62*, 859–66.

(S5) Vagin, A.; Teplyakov, A. Molecular replacement with MOLREP. *Acta Crystallogr D Biol Crystallogr* **2010**, *66*, 22–5.

(S6) Murshudov, G. N.; Vagin, A. A.; Dodson, E. J. Refinement of macromolecular structures by the maximum-likelihood method. *Acta Crystallogr D Biol Crystallogr* **1997**, *53*, 240–55.

(S7) Emsley, P.; Cowtan, K. Coot: model-building tools for molecular graphics. *Acta Crystallogr D Biol Crystallogr* **2004**, *60*, 2126–32.

(S8) Adams, P. D.; Afonine, P. V.; Bunkoczi, G.; Chen, V. B.; Davis, I. W.; Echols, N.; Headd, J. J.; Hung, L. W.; Kapral, G. J.; Grosse-Kunstleve, R. W.; McCoy, A. J.; Moriarty, N. W.; Oeffner, R.; Read, R. J.; Richardson, D. C.; Richardson, J. S.; Terwilliger, T. C.; Zwart, P. H. PHENIX: a comprehensive Python-based system for macromolecular structure solution. *Acta Crystallogr D Biol Crystallogr* **2010**, *66*, 213–21.

(S9) Laskowski, R. A.; MacArthur, M. W.; Moss, D. S.; Thornton, J. M. PROCHECK: a program to check the stereochemical quality of protein structures. *Journal of Applied Crystallography* **1993**, *26*, 283–291.

(S10) Davis, I. W.; Leaver-Fay, A.; Chen, V. B.; Block, J. N.; Kapral, G. J.; Wang, X.; Murray, L. W.; Arendall, r., W. B.; Snoeyink, J.; Richardson, J. S.; Richardson, D. C. MolProbity: all-atom contacts and structure validation for proteins and nucleic acids. *Nucleic Acids Res* **2007**, *35*, W375–83.

(S11) Pettersen, E. F.; Goddard, T. D.; Huang, C. C.; Couch, G. S.; Greenblatt, D. M.; Meng, E. C.; Ferrin, T. E. UCSF Chimera – a visualization system for exploratory research and analysis. *J. Comput. Chem.* **2004**, *25*, 1605–1612.

(S12) Kuzmič, P.; Elrod, K. C.; Cregar, L. M.; Sideris, S.; Rai, R.; Janc, J. W. High-throughput screening of enzyme inhibitors: Simultaneous determination of tight-binding inhibition constants and enzyme concentration. *Anal. Biochem.* **2000**, *286*, 45–50.

(S13) Kuzmič, P. Program DYNAFIT for the analysis of enzyme kinetic data: Application to HIV proteinase. *Anal. Biochem.* **1996**, *237*, 260–273.

(S14) Kuzmič, P. DynaFit - A software package for enzymology. *Meth. Enzymol.* **2009**, *467*, 247–280.

(S15) Wei, Y.; Kuzmič, P.; Yu, R.; Modi, G.; Hedstrom, L. Inhibition of inosine-5'-monophosphate dehydrogenase from *Bacillus anthracis*: mechanism revealed by pre-steady-state kinetics. *Biochemistry* **2016**, *55*, 5279–88.

(S16) Johnson, K. A. Fitting enzyme kinetic data with KinTek Global Kinetic Explorer. *Meth. Enzymol.* **2009**, *467*, 601–626.

(S17) Johnson, K. A.; Simpson, Z. B.; Blom, T. FitSpace Explorer: An algorithm to evaluate multidimensional parameter space in fitting kinetic data. *Anal. Biochem.* **2009**, *387*, 30–41.

(S18) Johnson, K. A.; Simpson, Z. B.; Blom, T. Global Kinetic Explorer: A new computer program for dynamic simulation and fitting of kinetic data. *Anal. Biochem.* **2009**, *387*, 20–29.

(S19) Kuzmič, P. A generalized numerical approach to steady-state enzyme kinetics: Applications to protein kinase inhibition. *Biochim. Biophys. Acta* **2010**, *1804*, 635–641.

(S20) Morrison, J. F. Kinetics of the reversible inhibition of enzyme-catalysed reactions by tight-binding inhibitors. *Biochim. Biophys. Acta* **1969**, *185*, 269–286.

(S21) Cha, S. Tight-binding inhibitors. I. Kinetic behavior. *Biochem. Pharmacol.* **1975**, *24*, 2177–2185.

(S22) Williams, J. W.; Morrison, J. F. The kinetics of reversible tight-binding inhibition. *Meth. Enzymol.* **1979**, *63*, 437–467.

(S23) Modi, G.; Marqus, G. M.; Vippila, M. R.; Gollapalli, D. R.; Kim, Y.; Manna, A. C.; Chacko, S.; Maltseva, N.; Wang, X.; Cullinane, R. T.; Zhang, Y.; Kotler, J. L. M.; Kuzmic, P.; Zhang, M.; Lawson, A. P.; Joachimiak, A.; Cheung, A.; Snider, B. B.; Rothstein, D. M.; Cuny, G. D.; Hedstrom, L. The Enzymatic Activity of Inosine 5'-

Monophosphate Dehydrogenase May Not Be a Vulnerable Target for Staphylococcus aureus Infections. *ACS Infect. Dis.* **2021**, *7*, 3062–3076.

(S24) Pettersen, E. F.; Goddard, T. D.; Huang, C. C.; Meng, E. C.; Couch, G. S.; Croll, T. I.; Morris, J. H.; Ferrin, T. E. UCSF ChimeraX: Structure visualization for researchers, educators, and developers. *Protein Sci* **2021**, *30*, 70–82.

(S25) Kim, Y.; Makowska-Grzyska, M.; Gorla, S. K.; Gollapalli, D. R.; Cuny, G. D.; Joachimiak, A.; Hedstrom, L. Structure of *Cryptosporidium* IMP dehydrogenase bound to an inhibitor with in vivo antiparasitic activity. *Acta Crystallogr F Struct Biol Commun* **2015**, *71*, 531–8.

(S26) Cleland, W. The kinetics of enzyme-catalyzed reactions with two or more substrates or products: I. Nomenclature and rate equations. *Biochim. Biophys. Acta* **1963**, *67*, 104–137.

(S27) Cook, P.; Cleland, W. *Enzyme Kinetics and Mechanism*; Garland Science Publishing: New York, 2007.

(S28) Bates, D. M.; Watts, D. G. *Nonlinear Regression Analysis and its Applications*; Wiley: New York, 1988.

(S29) Watts, D. G. Parameter estimation from nonlinear models. *Meth. Enzymol.* **1994**, *240*, 24–36.

(S30) Beechem, J. M. Global analysis of biochemical and biophysical data. *Meth. Enzymol.* **1992**, *210*, 37–54.

(S31) Cha, S. A simple method for derivation of rate equations for enzyme-catalyzed reactions under the rapid equilibrium assumption or combined assumptions of equilibrium and steady state. *J. Biol. Chem.* **1968**, *243*, 820–825.

(S32) Burnham, K. B.; Anderson, D. R. *Model Selection and Multimodel Inference: A Practical Information-Theoretic Approach*, 2nd ed.; Springer-Verlag: New York, 2002.

(S33) Myung, J. I.; Pitt, M. A. Model comparison methods. *Meth. Enzymol.* **2004**, *383*, 351–366.

(S34) Getov, I.; Petukh, M.; Alexov, E. SAAFEC: Predicting the Effect of Single Point Mutations on Protein Folding Free Energy Using a Knowledge-Modified MM/PBSA Approach. *Int. J. Mol. Sci.* **2016**, *17*, 512.

(S35) Hindmarsh, A. C. LSODE and LSODI, two new initial value ordinary differential equation solvers. *ACM SIGNUM Newslett.* **1980**, *15*, 10–11.

(S36) Hindmarsh, A. C. In *Scientific Computing*; Stepleman, R. S., Carver, M., Peskin, R., Ames, W. F., Vichnevetsky, R., Eds.; North Holland: Amsterdam, 1983; pp 55–64.

(S37) Brooks, I.; Watts, D.; Soneson, K.; Hensley, P. Determining confidence intervals for parameters derived from analysis of equilibrium analytical ultracentrifugation data. *Meth. Enzymol.* **1994**, *240*, 459–78.

(S38) Fersht, A. *Enzyme structure and mechanism*, 2nd ed.; W. H. Freeman: New York, 1985.

(S39) Ref. S38, p. 150.

(S40) Segel, I. H. *Enzyme Kinetics*; Wiley: New York, 1975.
